# 3D Facial Normotypes for Maxillofacial Surgical Planning

**DOI:** 10.3390/jcm15197518

**Published:** 2026-09-27

**Authors:** Federica Marcolin, Ylenia Gugliotta, Armando La Rosa, Michela Moschella, Alice Tommolini, Enrico Vezzetti, Fabio Roccia, Fabrizio Ferretti, Elena Carlotta Olivetti, Guglielmo Ramieri

**Affiliations:** 1 Department of Management and Production Engineering, Politecnico di Torino, 10129 Turin, Italy; federica.marcolin@polito.it (F.M.); s323793@studenti.polito.it (A.L.R.); s319456@studenti.polito.it (M.M.); s331967@studenti.polito.it (A.T.); enrico.vezzetti@polito.it (E.V.); elena.olivetti@polito.it (E.C.O.); 2 Maxillo–Facial Surgery Unit, Department of Surgical Sciences, AOU Città della Salute e della Scienza, University of Turin, 10126 Turin, Italy; ylenia.gugliotta@unito.it (Y.G.); guglielmo.ramieri@unito.it (G.R.); 3 Department of Mechanical and Aerospace Engineering, Politecnico di Torino, 10129 Turin, Italy

**Keywords:** orthognathic surgery, 3D Face Analysis, craniofacial anthropometry, facial landmarks, soft tissue morphology, sexual dimorphism, pre-operative planning, multivariate statistical analysis, facial normotypes

## Abstract

**Background:** Preoperative planning in maxillofacial and orthognathic surgery requires anatomical references to guide the definition of functional and aesthetic treatment objectives. Currently available planning tools do not provide quantitative, clinically interpretable three-dimensional morphological targets that are tailored to patient’s sex- and geography-specific characteristics. This study proposes a methodological pipeline for the development of sex-specific 3D facial normotypes in a population of healthy, normal-weight Caucasian young adults to serve as a reference for maxillofacial surgery planning. **Methods:** The approach is based on the integration of validated anthropometric references with three-dimensional data acquired using 3D scanners. Semi-automatic landmarking was performed on the acquired faces, and a comprehensive set of literature-acknowledged morphometric features and related reference ranges were extracted; a multivariate statistical analysis was applied. **Results:** The final collected database comprised 109 Caucasian subjects (70 females, 64.2%) that served as a quantitative basis for designing a female and male facial normotype in the form of 3D models. The developed normotypes showed high agreement with the literature-based reference ranges, reaching 95% for the male model and 91% for the female model. **Conclusions:** This study proposes a methodological approach integrating classical anthropometry with 3D digital modeling. The resulting normotypes represent consistent morphological face models and provide a quantitative and morphologically interpretable reference framework. This framework may support the future development of interactive tools and quantitative assessment metrics for surgical planning. However, its clinical applicability has not yet been validated, and further studies are required to assess its reliability and potential utility in surgical planning.

## 1. Introduction

Pre-operative planning in maxillofacial and orthognathic surgery plays a central role in the management of dento-skeletal deformities, as it directly influences both functional and aesthetic outcomes. Procedures such as bilateral sagittal osteotomy (BSSO) and Le Fort I osteotomy (LFI) result in significant changes in skeletal structures and associated soft tissues [1]. Whilst functional objectives are guided by well-established occlusal and skeletal parameters, the definition of a morphologically harmonious and proportionate facial outcome remains poorly standardized. The human face is, in fact, the result of a complex interaction between soft tissues and underlying bony structures, and alterations to this balance can have significant clinical and social consequences.

The introduction of three-dimensional (3D) imaging techniques and advanced surgical simulation tools has significantly improved the accuracy of pre-operative planning. These systems allow for detailed geometric modelling and the simulation of surgical movements on hard tissues. However, despite these advances, the identification of an appropriate aesthetic target and the effects of hard tissue displacements on soft tissues remain largely dependent on clinical experience. Current approaches frequently rely on qualitative assessments or two-dimensional references, which are unable to fully represent the three-dimensional complexity of facial morphology.

Several approaches to 3D facial modelling have been proposed in the literature. Statistical deformable models, such as those introduced by Blanz and Vetter [2], enable the parametric synthesis of realistic faces and are widely used in computer graphics and computer vision. Other studies have focused on facial morphology analysis for the identification of dysmorphic patterns [3], primarily for diagnostic purposes. Furthermore, registration techniques such as the Iterative Closest Point (ICP) algorithm are fundamental tools for the alignment and comparison of three-dimensional surfaces [4]. Despite their scientific soundness, these contributions were not designed with the aim of defining clinically interpretable morphological targets for orthognathic surgical planning.

In this context, craniofacial anthropometry provides a standardised framework for the quantitative description of human morphology, with direct applications in maxillofacial and orthognathic surgery to establish objective normotypical criteria. In recent decades, the discipline has evolved from two-dimensional approaches based on direct measurements to three-dimensional methodologies, with marked improvements in terms of accuracy and clinical reproducibility. This development builds on the pioneering work of Leslie G. Farkas [5], who established a rigorous and systematic definition of landmarks and normotypical datasets stratified by sex, age and ethnicity. These references were further expanded by subsequent multicentre studies [6], enabling comparisons across different populations and establishing the discipline as a reference standard for facial analysis. This framework is particularly relevant in the context of three-dimensional facial modeling, where the integration of traditional bidimensional anthropometric knowledge with 3D geometric representations (in terms of curves, surfaces or 3D distances and angles) is essential to define clinically meaningful and quantitatively interpretable morphological targets.

The introduction of digital technologies, such as stereophotogrammetry and 3D scanners, marked a major turning point, enabling the non-invasive acquisition of the facial surface and reducing operator-dependent variability. In this context, the contribution of Swennen et al. [7] was instrumental in adapting cephalometric protocols to the three-dimensional environment, whilst large-scale databases such as the 3D Facial Norms [8] have made it possible to define statistically robust normotypical ranges. A key element within this digital transition was the adoption of landmarks, which ensure comprehensive and univocal coverage and mapping of all facial regions.

In light of these considerations, a clear gap in the literature emerges. Currently, there are no three-dimensional normotypical models available that are designed as quantitative and clinically interpretable references for pre-operative planning. In particular, there is a lack of models specific to sex, age and geographical provenance, derived from validated anthropometric measurements and characterised by proportional consistency. Existing approaches provide statistical representations lacking direct clinical applicability or focus on classification tasks without defining an explicit morphological target. Importantly, however, population-based normative models should not be interpreted as defining an “ideal” facial morphology for an individual patient. Normative values represent population-based references rather than prescriptive treatment goals, and individual surgical objectives should remain tailored to patient-specific anatomical, functional, and aesthetic characteristics and expectations [9]. Nevertheless, the availability of quantitatively defined and morphologically consistent normotypes may provide an objective reference against which individual facial morphology can be assessed, interpreted, and incorporated into patient-specific surgical planning. To address this gap, the present study proposes the development of sex-specific three-dimensional facial normotypes for a population of healthy, normal-weight young Caucasian adults.

## 2. Materials and Methods

**Figure 1:**
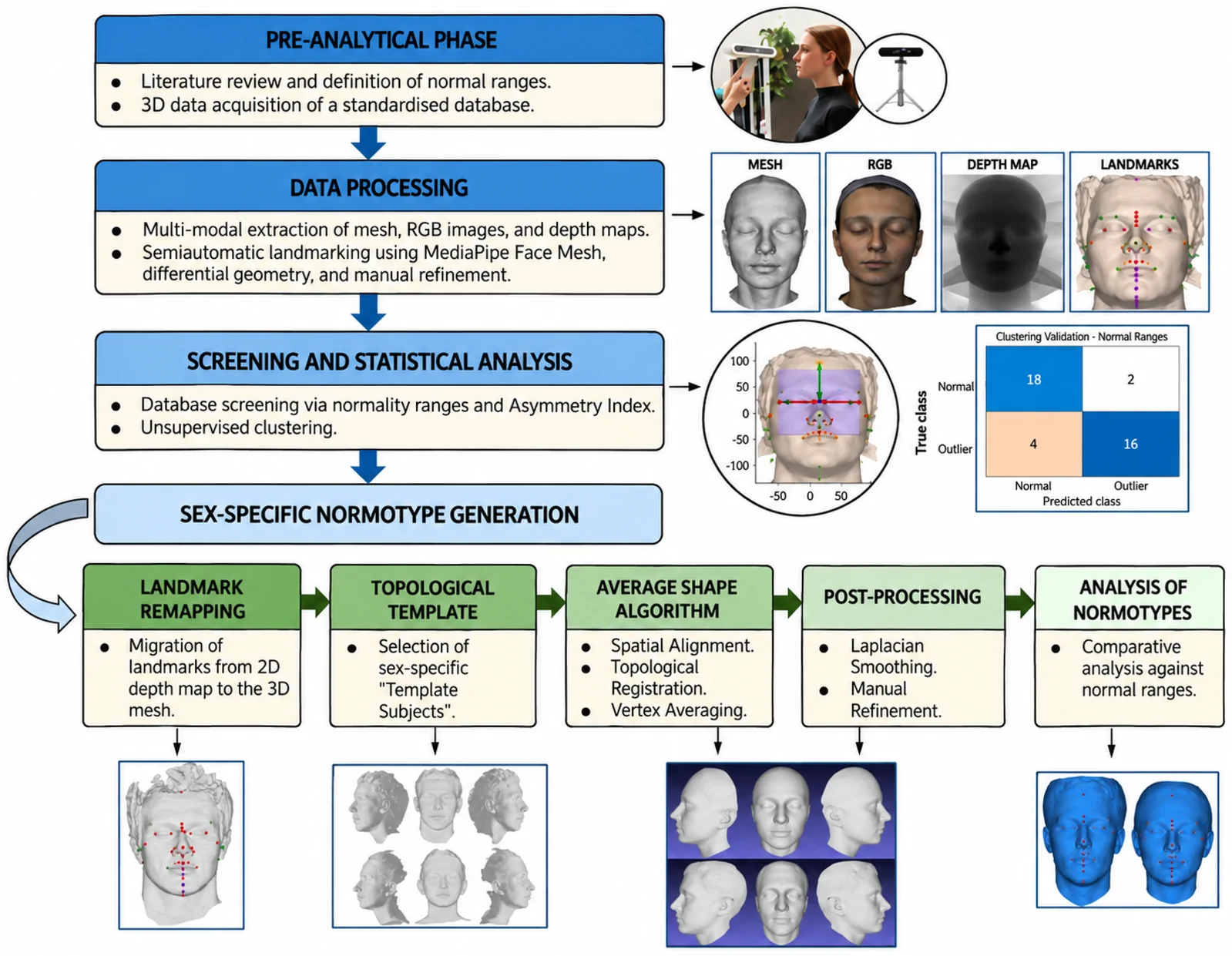
Methodological workflow for the generation of sex-specific 3D facial normotypes, from literature review to the generation and analysis of final 3D models.

The proposed approach, integrating both computational and clinical components for the development of three-dimensional facial normotypes, is structured as a sequential pipeline (Figure 1), primarily implemented in MATLAB R2025b (The MathWorks, Inc., Natick, MA, USA), with Python 3.12.x (Python Software Foundation, Wilmington, DE, USA) utilised for facial landmark extraction via the MediaPipe Face Mesh framework (version 0.10.35, Google LLC, Mountain View, CA, USA).

The methodological workflow comprised two complementary data collection phases. First, literature-derived anthropometric data were collected to identify clinically relevant facial landmarks and measurements and to define sex-specific normotypical reference ranges. Second, 3D facial data were collected to construct the experimental database used for the development of the facial normotypes. Subsequently, a standardised framework was implemented for the acquisition and processing of 3D facial data, comprising the construction of the experimental database, semi-automatic landmark identification, and spatial alignment. Finally, a multivariate statistical analysis was used to investigate morphological variability and derive sex-specific normotypes.

### 2.1. Inclusion and Exclusion Criteria

The same eligibility criteria were applied both to the literature review to select the reference studies used to define the normotypical ranges, and to the recruitment of subjects for the experimental database construction, in order to ensure consistency between the literature-derived reference population and the experimental population.

The inclusion criteria were:

- Caucasian ethnicity;
- Age between 18 and 30 years;
- Body mass index (BMI) within the normal range (18.5–24.9 kg/m^2^).

The exclusion criteria were:

- Craniofacial pathologies potentially affecting facial morphology or the soft-tissue facial profile;
- Previous maxillofacial or facial surgery;
- History of facial trauma;

For the experimental database, eligibility and the absence of the aforementioned conditions were assessed through anamnesis. For the selection of literature-based references, studies were considered eligible when the characteristics of the investigated population, as reported in the respective articles, were consistent with these criteria. The focus on the Caucasian population reflects the need to maintain consistency with the clinical target of this study, as this ethnic group is predominant among patients being treated at the Division of Maxillofacial Surgery.

### 2.2. Literature-Based Landmark and Morphometric Measure Selection, and Identification of Normotypical Ranges

The literature review focused on identifying quantitative facial measurements that were applicable to the patient population treated at the Division of Maxillofacial Surgery, Molinette Hospital (A.O.U. Città della Salute e della Scienza, University of Turin), with a particular focus on patients undergoing orthognathic surgery. Therefore, only studies reporting data from populations meeting the eligibility criteria described in Section 2.1 were included.

After all potential measurements and landmarks from the literature had been collected, the entire dataset was subject to a critical review by maxillofacial surgeons. First, redundancy was eliminated by discarding the landmarks and measurements that provided overlapping morphological descriptions or were clinically less significant. Secondly, the set was optimized for the digital environment; only landmarks and measurements that could be identified from the3D facial scans and digital models used in the study were retained.

Relying on the selected set of measurements, sex-specific normotypical ranges were defined by integrating literature-based references, ensuring both statistical robustness and relevance for maxillofacial applications. In this phase, a distinction was made between linear and angular measurements, for which normotypical ranges were defined, and facial proportions and indices, which were treated separately. The calculation of normotypical ranges was carried out according to a rigorous protocol designed to ensure maximum statistical and clinical robustness.

In particular, the adopted procedure allows for the definition of normotypical intervals representative of physiological variability, which serve as a quantitative reference for the interpretation of facial morphology and for the subsequent validation of the proposed normotypes. To harmonize the quantitative data derived from different literature sources, a standardized procedure was applied to the reported reference values. Regarding linear and angular measures, for studies reporting only mean and standard deviation, the minimum and maximum values were estimated in accordance with Farkas’s methodological approach, calculating the interval as mean±2timesstandarddeviation; this method allows for the inclusion of approximately 95% of the variability in the reference population, defining statistically robust boundaries for the morphological normotype [10]. Once the extreme values for each source were obtained, the final normotypical range for each measurement was identified by selecting the absolute minimum and absolute maximum across all eligible literature references. For facial proportions and indices, literature-acknowledged reference values were used [10,11,12]. This conservative approach was aimed at preserving the physiological variants across Caucasian sub-populations determining inherent geographical heterogeneity.

### 2.3. Construction of the Experimental Database

**Figure 2:**
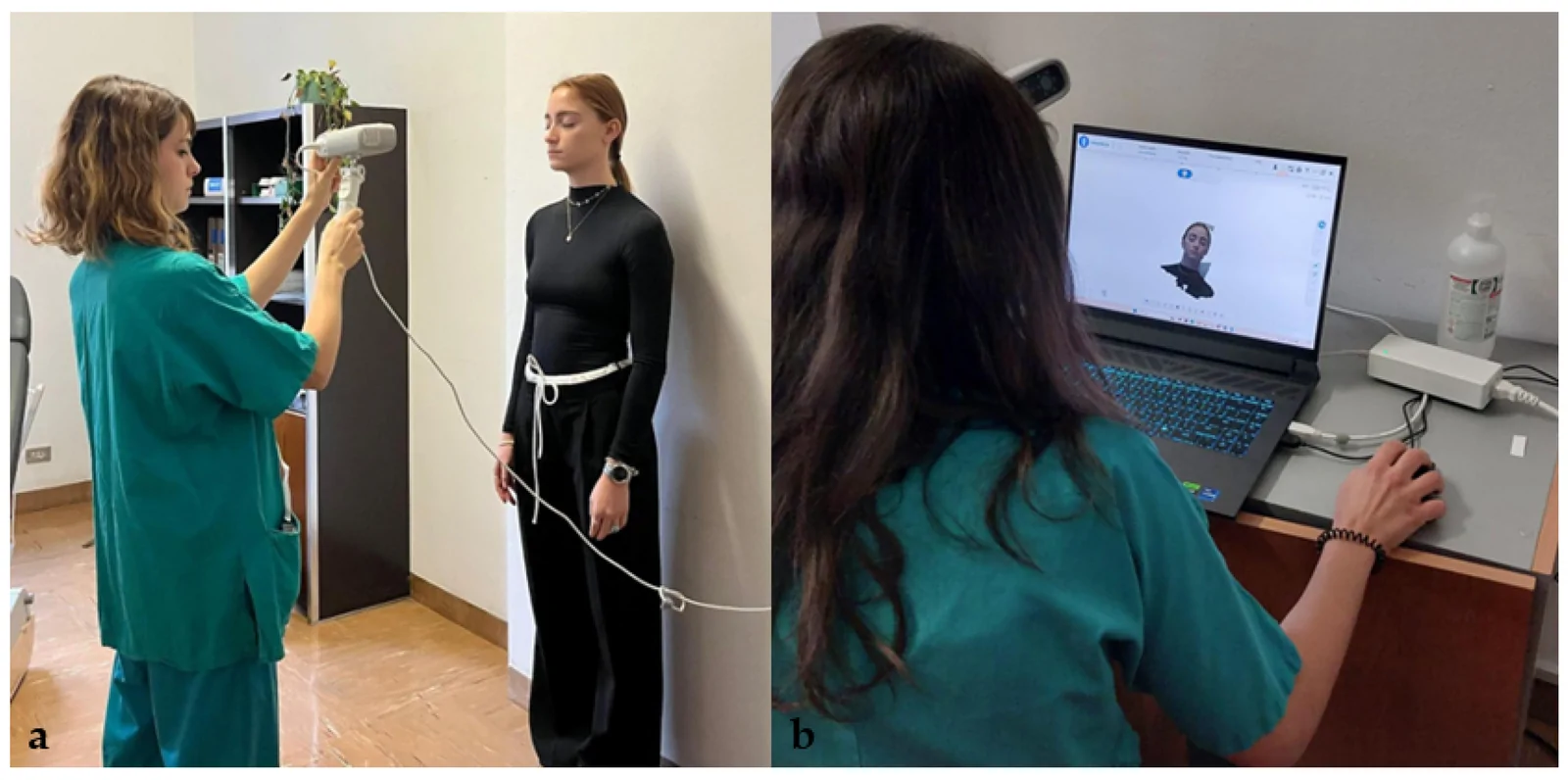
3D acquisition using a MetiSmile scanner. (**a**) The subject remains standing upright, with their head in NHP and eyes closed, while the operator moves the device manually. (**b**) After acquisition, scan quality is verified, and any inadvertently captured areas are removed.

3D facial data of 139 Caucasian subjects (55 males, 84 females) aged between 18 and 30, selected using the same inclusion criteria described in Section 2.1, were collected at Molinette Hospital between 11 May and 11 June 2026. The so-created database is aimed at building the normotype facial models. The scans were acquired using a MetiSmile 3D facial scanner (SHINING 3D Tech Co., Ltd., Hangzhou, Zhejiang, China) [13] under controlled conditions. To ensure maximum reproducibility and reduce inter-operator variability, all scans were acquired by a trained operator in a controlled, indirect lighting environment. Subjects were positioned in an upright posture, maintaining a Natural Head Position (NHP) with a neutral expression and relaxed facial muscles; during the procedure, participants were asked to keep their eyes closed to prevent involuntary head movements caused by visually following the scanner (Figure 2).

All participants provided written informed consent for the use of their demographic and biometric data for research purposes. The study protocol was reviewed and approved by the Clinical Research Office of the A.O.U. Città della Salute e della Scienza di Torino (Protocol No. 00012/2026, approved on 8 May 2026). The study was conducted in accordance with the principles of the Declaration of Helsinki and applicable institutional regulations. All data were stored on digital systems owned and managed by the A.O.U. Città della Salute e della Scienza di Torino and were accessible exclusively to the investigators directly involved in the study. Appropriate measures were adopted to ensure data confidentiality and security throughout the study period.

### 2.4. Landmarking

**Figure 3:**
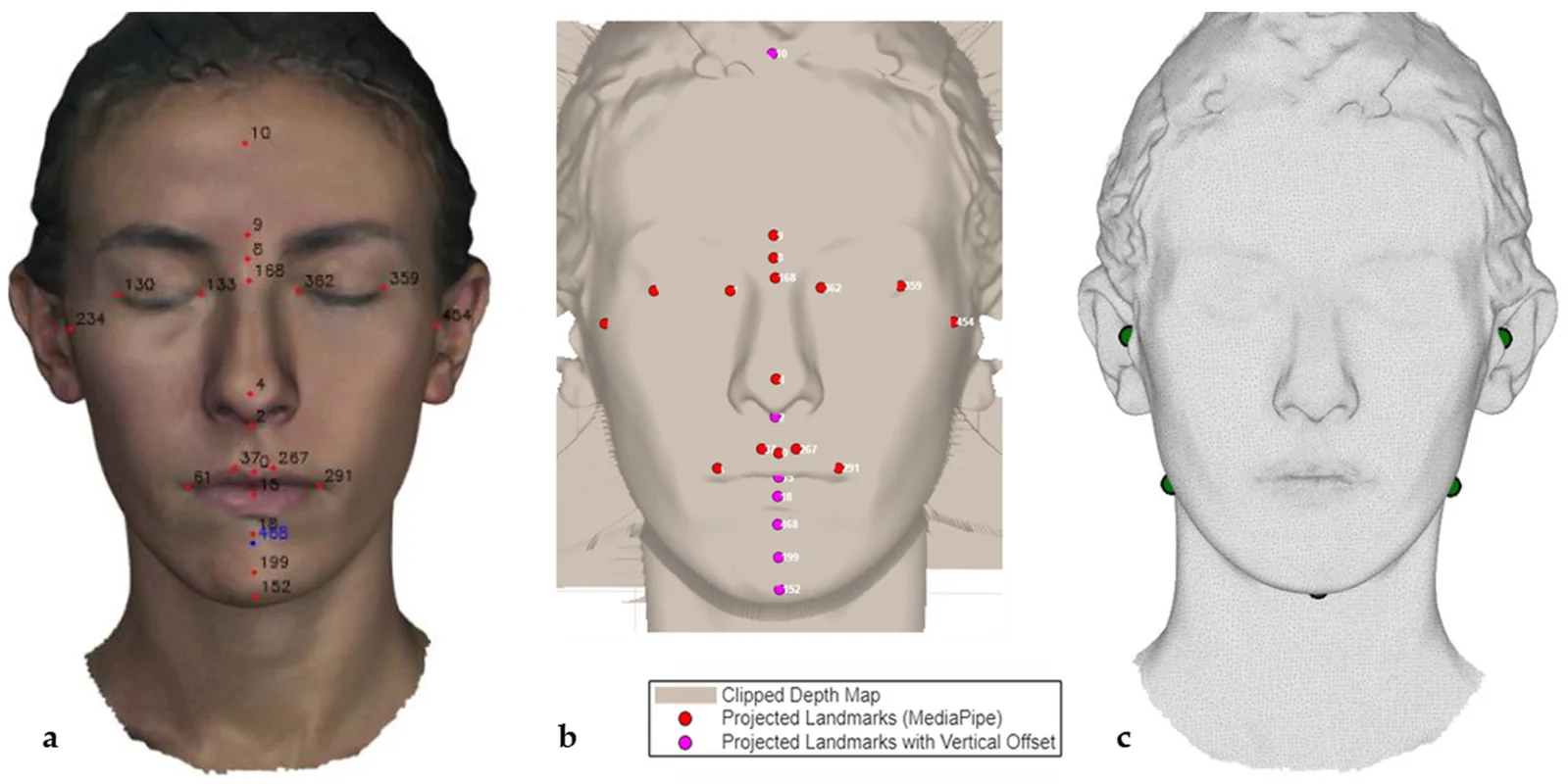
Semi-automatic landmarking workflow. (**a**) Automated 2D extraction via MediaPipe; (**b**) 3D projection of 22 landmarks onto the depth map with vertical offset corrections; (**c**) manual positioning of the five remaining landmarks on the 3D mesh.

A multi-stage semi-automatic landmarking procedure was implemented (Figure 3).

The MediaPipe Face Mesh framework [14] was used to extract 22 2D landmarks from the RGB image, which were subsequently projected into the aligned 3D model. Facial midline landmarks were automatically adjusted to be vertically aligned (Table 1). Each detected landmark corresponds to a predefined MediaPipe Face Mesh index, ensuring full reproducibility of the mapping between the algorithmic output and the anthropometric definition of each point. The corresponding indices are: stomion (15), pronasale (4), subnasale (2), nasion (8), glabella (9), trichion (10), labiale inferius (18), labiale superius (0), gnathion (152), sellion (168), pogonion (199), left/right exocanthion (359, 130), left/right endocanthion (362, 133), left/right crista philtri (267, 37), left/right zygion (454, 234), left/right cheilion (291, 61). The sublabial was defined as a custom landmark (index 468), computed as the midpoint between MediaPipe landmarks 18 (chosen as labiale inferius) and 200, ensuring a stable representation along the facial midline. This approach was adopted because MediaPipe does not provide a landmark that accurately corresponds to the anthropometric sublabial point. Therefore, the midpoint between landmarks 18 and 200 was used as an approximation of the true sublabial landmark. The consistency of this approximation was preliminarily assessed on a subsample of 30 subjects, including 15 males and 15 females, by the expert surgical team, who verified that the computed point provided a satisfactory approximation of the anatomically identified sublabial landmark.

**Figure 4:**
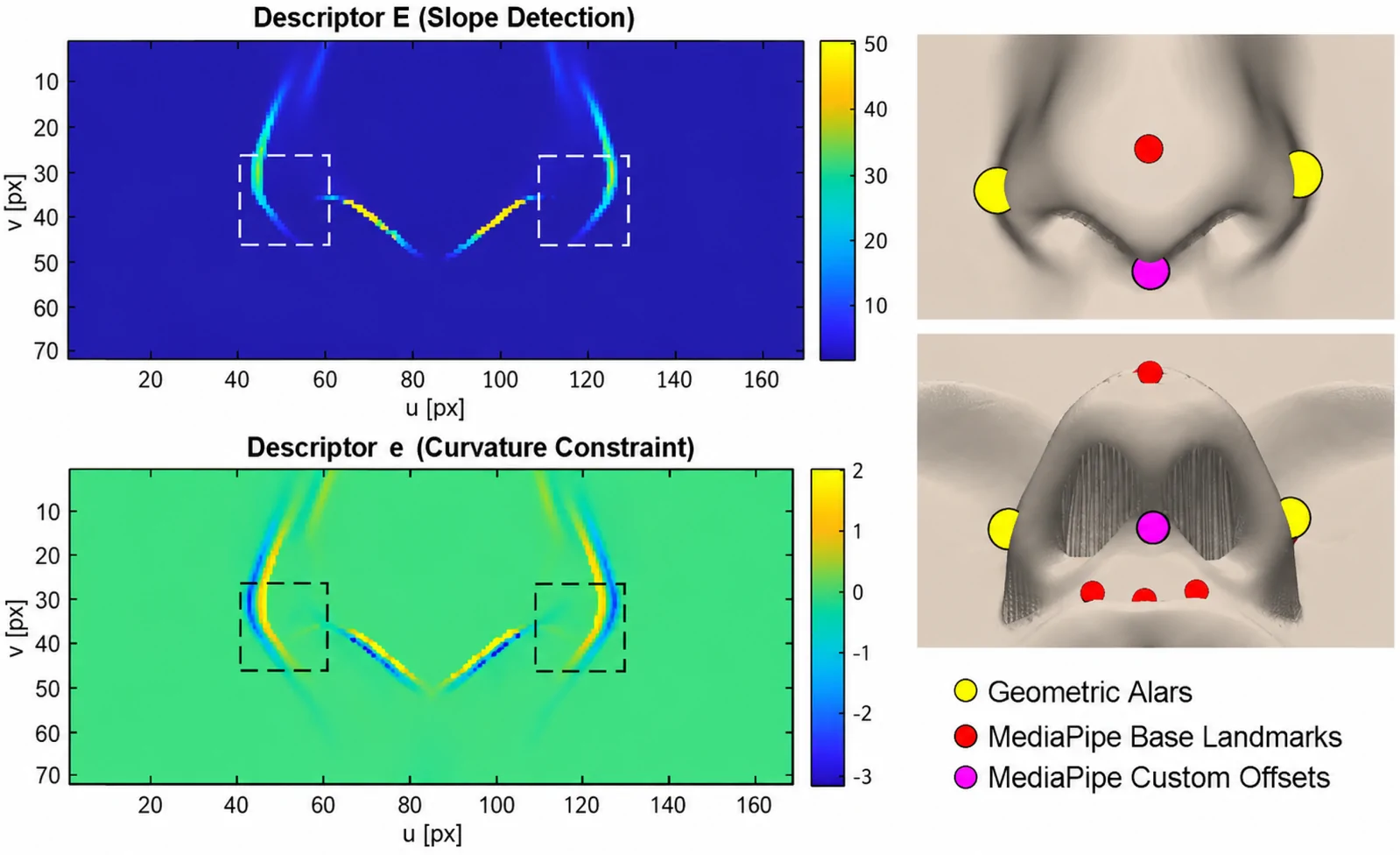
Alar landmarking: differential geometry descriptors *E* and *e* applied to the nasal surface (**left**), and the resulting alar landmarks (yellow points) projected onto the facial depth map (**right**).

**Figure 5:**
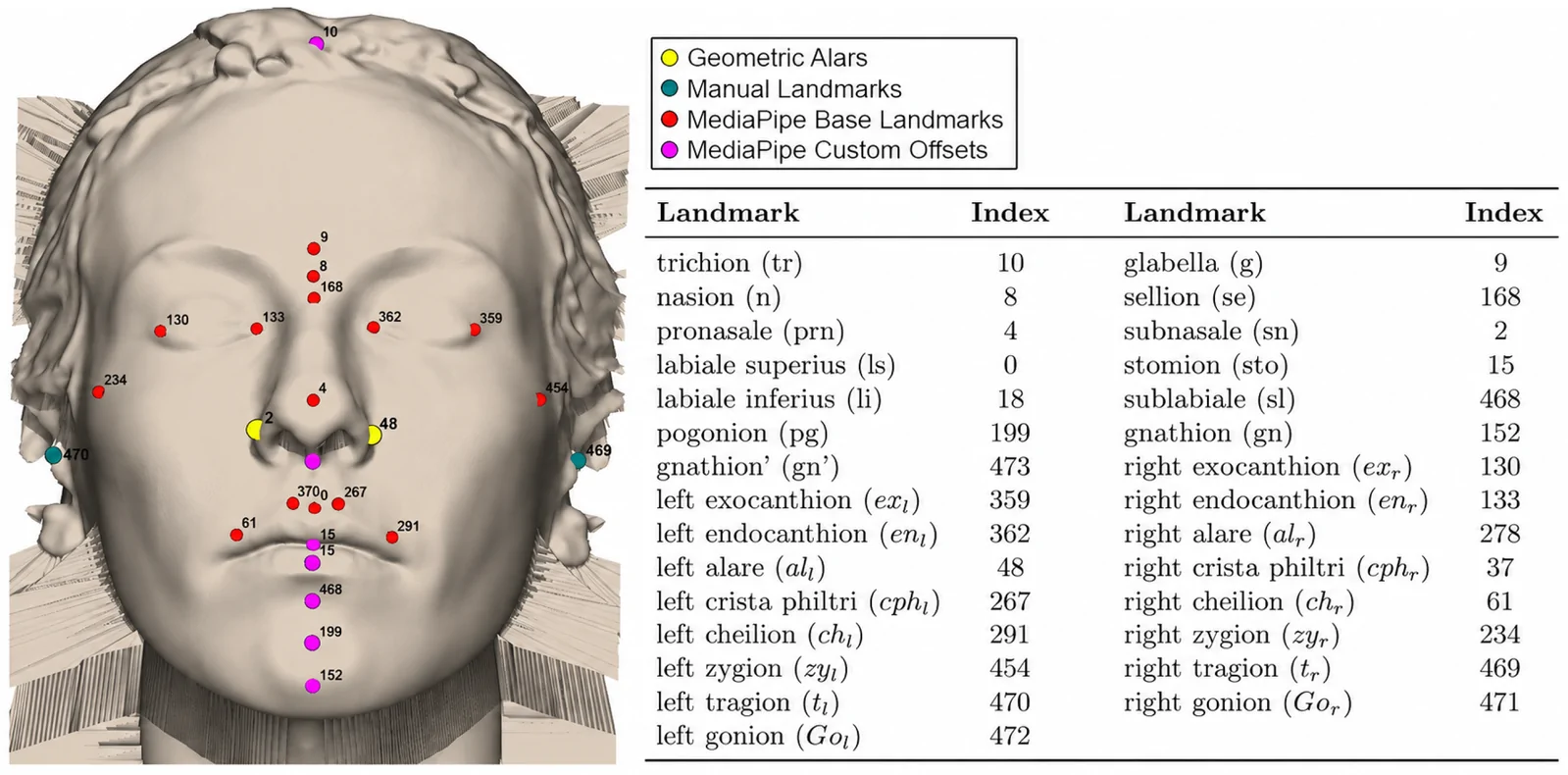
Final set of 29 landmarks visualised on the 3D depth map (female subject, ID 3).

For the two alar landmarks, the method was supplemented by an analysis based on differential geometry, using the coefficients of the fundamental forms *E* and *e* [15], which specifically highlight the nasal alar curvatures (Figure 4). Operationally, the analysis was restricted to two square regions of interest (ROIs), dynamically defined for each subject based on the pixel distance between the subnasale and pronasale landmarks; the system identifies the midpoint between the two landmarks, applies a lateral translation equal to 90% of their distance, and scales the side of each ROI to 65% of that distance, ensuring proportional coverage of the alar regions. Within these ROIs, the algorithm selects the point that maximises the gradient whilst simultaneously minimising the geometrical descriptor, thereby anchoring the points to the surface’s intrinsic geometric characteristics. Finally, landmarks that are not visible from the front or are associated with deep bony structures, such as the tragion, gonion, and gnathion, were positioned manually. Manual landmark placement was performed by a single experienced maxillofacial surgeon with detailed knowledge of the underlying bony anatomy and its relationship with the corresponding soft-tissue structures. This approach was adopted to standardize the manual positioning procedure and minimize inter-operator variability. Subsequently, these five manually placed landmarks were mapped into the coordinate system of the depth map (Figure 5).

### 2.5. Definition of Anatomical Reference System

**Figure 6:**
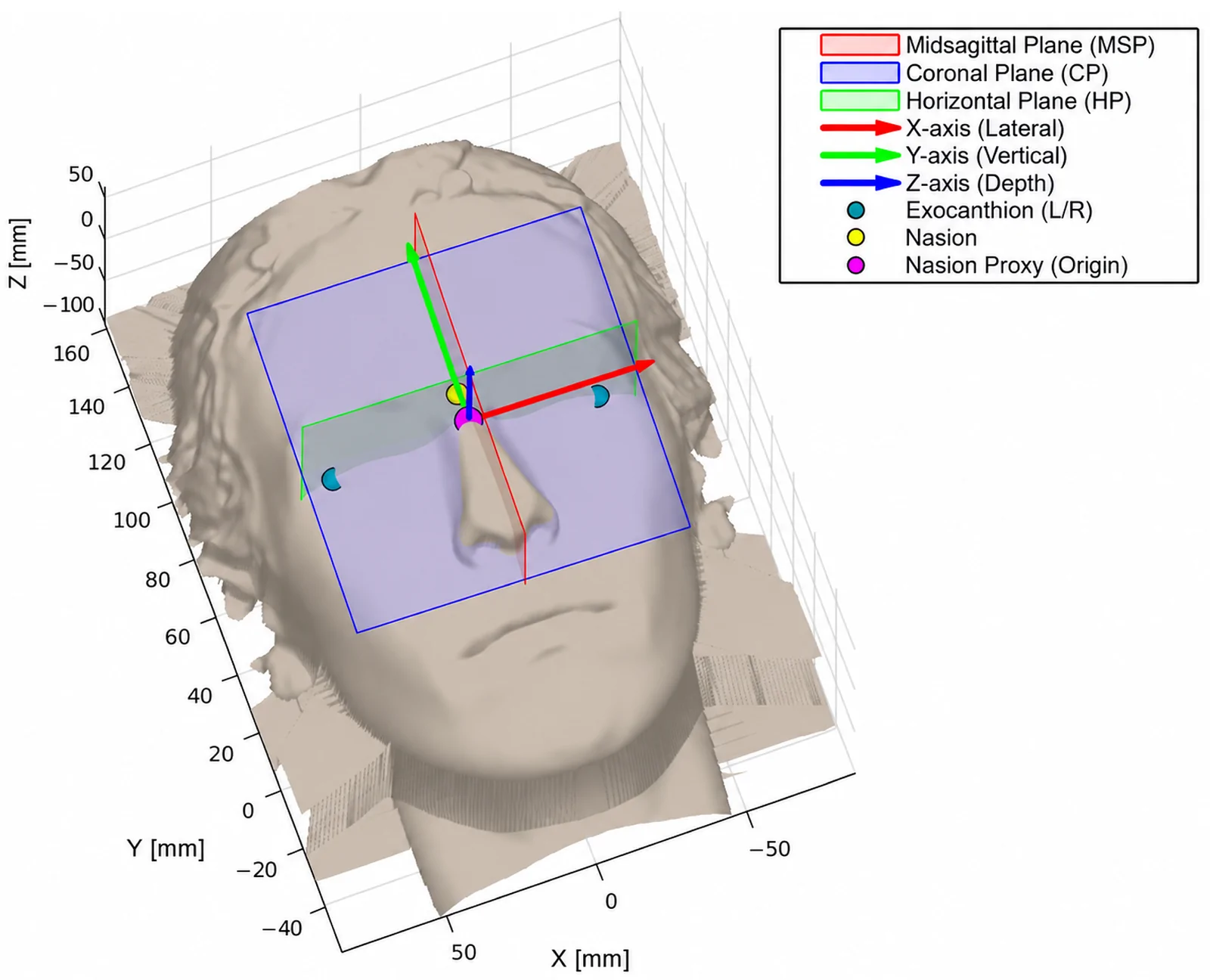
3D facial reference system with anatomical planes and axes (female subject, ID 3).

Once the landmarking is complete, a stable three-dimensional reference system (Figure 6), comparable across subjects, was constructed, which is essential both for calculating measurements and for assessing asymmetry. The horizontal plane (HP) was defined using the two exocanthion points and a midline reference point derived from the nasion, here referred to as the Nasion Proxy. This point retains the lateral coordinate of the nasion; its vertical coordinate is defined as the average of the two exocanthions, and its depth is obtained from its projection onto the depth map. The Nasion Proxy does not represent an anatomical landmark, but rather a geometrically constructed reference point used exclusively for the definition of the horizontal plane. The median sagittal plane (MSP) was constructed to pass through the Nasion Proxy and to be orthogonal to the lateral direction, whilst the coronal plane (CP) was defined as orthogonal to the previously defined planes. This approach follows the rationale of the Frankfort plane [16,17], a classical cephalometric reference for orienting the head in a standardized position. However, as the latter is defined using deep or posterior bony landmarks, it was approximated using stable landmarks that can be derived from 3D scans.

### 2.6. Database Validation and Symmetry Analysis

The initial database was subject to a screening process to ensure adequate morphological consistency for the purposes of this study. Before quantitative validation, all 3D facial scans underwent an individual qualitative quality-control assessment to verify the technical adequacy and completeness of the acquired facial surface. Each scan was visually inspected for acquisition- or reconstruction-related issues that could not be reliably corrected during subsequent processing. These included the presence of holes or missing surface data in anatomically relevant regions required for landmark identification and morphometric analysis. Only scans that passed this preliminary qualitative assessment proceeded to the quantitative validation phase. Quantitative validation was performed based on compliance with normotypical ranges and stringent asymmetry thresholds. Symmetry assessment was carried out using the Asymmetry Index (ASX) [18], applying different criteria depending on the type of landmark. For bilateral points, asymmetry was calculated as the three-dimensional difference between the two sides, allowing for the detection of discrepancies not only laterally but also in terms of height and depth:

(1) $$ASX_{bilateral}=\sqrt{\left(\right|x_{L}\left|-\right|x_{R}{\left|\right)}^{2}+\left(\right|y_{L}\left|-\right|y_{R}{\left|\right)}^{2}+\left(\right|z_{L}\left|-\right|z_{R}{\left|\right)}^{2}},$$

where x,y,z denote the spatial coordinates of the landmark in the local coordinate system, and the subscripts *L* and *R* identify the left and right sides, respectively. For medial landmarks, symmetry was assessed as lateral deviation from the median sagittal plane:

(2) $$ASX_{medial}=\left|x\right|,$$

where |x| represents the absolute distance of the landmark from the plane of symmetry. The acceptability thresholds for the ASX index were defined by integrating literature data [19] with clinical comparison, establishing more restrictive parameters for central points and more tolerant ones for peripheral regions, where slight millimetre-scale variability is physiological and has less impact on overall aesthetic harmony. The tolerance thresholds associated with each landmark of interest are reported in Table 2. Overall, the aim was not to identify perfectly symmetrical faces, but rather harmonious profiles that fell within the limits of clinical visual tolerance.

The database was then validated by comparing the measurements for which reliable reference values were available with the corresponding sex-specific normotypical ranges. The quantitative assessment was expressed as a percentage of conformity, defined as the ratio of the number of measurements falling within the respective normotypical range to the total number of measurements assessed for that category. Specifically, for each subject, separate percentages were calculated for linear measurements, angular measurements, and asymmetry indices, as well as an overall percentage based on all available measurements. This approach allowed for a concise quantification of each subject’s degree of agreement with the reference ranges, enabling the identification of cases characterised by greater overall morphological consistency and providing a robust database for subsequent modelling phases. Subjects with less than 80% compliance in any category underwent targeted review by the surgeons.

### 2.7. Unsupervised Clustering on the Mixed-Sex Population

To primarily assess the discriminatory power of the set of anthropometric features and analyse the overall structure of morphological variability, the analysis was extended to the entire population of the dataset, treated as a single group. In this context, all individuals were represented in a single data matrix, constructed from a vector collecting all anthropometric features and normalized using Z-scores [20,21], to ensure comparability between variables characterized by different units of measurement and scales.

The problem was formulated as an unsupervised clustering task in the multidimensional feature space, using the k-means algorithm with Euclidean distance. The algorithm was executed with a large number of random initialisations, in order to reduce dependence on the initial choice of centroids and ensure the stability of the final solution. The optimal number of clusters was determined using the Gap Statistic [22]. Candidate values of k ranging from 1 to 5 were considered, consistent with the sample size. In order to quantify the support associated with each value of k, the Gap Statistic values were normalized using the softmax function, yielding a distribution of relative weights, from which the confidence percentage associated with the optimal number of clusters was derived. This approach enables the evaluation of not only the most plausible solution, but also the level of evidence for the identified structure and the homogeneity of the dataset.

To explore the relative contribution of individual variables, a one-way analysis of variance (ANOVA) was conducted for each anthropometric measure. The Bonferroni-adjusted thresholds were used as descriptive indices to rank the most salient features driving cluster differentiation (p<0.00043, obtained as α/m, where α=0.05 and m=115).

### 2.8. Development of Normotypes

The construction of three-dimensional normotype models was approached as a problem of geometric mediation between unstructured facial surfaces, characterized by differences in both topology and vertex distribution. To ensure comparability between subjects and enable a consistent merging process, registration and homogenization of the geometric representations were required, as outlined in the methodology pipeline shown in Figure 1.

In the initial phase, the identified landmarks were transferred from the depth image space to the 3D mesh using a nearest-neighbour search, ensuring that each landmark was assigned a unique correspondence among the mesh vertices. Subsequently, a reference subject with a continuous surface and high vertex density, without gaps in key anthropometric regions, was identified for each sex to be used as a template. To isolate the shape component, a Generalised Procrustes Analysis (GPA) [23] was applied to the landmarks, which allowed the elimination of differences in translation, rotation and scale, aligning all subjects in a common space [2]. The topological registration was then obtained by imposing the template structure onto all the sample meshes using a mapping based on geometric proximity; thereby each subject was reconfigured with identical connectivity and the same number of vertices. The normotype was finally calculated as the arithmetic mean of the corresponding vertex coordinates, followed by a slight surface smoothing to correct any discretisation artefacts without altering the morphology. Then, linear distances, angular measurements and asymmetry indices were extracted from the average models after a surgeon had performed a completely manual landmarking procedure. These parameters were compared with the normotypical ranges defined in the literature to quantify to what extent the average facial shape adheres to literature-acknowledged morphological references.

## 3. Results and Discussion

This section presents and discusses the outcomes of the proposed methodology, exposed step by step in different subsections.

### 3.1. Literature-Based Landmark and Morphometric Measure Selection, and Identification of Normotypical Ranges

All literature research studies taken into consideration for the retrieval of normotypical data are reported in Table 3. The whole set of linear, angular and ratio measures with their reference numerical values found in the literature is reported in the Supplementary Materials. Interestingly, the analysis of the literature revealed a clear methodological complementarity; indeed, whilst pioneering studies based on direct anthropometry provide extremely detailed datasets, more recent digital studies offer larger sample sizes and greater reproducibility, albeit with a occasionally limited number of measurements.

**Figure 7:**
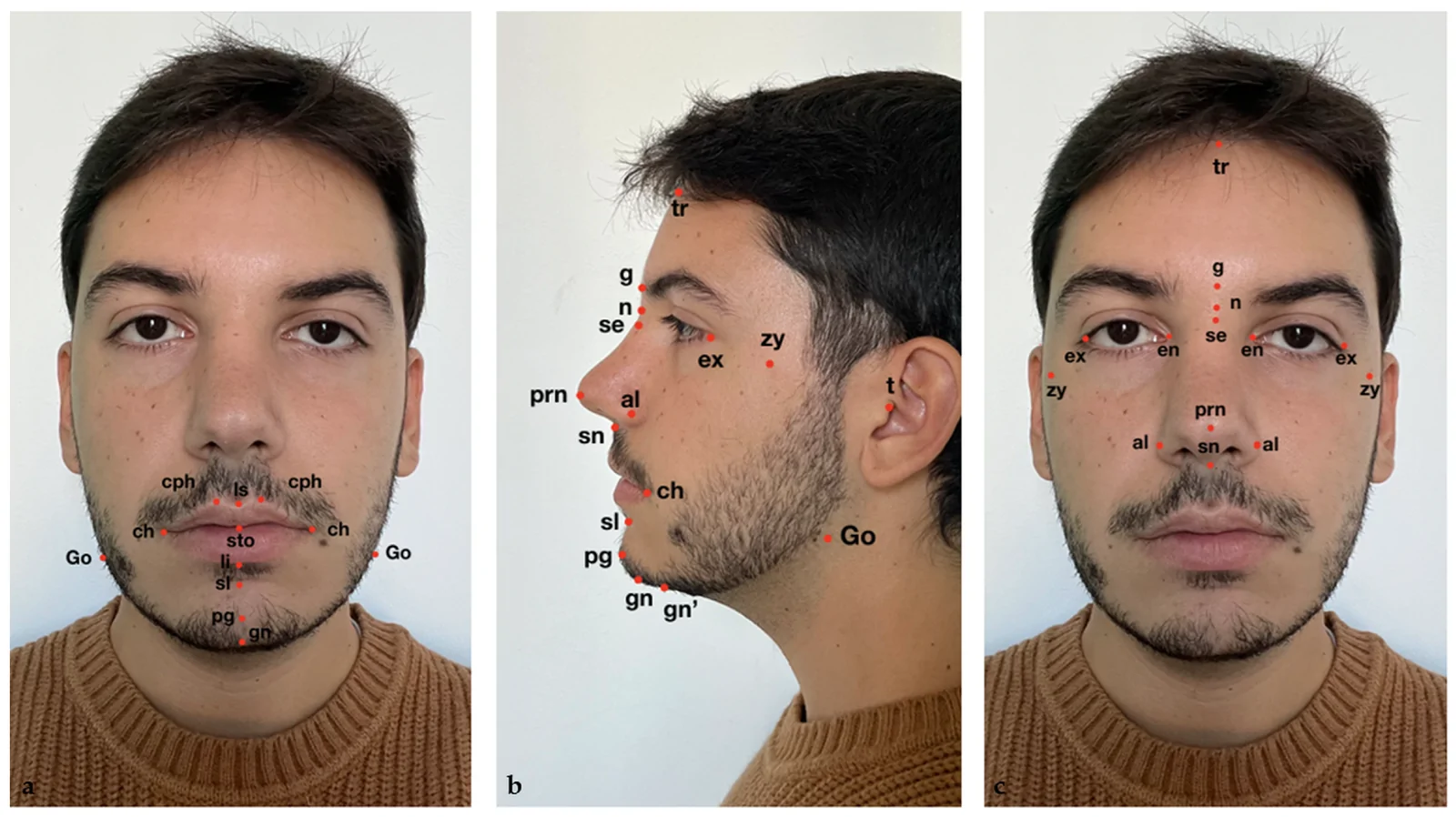
The complete set of 29 anatomical landmarks adopted for the computation of the selected anthropometric measurements. (**a**) Frontal view; lower third landmarks, (**b**) lateral view, (**c**) frontal view, mid and upper thirds landmarks.

The landmark selection process led to the definition of a final set of 29 anatomical landmarks, reported in Figure 7 and Table 4, serving as the basis for calculating morphometric measures. The selection of anthropometric measurements led to a set of 46 linear, 31 angular, and 18 ratio measurements (95 overall). Specifically, the linear measurements allow for the definition of the heights of the whole face and of individual facial segments, the transverse widths and the anteroposterior depths. These are complemented by angular measurements, which describe the inclination of structures, the convexity of the profile and the overall morphological harmony through the calculation of direct and projected angles and inclinations between segments. Finally, the assessment of overall geometric balance relies on facial proportions and indices, i.e., the vertical division of the face into three equal parts and the facial index calculated as the ratio of height to width. The corresponding sex-specific normotypical ranges are reported in Table 5, Table 6 and Table 7.

### 3.2. Database Validation and Symmetry Analysis

**Figure 8:**
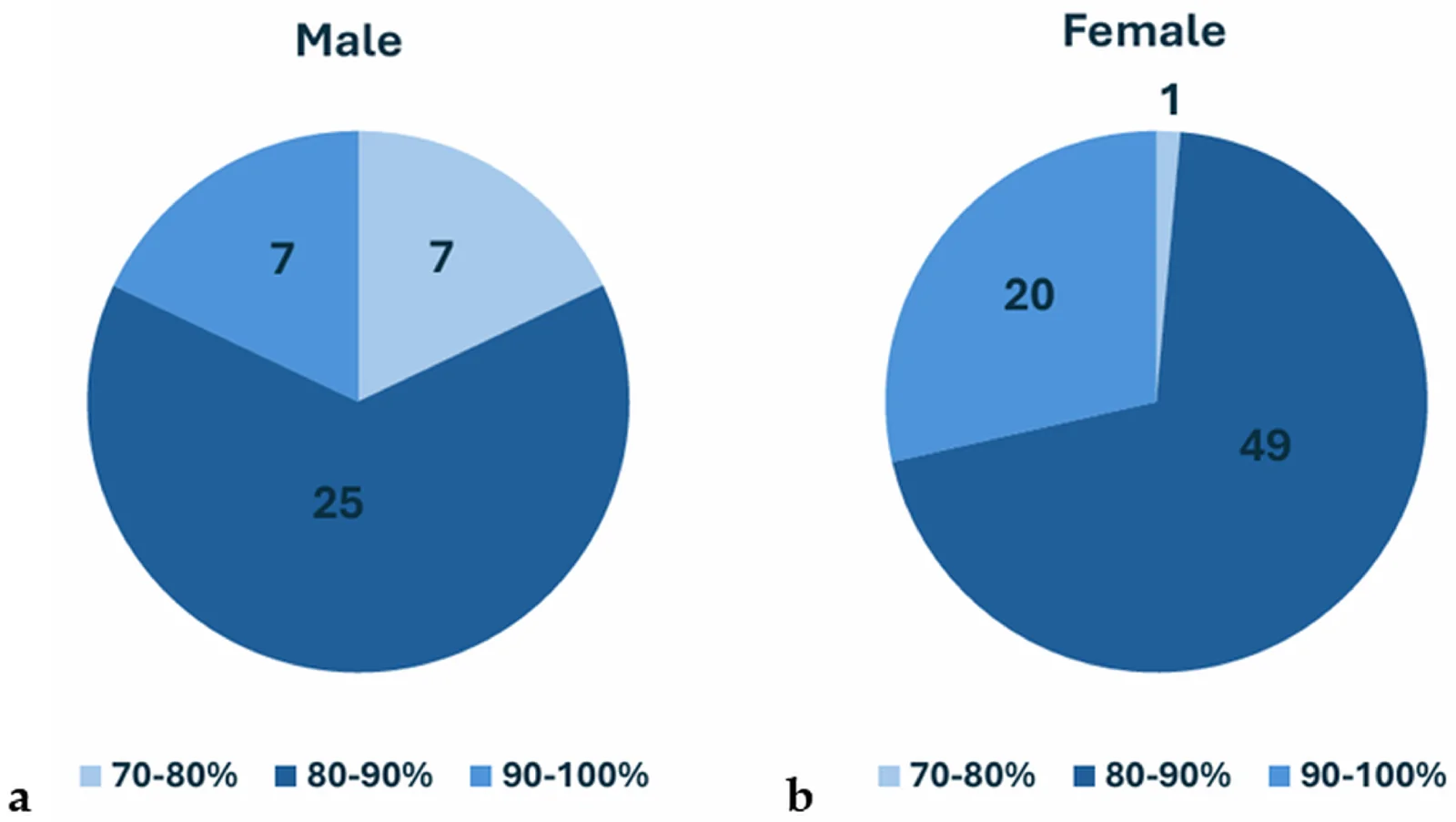
The pie charts report the number of individuals falling within the 70–80%, 80–90%, and 90–100% overall conformity intervals for the male (**a**) and female (**b**) groups.

Before quantitative validation, the initial database of 139 subjects underwent a qualitative screening of the 3D facial scans. Each scan was individually inspected to identify acquisition- or reconstruction-related issues that could not be adequately corrected during subsequent processing. This preliminary quality-control step led to the exclusion of 30 scans, resulting in a sample of 109 subjects entering the quantitative validation phase. On the resulting cohort of 109 subjects, quantitative validation was then performed by incorporating asymmetry indices into the morphometric analysis, increasing the measure set from 95 to 115. The validation of the database by comparison with normotypical ranges revealed a high degree of conformity in the selected sample. The overall percentages exceeded 75% for all subjects, with minimum values of 75% for females and 77% for males. The distribution of the overall percentages, shown in the pie charts (Figure 8), indicates that the majority of subjects fall within the highest compliance ranges (80–90% and 90–100%), confirming good agreement with the reference anthropometric parameters. Analysis by measurement category reveals greater stability for linear parameters, whereas angular measurements and asymmetry indices show slightly higher variability, attributable to both biological variability and greater sensitivity to landmark positioning. No further subjects were excluded during the quantitative phase: cases with compliance below 80% in specific categories were nevertheless compatible with clinical tolerance limits. The final database therefore consisted of 109 subjects (70 females, 39 males), constituting a morphologically consistent and robust sample for the subsequent modeling phases.

### 3.3. Clustering

**Figure 9:**
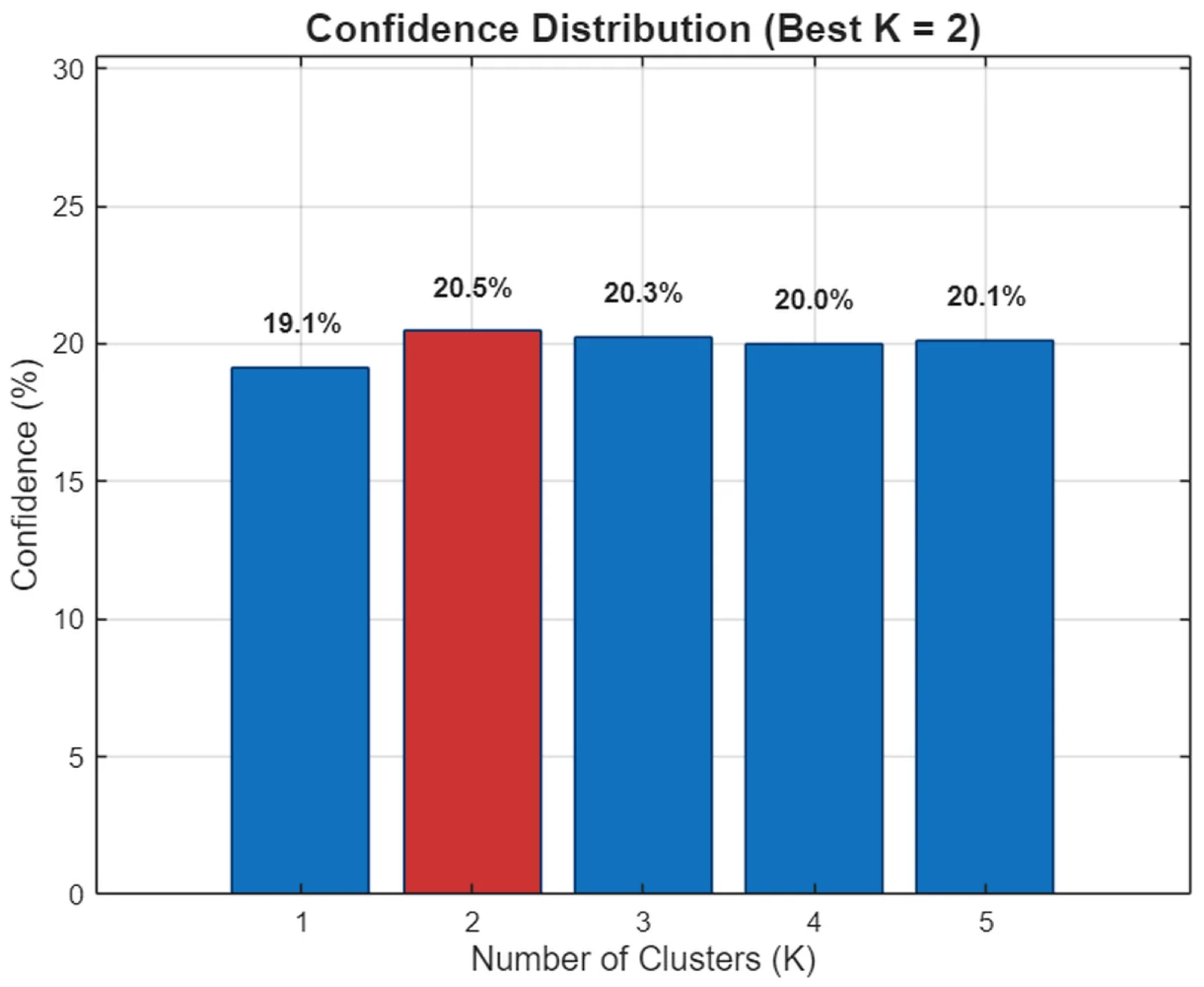
Distribution of confidence percentages associated with different values of the number of clusters k, calculated from the Gap Statistic via softmax normalization, on the entire dataset including male and female subjects. The column in red represents the selected k = 2 value, which produced a subdivision strongly consistent with the biological sex of the subjects.

The application of the Gap Statistic to the mixed population identified an optimal number of clusters of k = 2, with a maximum confidence of 20.5% (Figure 9).

**Figure 10:**
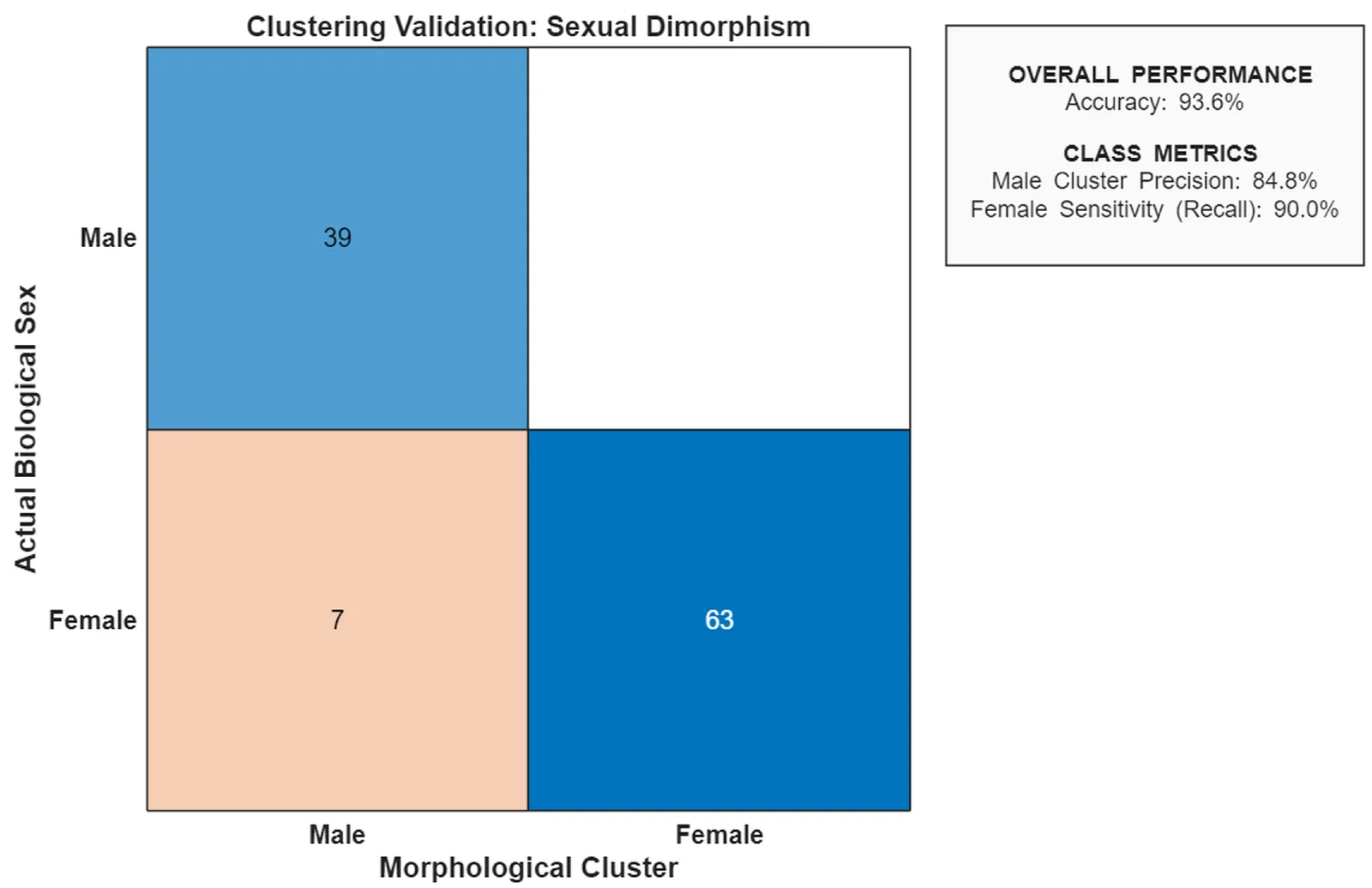
Confusion matrix of the classification induced by unsupervised clustering with k = 2, comparing morphological cluster membership with the actual biological sex of the subjects.

The relatively uniform distribution of confidence levels across the different values of k suggests that the morphological variability of the sample is not organised into clearly distinct groups, but rather distributed along a continuum. Despite this apparent continuity, clustering with k = 2 produced a subdivision strongly consistent with the biological sex of the subjects, achieving an overall accuracy of 93.6%. Specifically, one of the two clusters grouped 63 of the 70 women, whilst the second included all 39 men along with just 7 female subjects. This result, shown in the confusion matrix (Figure 10), is particularly significant as it demonstrates that sexual dimorphism emerges spontaneously from the data structure, without any prior information, highlighting its role as one of the main dimensions of morphological variability in the human face.

Notably, this level of accuracy was achieved using exclusively three-dimensional anthropometric measurements derived from landmarks—that is, synthetic features that are geometrically interpretable and clinically significant. Comparable results were achieved in previous research. Studies based on high-density 3D data [44], which adopt richer shape representations (e.g., via PCA on dense surfaces or local facial segmentation), report accuracies of up to 97%, whilst approaches based on continuous surface descriptors and classifiers such as Random Forest stand at around 93–94% [44]. In both cases, however, these methods rely on features that are difficult to interpret from a clinical perspective. In contrast, this study shows that a relatively compact set of three-dimensional anthropometric measurements is sufficient to effectively capture sexual dimorphism, achieving performance comparable to that obtained with more complex models. These findings suggest that discriminative information does not necessarily depend on the density of the representation, but on the ability of the features to meaningfully summarise facial morphology.

The ANOVA analysis further reinforced this finding, showing that 56 of the 115 measurements were statistically significant after Bonferroni correction. These variables, with their respective *p*-value values, are reported from the most discriminative (minor *p*-value) to the least discriminative (major *p*-value) in Table 8. The variables with the greatest discriminatory power predominantly involve the lower third of the face, in particular mandibular height and the height of the subnasal region, as well as measures of maxillary depth and transverse dimensions such as the zygomatic and bigonial widths. This result confirms that the observed separation is not only globally effective but is also supported by specific morphological patterns consistent with what is known about sexual dimorphism.

A further distinctive trait emerges when comparing this approach with other unsupervised studies [45]. In studies applying clustering techniques to 3D facial data, the analysis is often conducted separately by sex and aimed at identifying morphological categories within each group [45]. In our case, however, the separation between males and females emerges a posteriori within the mixed population, without any prior imposition. This reinforces the interpretation of biological sex as the dominant structural axis in the feature space, capable of spontaneously driving cluster formation.

### 3.4. Development of Normotypes

**Figure 11:**
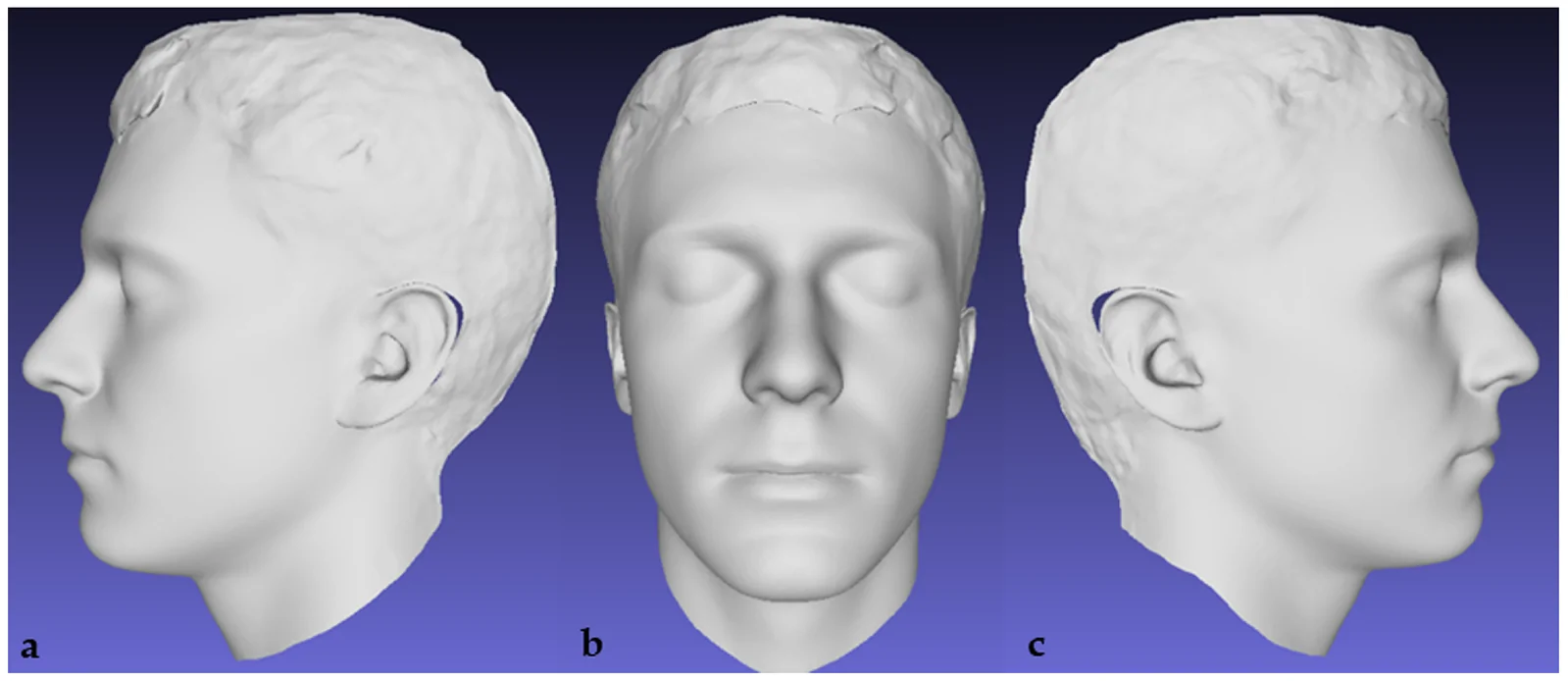
Final male normotype after post-processing and refinement in Mesh Lab. (**a**) Left lateral view; (**b**) frontal view; (**c**) right lateral view.

**Figure 12:**
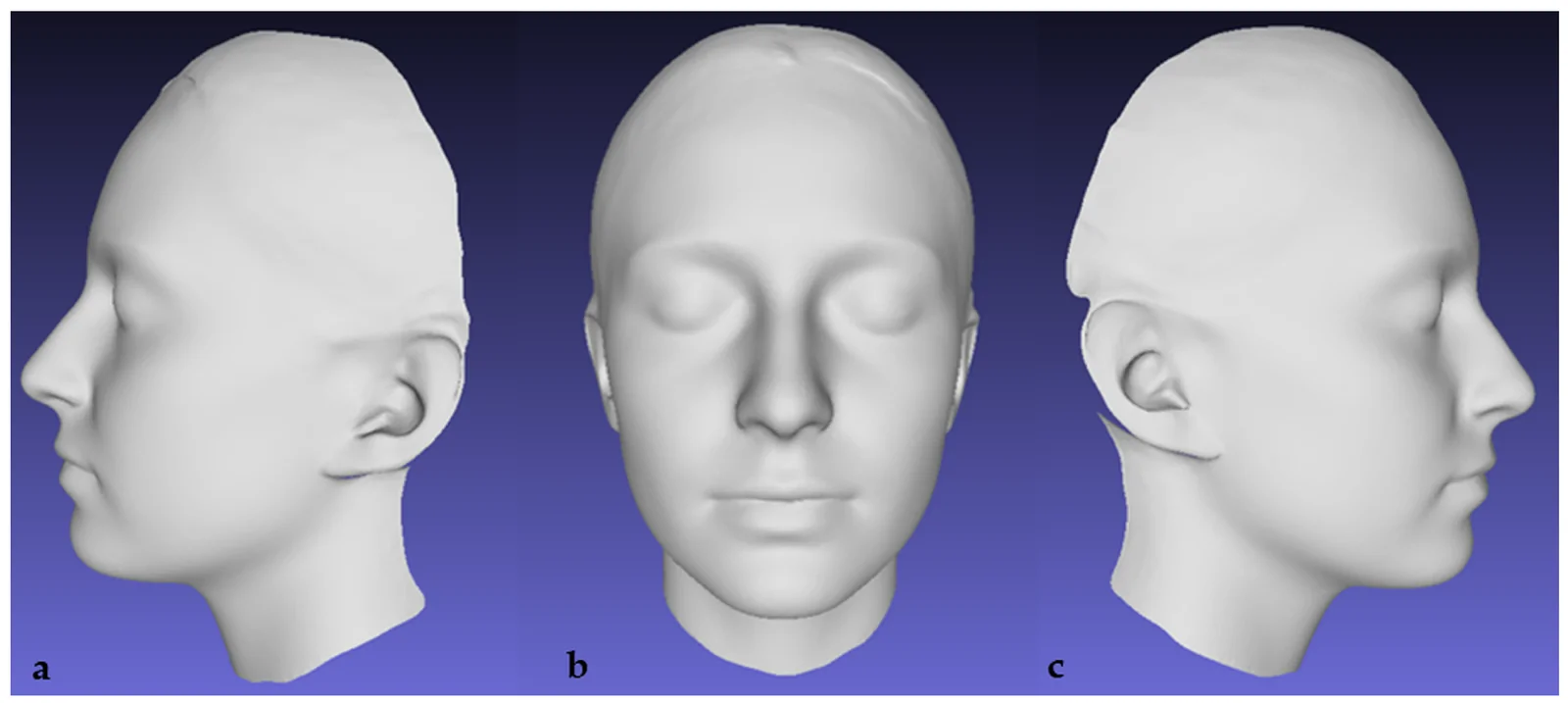
Final female normotype after post-processing and refinement in Mesh Lab. (**a**) Left lateral view; (**b**) frontal view; (**c**) right lateral view.

The resulting facial normotypes are shown in Figure 11 and Figure 12. The analysis of the anthropometric measurements showed a high level of agreement with the determined normotypical ranges. Indeed, the male normotype showed an overall conformity of 95%, whilst that of the female normotype was 91%, with high values across all measurement categories considered, as shown in Table 9.

Deviations from the normotypical ranges are limited and affect only a small number of parameters, with small variations that are consistent with the biological variability of the sample. These differences may be mainly attributable to the variability in manual landmark positioning, particularly in morphologically complex regions, and do not affect the overall representativeness of the normotype. Overall, these results reinforce the validity of the normotype model as a quantitative reference, highlighting its realism and ability to synthesize population morphology whilst maintaining consistency with established anthropometric parameters. This aspect is crucial from an application perspective, as it ensures that the normotype can be used as a reliable reference in clinical and surgical planning contexts.

The values of the 115 extracted anthropometric measurements for both normotypes are reported separately for proportions, linear measurements, angular measurements, and asymmetry indices in Table 10, Table 11, Table 12 and Table 13.

## 4. Preliminary Clinical Application

To illustrate the operational potential of the proposed pipeline, the normotype was applied to the planning of a representative clinical case. This section is intended solely as an illustrative example of the workflow and should not be interpreted as a clinical validation of the proposed approach, given the methodological limitations of this preliminary development study.

The patient was a 24-year-old male presenting with bimaxillary retrusion and skeletal and dental Class II malocclusion, who underwent bimaxillary advancement at the Maxillofacial Surgery Unit, AOU Città della Salute e della Scienza, Turin, Italy. Surgical planning was performed in house using Mimics Enlight CMF (Version 8.0, Materialise NV, Leuven, Belgium). Intermediate and final surgical splints were designed and manufactured in house using a Formlabs 3D printer (Formlabs Inc., Somerville, MA, USA) and were subsequently used to transfer the virtual surgical plan to the operating room.

**Figure 13:**
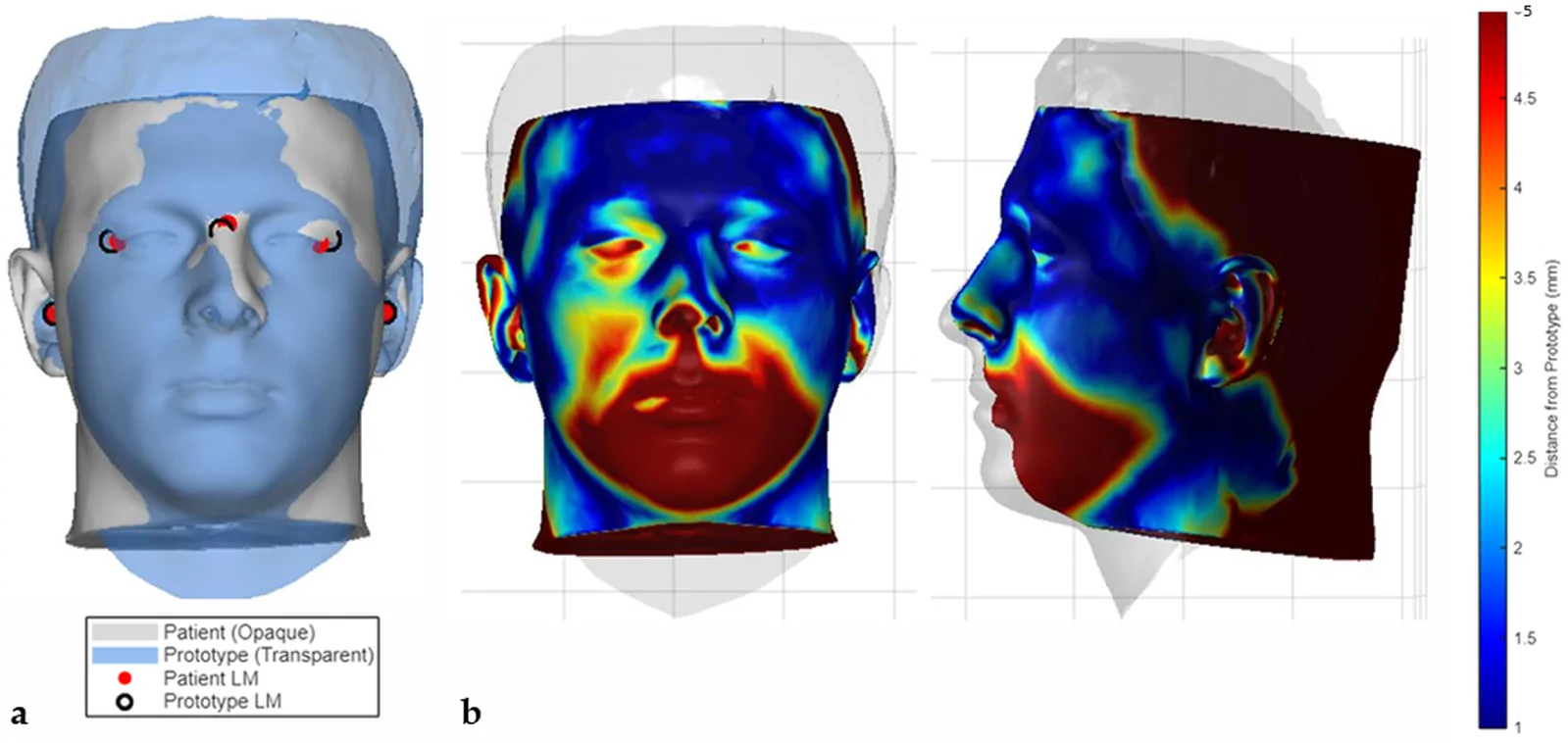
Example of a preliminary clinical application on a male patient with bimaxillary retrusion. (**a**) Rigid alignment based on the Procrustes method between the patient’s 3D scan, derived from a CT scan (shown in opaque grey), and the normotype (shown in transparent blue), obtained by identifying five stable craniofacial landmarks. (**b**) Heatmap of the discrepancies between the patient’s face and the normotype.

The normotype-based planning workflow began with the spatial alignment of the patient’s facial model to the corresponding male normotype using Generalized Procrustes Analysis. This was achieved by identifying five stable anatomical landmarks: the sellion, the bilateral tragion and the bilateral exocanthion (Figure 13). The choice of these specific landmarks is crucial, as, being situated in the upper third of the face or in peripheral areas, they are not affected by orthognathic osteotomy procedures.

Once the alignment had been established, the morphological comparison was carried out using a color heatmap, which allowed for the immediate visualization of the areas of greatest discrepancy, highlighting the chin and nasolabial region as the priority areas for intervention. The normotype served as a three-dimensional morphological reference to assist in the evaluation of facial asymmetries and soft-tissue deficiencies, supporting the definition of the desired postoperative facial morphology (Figure 13).

The operator was able to supplement their clinical assessments using an interactive graphical interface developed specifically to take direct measurements on the aligned models. Specifically, these measurements consist of calculating the spatial distances between pairs of homologous points, manually selected by the surgeon on the patient’s face and on the standard model according to the specific anatomical requirements of the case. This procedure made it possible to precisely quantify discrepancies in the three spatial directions, providing the surgeon with precise metric guidelines for correcting asymmetries and malformations. The surgical plan was subsequently completed in Mimics Enlight CMF according to the standard virtual surgical planning workflow.

## 5. Conclusions

In conclusion, this study demonstrates the feasibility of a methodological workflow for the development of quantitative 3D facial normotypes. The clinical case presented represents only a preliminary illustration of their potential application. The resulting models should therefore be considered preliminary and are not yet intended for use as validated references in surgical planning. Further studies in larger and independent populations are required to refine and validate these normotypes and to determine whether they can provide a reliable and clinically useful morphological reference for patient-specific surgical assessment and planning. The tool does not replace expert clinical assessment, but provides practical support for refining the surgical plan, whilst always maintaining due respect for the anatomical characteristics and specific functional expectations of each patient. A significant limitation of the present study is its intentional restriction to a Caucasian population, both for the selection of reference data and for the construction of the experimental dataset. This approach was adopted to ensure population homogeneity during the initial development of the normotype, given the substantial amount of data required to construct a reliable population-specific model. Consequently, the normotypes developed in the present study should be considered specific to the Caucasian population and are not directly applicable to individuals from other ethnic groups. The definition of the normotypical reference intervals between the absolute minimum and maximum bounds derived from the literature represents an outer envelope of physiological variability. While this approach prevents overly restrictive criteria across Caucasian cohorts, it yields broader intervals than weighted pooled estimations, which might reduce the specificity. Future developments should therefore extend the proposed methodology to other ethnic and geographical populations, enabling the development of population-specific normotypes and broader clinical applicability. Another limitation of the present study is that intra-observer reproducibility of manual landmark placement was not formally assessed. Although all manually identified landmarks were placed by the same experienced maxillofacial surgeon to standardize the procedure, a dedicated assessment of intra-observer variability would provide further methodological validation and should be considered in future studies.

## Figures and Tables

**Table 1:** Vertical correction factors (expressed in pixels) applied to the 2D coordinates extracted by MediaPipe.

Landmark (Index)	Male Offset [px]	Female Offset [px]	Translation
trichion (10)	−75	−82	Upwards
subnasale (2)	+6	+6	Downwards
stomion (15)	+3	+2	Downwards
labiale inferius (18)	−9	−12	Upwards
sublabiale (468)	+3	+3	Downwards
pogonion (199)	+7	+7	Downwards
gnathion (152)	+14	+14	Downwards

**Table 2:** ASX threshold values defined for the 20 analyzed parameters, divided by anatomical district and clinical tolerance.

Anatomical Region	Landmarks	Threshold (mm)
Medial (Midline)	tr,g,n,se,prn,sn,ls,sto,li,sl,pg,gn	2.0
Central Nasal and Labial	$al_{l}-al_{r},\;cph_{l}-cph_{r}$	2.0
Orbital (Ocular)	$ex_{l}-ex_{r},\;en_{l}-en_{r}$	3.0
External Labial	$ch_{l}-ch_{r}$	4.0
Peripheral (Latero-Posterior)	$zy_{l}-zy_{r},\;t_{l}-t_{r},\;Go_{l}-Go_{r}$	5.0

**Table 3:** Bibliographic sources used to collect the anthropometric reference data reported in the Supplementary tables.

First Author	Year	Bib. Ref.	First Author	Year	Bib. Ref.
Bozkir et al.	2004	[24]	Mengoa et al.	2024	[25]
Farkas	1981	[26]	Ozdemir et al.	2009	[27]
Farkas	1994	[5]	Porter	2004	[28]
Farkas et al.	2005	[6]	Sforza et al.	2009	[29]
Fernández-Riveiro et al.	2003	[30]	Sforza et al.	2011	[31]
Ferrario et al.	1996	[32]	Staka et al.	2013	[33]
Galantucci et al.	2016	[34]	Sutter and Turley	1998	[35]
Gu et al.	2011	[36]	Uysal et al.	2012	[37]
Gündüz Arslan et al.	2008	[38]	Uzun and Ozdemir	2014	[39]
Jagadish Chandra et al.	2012	[12]	Weinberg et al.	2016	[8]
Karaca et al.	2012	[40]	Wen et al.	2015	[41]
Malkoç et al.	2009	[42]	Winiarska et al.	2025	[43]

**Table 4:** List of the 29 soft tissue facial landmarks used in this study, including abbreviation, anatomical name, and standardized anatomical description. Bilateral landmarks are reported once for compactness.

Acronym	Name	Description
tr	trichion	Midline point located at the hairline on the sagittal plane.
g	glabella	Most prominent point on the soft tissue surface of the frontal region, located on the midline between the eyebrows.
n	nasion	Midline depression at the nasal root, corresponding to the intersection between the forehead and the nasal dorsum.
prn	pronasale	Most anterior point of the nasal tip.
sn	subnasale	Midline point at the base of the nose, at the junction between the nasal septum and the upper lip.
ls	labiale superius	Midline point on the vermilion border of the upper lip.
sto	stomion	Midline point of contact between upper and lower lip in resting position.
li	labiale inferius	Midline point on the vermilion border of the lower lip.
sl	sublabiale	Point of maximum concavity of the soft tissue profile between lower lip and chin.
pg	pogonion	Most anterior point of the soft tissue chin profile.
gn	gnathion	Most inferior and anterior point of the soft tissue chin contour on the midline.
se	sellion	Point of maximum concavity of the nasal dorsum between glabella and pronasale.
cph	crista philtri	Point of maximum elevation of the philtral ridge.
ch	cheilion	Labial commissure, at the intersection between upper and lower lip.
en	endocanthion	Point located at the inner corner of the palpebral fissure.
ex	exocanthion	Point located at the outer corner of the palpebral fissure.
al	alare	Most lateral point on the contour of the nasal ala.
t	tragion	Point located at the superior notch of the auricular tragus.
Go	gonion	Point located at the mandibular angle, defined by the intersection of the posterior border of the ramus and the inferior border of the mandible.
zy	zygion	Most lateral point of the zygomatic arch on the soft tissue surface.
gn’	gnathion’	Point located posterior to gnathion on the midline, tangent to the chin profile.

**Table 5:** Normotypical range of selected linear measurements for male and female subjects. Values represent the overall minimum and maximum limits derived from the literature synthesis (expressed in mm).

		Male		Female	
Measurement	Definition	Min	Max	Min	Max
Lower half of the cranial height (right/left)	en–gn	61	130	81	120
Physiognomical height of the face	tr–gn	149	223	137	211
Height of forehead II	tr–n	40	97	40	94
Height of the nose	n–sn	37	74	39	84
Height of the chin	sl–gn	19	39	17	32
Distance between glabella and subnasale	g–sn	40	88	36	80
Height of the upper lip	sn–sto	14	31	14	27
Lower lip height	sto–sl	13	27	8	27
Height of the lower face	sn–gn	47	91	45	81
Height of the mandible	sto–gn	35	68	28	56
Length of the nasal bridge	n–prn	26	60	29	54
Anterior lower facial height	sn–pg	42	58	40	56
Vermilion height of the upper lip	ls–sto	3	12	3	7
Vermilion height of the lower lip	li–sto	4	14	5	15
Length of the eye fissure (right/left)	ex–en	23	50	22	55
Width of the nose	al_*r*_–al_*l*_	26	58	23	54
Width of the mouth	ch_*r*_–ch_*l*_	36	65	35	65
Biocular width	ex_*r*_–ex_*l*_	76	111	72	112
Width of the philtrum	cph_*r*_–cph_*l*_	8	18	7	16
Endocanthion–facial midline distance (right/left)	en–se	21	30	19	25
Width of the face	zy_*r*_–zy_*l*_	97	162	94	155
Width of the mandible	Go_*r*_–Go_*l*_	80	142	77	143
Intercanthal width	en_*r*_–en_*l*_	20	41	21	39
Skull-base width	t_*r*_–t_*l*_	125	161	112	153
Labio-aural distance (right/left)	ch–t	104	125	94	113
Nasal tip protrusion	sn–prn	13	28	13	26
Depth of the upper third of the face (right/left)	n–t	110	138	100	129
Depth in the maxillary region (right/left)	sn–t	111	143	99	135
Depth in the mandibular region (right/left)	gn–t	125	170	120	151
Depth of the lower jaw (right/left)	gn–Go	76	97	74	89
Depth of the supraorbital rim (right/left)	t–g	118	141	108	130
Orbito-glabellar distance (right/left)	ex–g	46	58	43	55
Orbitotragial distance (right/left)	ex–t	78	92	71	86
Orbitogonial distance (right/left)	ex–Go	95	113	84	102

**Table 6:** Normotypical range of selected angular measurements for male and female subjects. Values represent the overall minimum and maximum limits derived from the literature synthesis (expressed in degrees).

		Male		Female	
Measurement	Definition	Min	Max	Min	Max
Exocanthion–nasion angle	ex_*l*_–n–ex_*r*_	95	125	94	138
Tragion–nasion–tragion angle	t_*l*_–n–t_*r*_	62	82	60	79
Tragion–pronasale–tragion angle	t_*l*_–prn–t_*r*_	54	73	55	70
Tragion–pogonion–tragion angle	t_*l*_–pg–t_*r*_	52	71	53	70
Inclination of the upper face profile	g–sn/y-plane	−4	9	−5	8
Inclination of the Leiber line	g–ls/y-plane	−8	10	−3	7
Inclination of the lower face profile	sn–pg/y-plane	−21	0	−22	−4
Inclination of the mandible	li–pg/y-plane	−28	−2	−33	−6
Inclination of the chin	sl–pg/y-plane	−4	38	−10	28
Inclination of the general profile line	g–pg/y-plane	−10	4	−10	2
Inclination of the nasal bridge	n–prn/y-plane	23	38	22	38
Inclination of the upper lip	ls–sn/y-plane	−16	19	−14	16
Inclination of the lower lip	li–sl/y-plane	−82	−11	−77	−20
Deviation of the nasal bridge	n–prn/z-plane	0	10	1	6
Inclination of the labial fissure	ch_*r*_–ch_*l*_/x-plane	−1	5	–	–
Nasofrontal angle	g–n–prn	116	162	113	158
Nasomental angle	n–prn–pg	123	145	123	144
Labiomental angle	li–sl–pg	72	155	93	163
Angle of facial convexity	n–sn–pg	157	179	148	177
Angle of total facial convexity	g–sn–pg	160	188	151	180
Variant of nasolabial angle	prn–sn–ls	–	–	102	150
Nasofacial angle	g–prn–pg	127	155	128	159
Nasal convexity angle	sl–n–sn	–	–	4	16
Nasal tip angle	n–prn–sn	62	107	58	105
Midface convexity angle (right/left)	n–t–sn	24	37	23	36
Mentocervical angle	sl–pg/gn–gn’	63	94	65	103

**Table 7:** Selected facial proportions and indices, and related reference values.

Category	Parameter	Definition
Vertical proportions	Vertical subdivision of the face	tr–n:n–sn:sn–gn = 1:1:1
Horizontal proportions	Horizontal facial subdivision	zy–ex:ex–en:en–en:en–ex:ex–zy = 1:1:1:1:1
Labial proportions	Lip–mandible ratio	sn–sto:sto–gn = 1:2
Facial index	Facial index	(n–gn)/(zy–zy)
Facial index	Upper facial index	(n–sto)/(zy–zy)
Facial index	Mouth–facial width index	(ch–ch)/(zy–zy)
Facial index	Lower facial height index	(sn–gn)/(n–gn)

**Table 8:** Anthropometric measurements significantly different between the two clusters, identified by one-way ANOVA with Bonferroni correction.

	Measure	*p*-Value
1	Height of lower face (sn−gn)	$4.74\times 10^{-30}$
2	Height of mandible (sto−gn)	$8.29\times 10^{-26}$
3	Height of chin (sl−gn)	$2.95\times 10^{-25}$
4	Depth in maxillary region $\left(sn-t_{r}\right)$	$3.82\times 10^{-25}$
5	Depth in maxillary region $\left(sn-t_{l}\right)$	$1.12\times 10^{-23}$
6	Depth of upper third of face $\left(n-t_{l}\right)$	$7.14\times 10^{-23}$
7	Depth of supraorbital rim $\left(t_{l}-g\right)$	$7.18\times 10^{-23}$
8	Lower half of cranial height $\left(en_{r}-gn\right)$	$1.10\times 10^{-22}$
9	Lower half of cranial height $\left(en_{l}-gn\right)$	$2.13\times 10^{-22}$
10	Anterior lower facial height (sn−pg)	$2.40\times 10^{-22}$
11	Depth of upper third of face $\left(n-t_{r}\right)$	$4.51\times 10^{-22}$
12	Depth of supraorbital rim $\left(t_{r}-g\right)$	$1.22\times 10^{-21}$
13	Depth of lower jaw $\left(gn-Go_{r}\right)$	$2.34\times 10^{-21}$
14	Depth in mandibular region $\left(gn-t_{r}\right)$	$6.98\times 10^{-21}$
15	Labio-aural distance $\left(ch_{r}-t_{r}\right)$	$3.06\times 10^{-20}$
16	Depth of lower jaw $\left(gn-Go_{l}\right)$	$4.92\times 10^{-20}$
17	Depth in mandibular region $\left(gn-t_{l}\right)$	$7.63\times 10^{-20}$
18	Skull-base width $\left(t_{r}-t_{l}\right)$	$1.30\times 10^{-19}$
19	Width of mandible $\left(Go_{r}-Go_{l}\right)$	$2.97\times 10^{-19}$
20	Width of face $\left(zy_{r}-zy_{l}\right)$	$2.82\times 10^{-18}$
21	Labio-aural distance $\left(ch_{l}-t_{l}\right)$	$7.02\times 10^{-18}$
22	Lower facial height index $\left((sn-gn)/(n-gn)\right)$	$9.53\times 10^{-18}$
23	Vertical subdivision $\left((n-sn)/(sn-gn)\right)$	$1.15\times 10^{-17}$
24	Orbitogonial distance $\left(ex_{r}-Go_{r}\right)$	$4.09\times 10^{-17}$
25	Endocanthion–facial midline $\left(en_{l}-se\right)$	$6.18\times 10^{-17}$
26	Physiognomical height of face (tr−gn)	$7.57\times 10^{-17}$
27	Endocanthion–facial midline $\left(en_{r}-se\right)$	$2.32\times 10^{-15}$
28	Orbitogonial distance $\left(ex_{l}-Go_{l}\right)$	$3.91\times 10^{-15}$
29	Orbitotragial distance $\left(ex_{r}-t_{r}\right)$	$3.11\times 10^{-13}$
30	Orbitotragial distance $\left(ex_{l}-t_{l}\right)$	$1.07\times 10^{-12}$
31	Height of upper lip (sn−sto)	$1.88\times 10^{-12}$
32	Width of nose $\left(al_{r}-al_{l}\right)$	$2.30\times 10^{-12}$
33	Lower lip height (sto−sl)	$9.24\times 10^{-12}$
34	Height of forehead II (tr−n)	$1.23\times 10^{-10}$
35	Biocular width $\left(ex_{r}-ex_{l}\right)$	$1.83\times 10^{-9}$
36	Inclination of nasal bridge $\left(n-prn/y^{\prime }\right)$	$2.05\times 10^{-9}$
37	Orbito-glabellar distance $\left(ex_{l}-g\right)$	$2.62\times 10^{-8}$
38	Vertical subdivision $\left((tr-n)/(n-sn)\right)$	$5.86\times 10^{-8}$
39	Orbito-glabellar distance $\left(ex_{r}-g\right)$	$1.05\times 10^{-7}$
40	Width of philtrum $\left(cph_{r}-cph_{l}\right)$	$2.37\times 10^{-7}$
41	Vermilion height lower lip (li−sto)	$5.38\times 10^{-7}$
42	Intercanthal width $\left(en_{r}-en_{l}\right)$	$5.43\times 10^{-7}$
43	Midface convexity angle $\left(n-t_{r}-sn\right)$	$1.38\times 10^{-6}$
44	Length of eye fissure $\left(en_{r}-ex_{r}\right)$	$3.86\times 10^{-6}$
45	Vertical subdivision $\left((tr-n)/(sn-gn)\right)$	$4.42\times 10^{-6}$
46	Length of eye fissure $\left(en_{l}-ex_{l}\right)$	$1.10\times 10^{-5}$
47	Width of mouth $\left(ch_{r}-ch_{l}\right)$	$2.23\times 10^{-5}$
48	Inclination of upper face $\left(g-sn/y^{\prime }\right)$	$2.43\times 10^{-5}$
49	Horizontal eye angle $\left(ex_{l}-en_{l}\right)$	$4.00\times 10^{-5}$
50	Midface convexity angle $\left(n-t_{l}-sn\right)$	$4.13\times 10^{-5}$
51	Length of nasal bridge (n−prn)	$5.97\times 10^{-5}$
52	Horizontal subdivision $\left(\left(en_{l}-ex_{l}\right)/\left(ex_{l}-zy_{l}\right)\right)$	$6.60\times 10^{-5}$
53	Inclination of general profile $\left(g-pg/y^{\prime }\right)$	$6.68\times 10^{-5}$
54	Horizontal subdivision $\left(\left(zy_{r}-ex_{r}\right)/\left(en_{l}-ex_{l}\right)\right)$	$8.84\times 10^{-5}$
55	Horizontal subdivision $\left(\left(zy_{r}-ex_{r}\right)/\left(ex_{r}-en_{r}\right)\right)$	$2.47\times 10^{-4}$
56	Horizontal subdivision $\left(\left(zy_{r}-ex_{r}\right)/\left(en_{r}-en_{l}\right)\right)$	$4.13\times 10^{-4}$

**Table 9:** Conformity with normotypical ranges (%) by measurement category for the male and female normotypes.

Categories	Male (%)	Female (%)
Linear measurements	91	89
Angular measurements	96	96
Asymmetry indices (ASX)	100	90
Overall compliance	95	91

**Table 10:** Proportional anthropometric measurements extracted for the male and female normotypes.

Measurement	Male	Female
Facial index (*n*–gn/zy–zy)	0.9	0.8
Upper facial index (*n*–sto/zy–zy)	0.6	0.6
Mouth–facial width index (ch–ch/zy–zy)	0.3	0.3
Lower facial height index (sn–gn/n–gn)	0.5	0.5
Vertical subdivision (tr–n/n–sn)	0.8	1.0
Vertical subdivision (tr–n/sn–gn)	0.7	1.1
Vertical subdivision (*n*–sn/sn–gn)	0.9	1.1
Lip–mandible ratio (sn–sto/sto–gn)	0.5	0.5
Horizontal subdivision ($zy_{r}$–$ex_{r}/ex_{r}$–$en_{r}$)	0.6	0.5
Horizontal subdivision ($zy_{r}$–$ex_{r}/en$–en)	0.5	0.6
Horizontal subdivision ($zy_{r}$–$ex_{r}/en_{l}$–$ex_{l}$)	0.6	0.5
Horizontal subdivision ($zy_{r}$–$ex_{r}/ex_{l}$–$zy_{l}$)	1.0	1.0
Horizontal subdivision ($ex_{r}$–$en_{r}/en$–en)	0.8	1.2
Horizontal subdivision ($ex_{r}$–$en_{r}/en_{l}$–$ex_{l}$)	1.0	1.0
Horizontal subdivision ($ex_{r}$–$en_{r}/ex_{l}$–$zy_{l}$)	1.5	2.0
Horizontal subdivision (en–$en/en_{l}$–$ex_{l}$)	1.2	0.8
Horizontal subdivision (en–$en/ex_{l}$–$zy_{l}$)	1.8	1.7
Horizontal subdivision ($en_{l}$–$ex_{l}/ex_{l}$–$zy_{l}$)	1.5	2.0

**Table 11:** Linear anthropometric measurements extracted for the male and female normotypes. Blue values indicate measurements that fall outside the established normotype ranges.

Measurement	Male	Female
Physiognomical height of face (tr–gn)	181.9	176.4
Width of face ($zy_{r}$–$zy_{l}$)	140.1	135.2
Skull-base width ($t_{r}$–$t_{l}$)	150.3	140.8
Width of mandible ($Go_{r}$–$Go_{l}$)	127.3	119.2
Depth of supraorbital rim ($t_{r}$–*g*)	132.4	125.0
Depth of supraorbital rim ($t_{l}$–*g*)	133.6	126.5
Depth of upper third of face (*n*–$t_{r}$)	130.0	121.3
Depth of upper third of face (*n*–$t_{l}$)	129.8	122.5
Orbitotragial distance ($ex_{r}$–$t_{r}$)	80.0	73.9
Orbitotragial distance ($ex_{l}$–$t_{l}$)	80.3	78.5
Depth in maxillary region (sn–$t_{r}$)	131.0	121.2
Depth in maxillary region (sn–$t_{l}$)	131.7	122.6
Orbitogonial distance ($ex_{r}$–$Go_{r}$)	97.4	81.7
Orbitogonial distance ($ex_{l}$–$Go_{l}$)	97.6	84.0
Labio-aural distance ($ch_{r}$–$t_{r}$)	112.9	103.0
Labio-aural distance ($ch_{l}$–$t_{l}$)	112.9	106.4
Depth in mandibular region (gn–$t_{r}$)	145.9	133.2
Depth in mandibular region (gn–$t_{l}$)	145.5	134.8
Height of forehead II (tr–*n*)	49.8	61.7
Orbito-glabellar distance ($ex_{r}$–*g*)	60.5	60.3
Orbito-glabellar distance ($ex_{l}$–*g*)	61.5	58.1
Endocanthion–facial midline ($en_{r}$–se)	24.8	20.4
Endocanthion–facial midline ($en_{l}$–se)	24.4	19.7
Intercanthal width ($en_{r}$–$en_{l}$)	37.0	28.9
Biocular width ($ex_{r}$–$ex_{l}$)	99.5	99.3
Length of eye fissure ($en_{r}$–$ex_{r}$)	32.9	37.4
Length of eye fissure ($en_{l}$–$ex_{l}$)	32.8	36.1
Length of nasal bridge (*n*–prn)	56.3	52.1
Nasal tip protrusion (sn–prn)	19.8	20.8
Height of nose (*n*–sn)	62.9	60.7
Distance glabella–subnasale (*g*–sn)	71.2	70.6
Width of nose ($al_{r}$–$al_{l}$)	34.9	30.6
Width of mouth ($ch_{r}$–$ch_{l}$)	45.6	43.1
Width of philtrum ($cph_{r}$–$cph_{l}$)	13.5	12.3
Height of upper lip (sn–sto)	23.1	16.8
Vermilion height upper lip (ls–sto)	7.0	7.0
Vermilion height lower lip (li–sto)	7.7	9.1
Lower lip height (sto–sl)	19.8	15.6
Height of mandible (sto–gn)	46.1	37.2
Anterior lower facial height (sn–pg)	57.5	44.8
Height of lower face (sn–gn)	69.2	54.0
Lower half of cranial height ($en_{r}$–gn)	118.7	103.3
Lower half of cranial height ($en_{l}$–gn)	118.3	102.9
Height of chin (sl–gn)	26.3	21.6
Depth of lower jaw (gn–$Go_{r}$)	111.3	95.3
Depth of lower jaw (gn–$Go_{l}$)	111.6	96.5

**Table 12:** Angular anthropometric measurements extracted for the male and female normotypes. Blue values indicate measurements that fall outside the established normotype ranges.

Measurement	Male	Female
Exocanthion–nasion angle ($ex_{r}$–*n*–$ex_{l}$)	117.5	124.3
Tragion–nasion–tragion angle ($t_{r}$–*n*–$t_{l}$)	70.7	70.6
Tragion–pronasale–tragion angle ($t_{r}$–prn–$t_{l}$)	63.5	63.7
Tragion–pogonion–tragion angle ($t_{r}$–pg–$t_{l}$)	62.8	64.8
Nasal tip angle (*n*–prn–sn)	103.1	105.0
Midface convexity angle (*n*–$t_{r}$–sn)	34.7	36.0
Midface convexity angle (*n*–$t_{l}$–sn)	34.7	34.7
Nasofacial angle (*g*–prn–pg)	137.8	137.2
Total facial convexity (*g*–sn–pg)	163.4	165.7
Variant of nasolabial angle (prn–sn–ls)	133.0	124.9
Angle of facial convexity (*n*–sn–pg)	162.3	164.6
Nasomental angle (*n*–prn–pg)	134.4	133.0
Nasofrontal angle (*g*–*n*–prn)	153.0	152.9
Labiomental angle (li–sl–pg)	145.4	133.7
Nasal convexity angle (sl–*n*–sn)	9.1	7.1
Mentocervical angle (sl–pg/gn–$gn^{\prime }$)	107.3	105.8
Inclination of chin (sl–$pg/y^{\prime }$)	6.3	3.7
Inclination of general profile (*g*–$pg/y^{\prime }$)	1.3	−0.8
Inclination of nasal bridge (*n*–$prn/y^{\prime }$)	27.4	25.0
Inclination of upper lip (sn–$ls/y^{\prime }$)	−2.4	5.6
Inclination of lower lip (li–$sl/y^{\prime }$)	−28.3	−42.5
Inclination of mandible (li–$pg/y^{\prime }$)	−10.7	−15.8
Inclination of upper face (*g*–$sn/y^{\prime }$)	8.7	4.7
Inclination of Leiber line (*g*–$ls/y^{\prime }$)	6.7	4.8
Inclination of lower face (sn–$pg/y^{\prime }$)	−7.9	−9.6
Deviation of nasal bridge (*n*–prn/MSP)	0.7	1.0
Inclination of labial fissure ($ch_{r}$–$ch_{l}/HP$)	0.6	0.1
Inclination of mandibular base ($Go_{r}$–gn)	21.8	22.2
Inclination of mandibular base ($Go_{l}$–gn)	21.8	22.4
Horizontal eye angle ($ex_{r}$–$en_{r}$)	−2.4	−0.4
Horizontal eye angle ($ex_{l}$–$en_{l}$)	−1.9	0.0

**Table 13:** Asymmetry indices (ASX) extracted for the male and female normotypes. Blue values indicate measurements that fall outside the established normotype ranges.

Measurement	Male	Female
ASX $ex_{l}$–$ex_{r}$	1.0	3.0
ASX $en_{l}$–$en_{r}$	1.1	2.0
ASX $t_{l}$–$t_{r}$	0.5	8.2
ASX $zy_{l}$–$zy_{r}$	1.4	4.0
ASX $al_{l}$–$al_{r}$	1.3	1.4
ASX $cph_{l}$–$cph_{r}$	1.0	0.9
ASX $ch_{l}$–$ch_{r}$	0.7	2.9
ASX $Go_{l}$–$Go_{r}$	0.4	6.2
ASX tr	0.7	2.0
ASX *g*	1.2	0.3
ASX *n*	0.0	0.0
ASX se	0.2	0.3
ASX prn	0.6	0.6
ASX sn	0.9	0.1
ASX ls	0.4	0.5
ASX sto	0.0	0.1
ASX li	0.1	0.4
ASX sl	0.2	0.8
ASX pg	0.1	0.7
ASX gn	0.1	0.8

## Data Availability

The research data supporting the findings of this study are available from the corresponding author upon reasonable request.

## Supplementary Materials

The following supporting information can be downloaded at https://www.mdpi.com/article/10.3390/jcm15197518/s1. This section reports the normotypical data relating to the main facial anthropometric measurements, both linear and angular, identified in the selected literature. Linear measurements are reported in Tables S1–S112 and angular measurements are reported in Tables S113–S183. Table S1: Height of the nose: (n-sn), male subjects. Normotypical values of the linear measurement derived from the literature, expressed in millimetres. Table S2: Height of the nose: (n-sn), female subjects. Normotypical values of the linear measurement derived from the literature, expressed in millimetres. Table S3: Width of the mouth: (chr-chl), male subjects. Normotypical values of the linear measurement derived from the literature, expressed in millimetres. Table S4: Width of the mouth: (chr-chl), female subjects. Normotypical values of the linear measurement derived from the literature, expressed in millimetres. Table S5: Biocular width: (exr-exl), male subjects. Normotypical values of the linear measurement derived from the literature, expressed in millimetres. Table S6: Biocular width: (exr-exl), female subjects. Normotypical values of the linear measurement derived from the literature, expressed in millimetres. Table S7: Anterior lower facial height: (sn-pg), male subjects. Normotypical values of the linear measurement derived from the literature, expressed in millimetres. Table S8: Anterior lower facial height: (sn-pg), female subjects. Normotypical values of the linear measurement derived from the literature, expressed in millimetres. Table S9: Skull-base width: (tr-tl), male subjects. Normotypical values of the linear measurement derived from the literature, expressed in millimetres. Table S10: Skull-base width: (tr-tl), female subjects. Normotypical values of the linear measurement derived from the literature, expressed in millimetres. Table S11: Intercanthal width: (enr-enl), male subjects. Normotypical values of the linear measurement derived from the literature, expressed in millimetres. Table S12: Intercanthal width: (enr-enl), female subjects. Normotypical values of the linear measurement derived from the literature, expressed in millimetres. Table S13: Width between the facial insertion points of the alar base: (acr-acl), male subjects. Normotypical values of the linear measurement derived from the literature, expressed in millimetres. Table S14: Width between the facial insertion points of the alar base: (acr-acl), female subjects. Normotypical values of the linear measurement derived from the literature, expressed in millimetres. Table S15: Morphological height of the face: (n-gn), male subjects. Normotypical values of the linear measurement derived from the literature, expressed in millimetres. Table S16: Morphological height of the face: (n-gn), female subjects. Normotypical values of the linear measurement derived from the literature, expressed in millimetres. Table S17: Height of the lower face: (sn-gn), male subjects. Normotypical values of the linear measurement derived from the literature, expressed in millimetres. Table S18: Height of the lower face: (sn-gn), female subjects. Normotypical values of the linear measurement derived from the literature, expressed in millimetres. Table S19: Vermilion height of the upper lip: (ls-sto), male subjects. Normotypical values of the linear measurement derived from the literature, expressed in millimetres. Table S20: Vermilion height of the upper lip: (ls-sto), female subjects. Normotypical values of the linear measurement derived from the literature, expressed in millimetres. Table S21: Vermilion height of the lower lip: (li-sto), male subjects. Normotypical values of the linear measurement derived from the literature, expressed in millimetres. Table S22: Vermilion height of the lower lip: (li-sto), female subjects. Normotypical values of the linear measurement derived from the literature, expressed in millimetres. Table S23: Height of the upper lip: (sn-sto), male subjects. Normotypical values of the linear measurement derived from the literature, expressed in millimetres. Table S24: Height of the upper lip: (sn-sto), female subjects. Normotypical values of the linear measurement derived from the literature, expressed in millimetres. Table S25: Height of forehead II: (tr-n), male subjects. Normotypical values of the linear measurement derived from the literature, expressed in millimetres. Table S26: Height of forehead II: (tr-n), female subjects. Normotypical values of the linear measurement derived from the literature, expressed in millimetres. Table S27: Width of the mandible: (Gor-Gol), male subjects. Normotypical values of the linear measurement derived from the literature, expressed in millimetres. Table S28: Width of the mandible: (Gor-Gol), female subjects. Normotypical values of the linear measurement derived from the literature, expressed in millimetres. Table S29: Depth in the maxillary region (right/left): (sn-t), male subjects. Normotypical values of the linear measurement derived from the literature, expressed in millimetres. Table S30: Depth in the maxillary region (right/left): (sn-t), female subjects. Normotypical values of the linear measurement derived from the literature, expressed in millimetres. Table S31: Depth of the upper third of the face (right/left): (n-t), male subjects. Normotypical values of the linear measurement derived from the literature, expressed in millimetres. Table S32: Depth of the upper third of the face (right/left): (n-t), female subjects. Normotypical values of the linear measurement derived from the literature, expressed in millimetres. Table S33: Length of the eye fissure (right/left): (ex-en), male subjects. Normotypical values of the linear measurement derived from the literature, expressed in millimetres. Table S34: length of the eye fissure (right/left): (ex-en), female subjects. Normotypical values of the linear measurement derived from the literature, expressed in millimetres. Table S35: Length of the nasal bridge: (n-prn), male subjects. Normotypical values of the linear measurement derived from the literature, expressed in millimetres. Table S36: Length of the nasal bridge: (n-prn), female subjects. Normotypical values of the linear measurement derived from the literature, expressed in millimetres. Table S37: Nasal tip protrusion: (sn-prn), male subjects. Normotypical values of the linear measurement derived from the literature, expressed in millimetres. Table S38: Nasal tip protrusion: (sn-prn), female subjects. Normotypical values of the linear measurement derived from the literature, expressed in millimetres. Table S39: Height of the coutaneous upper lip: (sn-ls), male subjects. Normotypical values of the linear measurement derived from the literature, expressed in millimetres. Table S40: Height of the coutaneous upper lip: (sn-ls), female subjects. Normotypical values of the linear measurement derived from the literature, expressed in millimetres. Table S41: Width of the face: (zyr-zyl), male subjects. Normotypical values of the linear measurement derived from the literature, expressed in millimetres. Table S42: Width of the face: (zyr-zyl), female subjects. Normotypical values of the linear measurement derived from the literature, expressed in millimetres. Table S43: Lower half of the cranial height (right/left): (en-gn), male subjects. Normotypical values of the linear measurement derived from the literature, expressed in millimetres. Table S44: Lower half of the cranial height (right/left): (en-gn), female subjects. Normotypical values of the linear measurement derived from the literature, expressed in millimetres. Table S45: Height of forehead I: (tr-g), male subjects. Normotypical values of the linear measurement derived from the literature, expressed in millimetres. Table S46: Height of forehead I: (tr-g), female subjects. Normotypical values of the linear measurement derived from the literature, expressed in millimetres. Table S47: Distance between the glabella and the subnasale: (g-sn), male subjects. Normotypical values of the linear measurement derived from the literature, expressed in millimetres. Table S48: Distance between the glabella and the subnasale: (g-sn), female subjects. Normotypical values of the linear measurement derived from the literature, expressed in millimetres. Table S49: Width of the nose: (alr-all), male subjects. Normotypical values of the linear measurement derived from the literature, expressed in millimetres. Table S50: Width of the nose: (alr-all), female subjects. Normotypical values of the linear measurement derived from the literature, expressed in millimetres. Table S51: Width of the nasal root: (m fr-m fl), male subjects. Normotypical values of the linear measurement derived from the literature, expressed in millimetres. Table S52: Width of the nasal root: (m fr-m fl), female subjects. Normotypical values of the linear measurement derived from the literature, expressed in millimetres. Table S53: Columella length: (sn-c), male subjects. Normotypical values of the linear measurement derived from the literature, expressed in millimetres. Table S54: Columella length: (sn-c), female subjects. Normotypical values of the linear measurement derived from the literature, expressed in millimetres. Table S55: Physiognomical height of the face: (tr-gn), male subjects. Normotypical values of the linear measurement derived from the literature, expressed in millimetres. Table S56: Physiognomical height of the face: (tr-gn), female subjects. Normotypical values of the linear measurement derived from the literature, expressed in millimetres. Table S57: Height of the chin: (sl-gn), male subjects. Normotypical values of the linear measurement derived from the literature, expressed in millimetres. Table S58: Height of the chin: (sl-gn), female subjects. Normotypical values of the linear measurement derived from the literature, expressed in millimetres. Table S59: Depth in the mandibular region (right/left): (gn-t), male subjects. Normotypical values of the linear measurement derived from the literature, expressed in millimetres. Table S60: Depth in the mandibular region (right/left): (gn-t), female subjects. Normotypical values of the linear measurement derived from the literature, expressed in millimetres. Table S61: Physiognomical height of the upper face: (n-sto), male subjects. Normotypical values of the linear measurement derived from the literature, expressed in millimetres. Table S62: Physiognomical height of the upper face: (n-sto), female subjects. Normotypical values of the linear measurement derived from the literature, expressed in millimetres. Table S63: Width between the labial insertions of the alar base: (sbalr-sball), male subjects. Normotypical values of the linear measurement derived from the literature, expressed in millimetres. Table S64: Width between the labial insertions of the alar base: (sbalr-sball), female subjects. Normotypical values of the linear measurement derived from the literature, expressed in millimetres. Table S65: Length of the ala (right/left): (prn-ac), male subjects. Normotypical values of the linear measurement derived from the literature, expressed in millimetres. Table S66: Length of the ala (right/left): (prn-ac), female subjects. Normotypical values of the linear measurement derived from the literature, expressed in millimetres. Table S67: Width of the philtrum: (cphr-cphl), male subjects. Normotypical values of the linear measurement derived from the literature, expressed in millimetres. Table S68: Width of the philtrum: (cphr-cphl), female subjects. Normotypical values of the linear measurement derived from the literature, expressed in millimetres. Table S69: Lower Lip Height: (sto-sl), male subjects. Normotypical values of the linear measurement derived from the literature, expressed in millimetres. Table S70: Lower Lip Height: (sto-sl), female subjects. Normotypical values of the linear measurement derived from the literature, expressed in millimetres. Table S71: Height of the coutaneous lower lip: (li-sl), male subjects. Normotypical values of the linear measurement derived from the literature, expressed in millimetres. Table S72: Height of the coutaneous lower lip: (li-sl), female subjects. Normotypical values of the linear measurement derived from the literature, expressed in millimetres. Table S73: Width of the forehead: (f tr-f tl), male subjects. Normotypical values of the linear measurement derived from the literature, expressed in millimetres. Table S74: Width of the forehead: (f tr-f tl), female subjects. Normotypical values of the linear measurement derived from the literature, expressed in millimetres. Table S75: Depth of the lower jaw (right/left): (gn-Go), male subjects. Normotypical values of the linear measurement derived from the literature, expressed in millimetres. Table S76: Depth of the lower jaw (right/left): (gn-Go), female subjects. Normotypical values of the linear measurement derived from the literature, expressed in millimetres. Table S77: Orbito-glabellar distance (right/left): (ex-g), male subjects. Normotypical values of the linear measurement derived from the literature, expressed in millimetres. Table S78: Orbito-glabellar distance (right/left): (ex-g), female subjects. Normotypical values of the linear measurement derived from the literature, expressed in millimetres. Table S79: Height of the mandible: (sto-gn), male subjects. Normotypical values of the linear measurement derived from the literature, expressed in millimetres. Table S80: Height of the mandible: (sto-gn), female subjects. Normotypical values of the linear measurement derived from the literature, expressed in millimetres. Table S81: Width of the nostril floor (right/left): (sbal-sn), male subjects. Normotypical values of the linear measurement derived from the literature, expressed in millimetres. Table S82: Width of the nostril floor (right/left): (sbal-sn), female subjects. Normotypical values of the linear measurement derived from the literature, expressed in millimetres. Table S83: Height of the upper profile: (tr-prn), male subjects. Normotypical values of the linear measurement derived from the literature, expressed in millimetres. Table S84: Height of the upper profile: (tr-prn), female subjects. Normotypical values of the linear measurement derived from the literature, expressed in millimetres. Table S85: Height of the lower profile: (prn-gn), male subjects. Normotypical values of the linear measurement derived from the literature, expressed in millimetres. Table S86: Height of the lower profile: (prn-gn), female subjects. Normotypical values of the linear measurement derived from the literature, expressed in millimetres. Table S87: Height of the mandibular ramus (right/left): (Go-cdl), male subjects. Normotypical values of the linear measurement derived from the literature, expressed in millimetres. Table S88: Height of the mandibular ramus (right/left): (Go-cdl), female subjects. Normotypical values of the linear measurement derived from the literature, expressed in millimetres. Table S89: Depth of the supraorbital rim (right/left): (t-g), male subjects. Normotypical values of the linear measurement derived from the literature, expressed in millimetres. Table S90: Depth of the supraorbital rim (right/left): (t-g), female subjects. Normotypical values of the linear measurement derived from the literature, expressed in millimetres. Table S91: Endocanthion-facial midline distance (right/left): (en-se), male subjects. Normotypical values of the linear measurement derived from the literature, expressed in millimetres. Table S92: Endocanthion-facial midline distance (right/left): (en-se), female subjects. Normotypical values of the linear measurement derived from the literature, expressed in millimetres. Table S93: Orbitotragial distance (right/left): (ex-t), male subjects. Normotypical values of the linear measurement derived from the literature, expressed in millimetres. Table S94: Orbitotragial distance (right/left): (ex-t), female subjects. Normotypical values of the linear measurement derived from the literature, expressed in millimetres. Table S95: Orbitogonial distance (right/left): (ex-Go), male subjects. Normotypical values of the linear measurement derived from the literature, expressed in millimetres. Table S96: Orbitogonial distance (right/left): (ex-Go), female subjects. Normotypical values of the linear measurement derived from the literature, expressed in millimetres. Table S97: Combined height of orbit and eyebrow (right/left): (or-sci), male subjects. Normotypical values of the linear measurement derived from the literature, expressed in millimetres. Table S98: Combined height of orbit and eyebrow (right/left): (or-sci), female subjects. Normotypical values of the linear measurement derived from the literature, expressed in millimetres. Table S99: Height of eye fissure (right/left): (ps-pi), male subjects. Normotypical values of the linear measurement derived from the literature, expressed in millimetres. Table S100: Height of eye fissure: (ps-pi), female subjects. Normotypical values of the linear measurement derived from the literature, expressed in millimetres. Table S101: Height of the upper lid (right/left): (ps-os), male subjects. Normotypical values of the linear measurement derived from the literature, expressed in millimetres. Table S102: Height of the upper lid: (ps-os), female subjects. Normotypical values of the linear measurement derived from the literature, expressed in millimetres. Table S103: Height of the lower lid (right/left): direct linear measurment (pi-or), male subjects. Normotypical values of the linear measurement derived from the literature, expressed in millimetres. Table S104: Height of the lower lid (right/left): (pi-or), female subjects. Normotypical values of the linear measurement derived from the literature, expressed in millimetres. Table S105: Width of the columella: (sn’r-sn’l), male subjects. Normotypical values of the linear measurement derived from the literature, expressed in millimetres. Table S106: Width of the columella: (sn’r-sn’l), female subjects. Normotypical values of the linear measurement derived from the literature, expressed in millimetres. Table S107: Distance between the facial insertions of the alar base and the subnasal point (right/left): (ac-sn), male subjects. Normotypical values of the linear measurement derived from the literature, expressed in millimetres. Table S108: Distance between the facial insertions of the alar base and the subnasal point (right/left): (ac-sn), female subjects. Normotypical values of the linear measurement derived from the literature, expressed in millimetres. Table S109: Labio-aural distance (right/left): (ch-t), male subjects. Normotypical values of the linear measurement derived from the literature, expressed in millimetres. Table S110: Labio-aural distance (right/left): (ch-t), female subjects. Normotypical values of the linear measurement derived from the literature, expressed in millimetres. Table S111: Height of the orbit (right/left): (or-os), male subjects. Normotypical values of the linear measurement derived from the literature, expressed in millimetres. Table S112: Height of the orbit (right/left): (or-os), female subjects. Normotypical values of the linear measurement derived from the literature, expressed in millimetres. Table S113: Angle of facial convexity (n-sn-pg), male subjects. Normotypical values of the angular measurement derived from the literature, expressed in degrees. Table S114: Angle of facial convexity (n-sn-pg), female subjects. Normotypical values of the angular measurement derived from the literature, expressed in degrees. Table S115: Nasomental angle (n-prn-pg), male subjects. Normotypical values of the angular measurement derived from the literature, expressed in degrees. Table S116: Nasomental angle (n-prn-pg), female subjects. Normotypical values of the angular measurement derived from the literature, expressed in degrees. Table S117: Exocanthion–nasion angle (exl-n-exr), male subjects. Normotypical values of the angular measurement derived from the literature, expressed in degrees. Table S118: Exocanthion–nasion angle (exl-n-exr), female subjects. Normotypical values of the angular measurement derived from the literature, expressed in degrees. Table S119: Nasofrontal angle (g-n-prn), male subjects. Normotypical values of the angular measurement derived from the literature, expressed in degrees. Table S120: Nasofrontal angle (g-n-prn), female subjects. Normotypical values of the angular measurement derived from the literature, expressed in degrees. Table S121: Nasal tip angle (n-prn-sn), male subjects. Normotypical values of the angular measurement derived from the literature, expressed in degrees. Table S122: Nasal tip angle (n-prn-sn), female subjects. Normotypical values of the angular measurement derived from the literature, expressed in degrees. Table S123: Angle of total facial convexity (g-sn-pg), male subjects. Normotypical values of the angular measurement derived from the literature, expressed in degrees. Table S124: Angle of total facial convexity (g-sn-pg), female subjects. Normotypical values of the angular measurement derived from the literature, expressed in degrees. Table S125: Nasofacial angle (g-prn-pg), male subjects. Normotypical values of the angular measurement derived from the literature, expressed in degrees. Table S126: Nasofacial angle (g-prn-pg), female subjects. Normotypical values of the angular measurement derived from the literature, expressed in degrees. Table S127: Tragion–nasion–tragion angle (tl-n-tr), male subjects. Normotypical values of the angular measurement derived from the literature, expressed in degrees. Table S128: Tragion–nasion–tragion angle (tl-n-tr), female subjects. Normotypical values of the angular measurement derived from the literature, expressed in degrees. Table S129: Tragion–pronasale–tragion angle (tl-prn-tr), male subjects. Normotypical values of the angular measurement derived from the literature, expressed in degrees. Table S130: Tragion–pronasale–tragion angle (tl-prn-tr), female subjects. Normotypical values of the angular measurement derived from the literature, expressed in degrees. Table S131: Tragion–pogonion–tragion angle (tl-pg-tr), male subjects. Normotypical values of the angular measurement derived from the literature, expressed in degrees. Table S132: Tragion–pogonion–tragion angle (tl-pg-tr), female subjects. Normotypical values of the angular measurement derived from the literature, expressed in degrees. Table S133: Nasolabial angle (c-sn-ls), male subjects. Normotypical values of the angular measurement derived from the literature, expressed in degrees. Table S134: Nasolabial angle (c-sn-ls), female subjects. Normotypical values of the angular measurement derived from the literature, expressed in degrees. Table S135: Labiomental angle (li-sl-pg), male subjects. Normotypical values of the angular measurement derived from the literature, expressed in degrees. Table S136: Labiomental angle (li-sl-pg), female subjects. Normotypical values of the angular measurement derived from the literature, expressed in degrees. Table S137: Midface convexity angle (right/left) (n-t-sn), male subjects. Normotypical values of the angular measurement derived from the literature, expressed in degrees. Table S138: Midface convexity angle (right/left) (n-t-sn), female subjects. Normotypical values of the angular measurement derived from the literature, expressed in degrees. Table S139: Inclination of the upper face profile (g-sn/y-plane), male subjects. Normotypical values of the angular measurement derived from the literature, expressed in degrees. Table S140: Inclination of the upper face profile (g-sn/y-plane), female subjects. Normotypical values of the angular measurement derived from the literature, expressed in degrees. Table S141: Inclination of the anterior surface of the forehead (tr-g/y-plane), male subjects. Normotypical values of the angular measurement derived from the literature, expressed in degrees. Table S142: Inclination of the anterior surface of the forehead (tr-g/y-plane), female subjects. Normotypical values of the angular measurement derived from the literature, expressed in degrees. Table S143: Inclination of the Leiber line (g-ls/y-plane), male subjects. Normotypical values of the angular measurement derived from the literature, expressed in degrees. Table S144: Inclination of the Leiber line (g-ls/y-plane), female subjects. Normotypical values of the angular measurement derived from the literature, expressed in degrees. Table S145: Inclination of the lower face profile (sn-pg/y-plane), male subjects. Normotypical values of the angular measurement derived from the literature, expressed in degrees. Table S146: Inclination of the lower face profile (sn-pg/y-plane), female subjects. Normotypical values of the angular measurement derived from the literature, expressed in degrees. Table S147: Inclination of the mandible (li-pg/y-plane), male subjects. Normotypical values of the angular measurement derived from the literature, expressed in degrees. Table S148: Inclination of the mandible (li-pg/y-plane), female subjects. Normotypical values of the angular measurement derived from the literature, expressed in degrees. Table S149: Inclination of the chin (sl-pg/y-plane), male subjects. Normotypical values of the angular measurement derived from the literature, expressed in degrees. Table S150: Inclination of the chin (sl-pg/y-plane), female subjects. Normotypical values of the angular measurement derived from the literature, expressed in degrees. Table S151: Inclination of the general profile line (g-pg/y-plane), male subjects. Normotypical values of the angular measurement derived from the literature, expressed in degrees. Table S152: Inclination of the general profile line (g-pg/y-plane), female subjects. Normotypical values of the angular measurement derived from the literature, expressed in degrees. Table S153: Mentocervical angle (sl-pg/gn-gn’), male subjects. Normotypical values of the angular measurement derived from the literature, expressed in degrees. Table S154: Mentocervical angle (sl-pg/gn-gn’), female subjects. Normotypical values of the angular measurement derived from the literature, expressed in degrees. Table S155: Inclination of the nasal bridge (n-prn/y-plane), male subjects. Normotypical values of the angular measurement derived from the literature, expressed in degrees. Table S156: Inclination of the nasal bridge (n-prn/y-plane), female subjects. Normotypical values of the angular measurement derived from the literature, expressed in degrees. Table S157: Inclination of the columella (sn-c/y-plane), male subjects. Normotypical values of the angular measurement derived from the literature, expressed in degrees. Table S158: Inclination of the columella (sn-c/y-plane), female subjects. Normotypical values of the angular measurement derived from the literature, expressed in degrees. Table S159: Inclination of the nasal tip portion (c-prn/y-plane), male subjects. Normotypical values of the angular measurement derived from the literature, expressed in degrees. Table S160: Inclination of the nasal tip portion (c-prn/y-plane), female subjects. Normotypical values of the angular measurement derived from the literature, expressed in degrees. Table S161: Nasal root-slope angle (m fr-se-m fl), male subjects. Normotypical values of the angular measurement derived from the literature, expressed in degrees. Table S162: Nasal root-slope angle (m fr-se-m fl), female subjects. Normotypical values of the angular measurement derived from the literature, expressed in degrees. Table S163: Deviation of the nasal bridge (n-prn/z-plane), male subjects. Normotypical values of the angular measurement derived from the literature, expressed in degrees. Table S164: Deviation of the nasal bridge (n-prn/z-plane), female subjects. Normotypical values of the angular measurement derived from the literature, expressed in degrees. Table S165: Deviation of the columella (sn-c/z-plane), male subjects. Normotypical values of the angular measurement derived from the literature, expressed in degrees. Table S166: Deviation of the columella (sn-c/z-plane), female subjects. Normotypical values of the angular measurement derived from the literature, expressed in degrees. Table S167: Inclination of the labial fissure (chr-chl/x-plane), male subjects. Normotypical values of the angular measurement derived from the literature, expressed in degrees. Table S168: Inclination of the labial fissure (chr-chl/x-plane), female subjects. Normotypical values of the angular measurement derived from the literature, expressed in degrees. Table S169: Inclination of the upper lip (sn-ls/y-plane), male subjects. Normotypical values of the angular measurement derived from the literature, expressed in degrees. Table S170: Inclination of the upper lip (sn-ls/y-plane), female subjects. Normotypical values of the angular measurement derived from the literature, expressed in degrees. Table S171: Inclination of the lower lip (li-sl/y-plane), male subjects. Normotypical values of the angular measurement derived from the literature, expressed in degrees. Table S172: Inclination of the lower lip (li-sl/y-plane), female subjects. Normotypical values of the angular measurement derived from the literature, expressed in degrees. Table S173: Nasal convexity angle (sl-n-sn), female subjects. Normotypical values of the angular measurement derived from the literature, expressed in degrees. Table S174: Variant of nasolabial angle (prn-sn-ls), female subjects. Normotypical values of the angular measurement derived from the literature, expressed in degrees. Table S175: Deviation of the columella (sn-c/z-plane), female subjects. Normotypical values of the angular measurement derived from the literature, expressed in degrees. Table S176: Inclination of the labial fissure (chr-chl/x-plane), male subjects. Normotypical values of the angular measurement derived from the literature, expressed in degrees. Table S177: Inclination of the labial fissure (chr-chl/x-plane), female subjects. Normotypical values of the angular measurement derived from the literature, expressed in degrees. Table S178: Inclination of the upper lip (sn-ls/y-plane), male subjects. Normotypical values of the angular measurement derived from the literature, expressed in degrees. Table S179: Inclination of the upper lip (sn-ls/y-plane), female subjects. Normotypical values of the angular measurement derived from the literature, expressed in degrees. Table S180: Inclination of the lower lip (li-sl/y-plane), male subjects. Normotypical values of the angular measurement derived from the literature, expressed in degrees. Table S181: Inclination of the lower lip (li-sl/y-plane), female subjects. Normotypical values of the angular measurement derived from the literature, expressed in degrees. Table S182: Nasal convexity angle (sl-n-sn), female subjects. Normotypical values of the angular measurement derived from the literature, expressed in degrees. Table S183: Variant of nasolabial angle (prn-sn-ls), female subjects. Normotypical values of the angular measurement derived from the literature, expressed in degrees. A dedicated table has been created for each measurement. The caption of each table indicates the name of the measurement, the anthropometric landmarks involved, and the reference sex. Measurements are presented separately for male and female subjects. Within each table, the first column shows the sample size used to calculate the measurement in a specific study; the second column indicates the mean value; the third column shows the standard deviation; the fourth and fifth columns show the minimum and maximum values observed, respectively; the sixth column describes the method of measurement used in the study; the seventh column indicates the reference population: if the article specifies ethnicity but not specific geographical origin, “Caucasian” is reported, and if the nationality of the sample is indicated, this is reported; and finally, the eighth column shows the bibliographic reference. This organization allows for a direct comparison of the results from different studies, highlighting any variations related to sample size, acquisition methodology and characteristics of the population analyzed, and forms the basis for the presented methodology.
