# Supplementary material for: 3D Facial Normotypes for Maxillofacial Surgical Planning
Source: J Clin Med. 2026 Sep 27;15(19):7518. doi: 10.3390/jcm15197518 (PMC13666107; doi:10.3390/jcm15197518)
Supplement: Supplementary file 1 [file jcm-15-07518-s001.zip › jcm-4515922-supplementary.pdf]

Supplementary Material
3D Facial Normotypes for Maxillofacial Surgical Planning

Table S1: Height of the nose: (n-sn), male subjects. Normotypical values of the linear measurement derived from the literature, expressed in millimetres.

N	Mean	SD	Min	Max	Method	Population	Reference
40	64.3	5.0	-	-	infrared camera	Caucasian	[1]
-	53.0	3.5	46.0	60.0	direct anthropometry	North American White	[2]
30	55.9	3.4	49.1	62.7	direct anthropometry	Azerbaijan	[2]
30	54.0	3.8	46.4	61.6	direct anthropometry	Bulgarian	[2]
30	54.0	4.1	45.8	62.2	direct anthropometry	Chez	[2]
30	53.8	3	47.8	59.8	direct anthropometry	Croatian	[2]
30	52.0	5.6	40.8	63.2	direct anthropometry	German	[2]
30	55.5	4.1	47.3	63.7	direct anthropometry	Greek	[2]
30	55.0	2.6	49.8	60.2	direct anthropometry	Hungarian	[2]
30	56.2	3.9	48.4	64.0	direct anthropometry	Italian	[2]
30	53.7	3.4	46.9	60.5	direct anthropometry	Polish	[2]
30	59.5	3.9	51.7	67.3	direct anthropometry	Portuguese	[2]
30	51.7	2.6	46.5	56.9	direct anthropometry	Russian	[2]
30	53.5	2.8	47.9	59.1	direct anthropometry	Slovak	[2]
30	56.2	4	48.2	64.2	direct anthropometry	Slovenian	[2]
30	58.1	3.5	51.1	65.1	direct anthropometry	Turkish	[2]
271	51.1	-	-	-	photogrammetry	Caucasian	[3]
535	56.6	3.9	-	-	digital stereophotogrammetry	Caucasian	[4]
100	53.1	4.4	40.1	62.9	direct anthropometry	Turkish	[5]
-	53.2	3.3	-	-	direct anthropometry	North American White	[5]
149	51.9	7.5	-	-	photographic analysis	Turkish	[6]
109	54.8	3.3	-	-	direct anthropometry	North American White	[7]
228	52.4	4.0	44.0	71.0	direct anthropometry	Turkish	[8]
25	57.3	4.8	50.0	72.0	direct anthropometry	Turkish	[9]
29	56.2	3.9	51.0	64.0	direct anthropometry	Turkish	[9]
36	55.9	4.5	48.0	69.0	direct anthropometry	Turkish	[9]

Table S2: Height of the nose: (n-sn), female subjects. Normotypical values of the linear measurement derived from the literature, expressed in millimetres.

N	Mean	SD	Min	Max	Method	Population	Reference
40	62.0	4.0	-	-	infrared camera	Caucasian	[1]
66	50.3	2.4	41.9	54.7	3D facial scanner	Caucasian	[10]
-	48.9	2.6	43.7	54.1	direct anthropometry	North American White	[2]
30	52.3	3.6	45.1	59.5	direct anthropometry	Azerbaijan	[2]
30	52.1	3.2	45.7	58.5	direct anthropometry	Bulgarian	[2]
30	52.1	3.7	44.7	59.5	direct anthropometry	Chez	[2]
30	50.0	3.6	42.8	57.2	direct anthropometry	Croatian	[2]
30	51.4	2.8	45.8	57.0	direct anthropometry	German	[2]
30	52.8	3.3	46.2	59.4	direct anthropometry	Greek	[2]
30	52.5	3.5	45.5	59.5	direct anthropometry	Hungarian	[2]
30	52.1	6.8	38.5	65.7	direct anthropometry	Italian	[2]
30	51.2	3.2	44.8	57.6	direct anthropometry	Polish	[2]
30	57.8	4.4	49.0	66.6	direct anthropometry	Portuguese	[2]
30	50.4	3.4	43.6	57.2	direct anthropometry	Russian	[2]
30	49.4	2.4	44.6	54.2	direct anthropometry	Slovak	[2]
30	52.0	2.3	47.4	56.6	direct anthropometry	Slovenian	[2]
30	55.2	4	47.2	63.2	direct anthropometry	Turkish	[2]
830	48.5	-	-	-	photogrammetry	Caucasian	[3]
907	54.7	3.8	-	-	digital stereophotogrammetry	Caucasian	[4]
100	50.4	3.7	42.8	60.3	direct anthropometry	Turkish	[5]
281	51.7	5.8	-	-	photographic analysis	Turkish	[6]
200	50.6	3.1	-	-	direct anthropometry	North American White	[7]
-	49.2	2.9	-	-	direct anthropometry	North American White	[5]
34	51.3	3.1	46.0	59.0	direct anthropometry	Turkish	[9]
20	53.9	3.9	48.0	62.0	direct anthropometry	Turkish	[9]
29	54.0	3.1	47.0	60.0	direct anthropometry	Turkish	[9]
272	49.7	4.2	41.0	84.0	direct anthropometry	Turkish	[8]

Table S3: Width of the mouth: (ch_r-ch_l), male subjects. Normotypical values of the linear measurement derived from the literature, expressed in millimetres.

N	Mean	SD	Min	Max	Method	Population	Reference
40	54.9	4.0	-	-	infrared camera	Caucasian	[1]
-	53.3	3.3	46.7	59.9	direct anthropometry	North American White	[2]
30	53.4	3.5	46.4	60.4	direct anthropometry	Azerbaijan	[2]
30	49.8	3.8	42.2	57.4	direct anthropometry	Bulgarian	[2]
30	53.8	3.6	46.6	61.0	direct anthropometry	Chez	[2]
30	50.5	3.9	42.7	58.3	direct anthropometry	Croatian	[2]
30	50.9	3.4	44.1	57.7	direct anthropometry	German	[2]
30	51.8	4.7	42.4	61.2	direct anthropometry	Greek	[2]
30	57.0	4.1	48.8	65.2	direct anthropometry	Hungarian	[2]
30	50.8	3.8	43.2	58.4	direct anthropometry	Italian	[2]

Table S3 – Continued from previous page

N	Mean	SD	Min	Max	Method	Population	Reference
30	52.5	3.2	46.1	58.9	direct anthropometry	Polish	[2]
30	50.0	4.1	41.8	58.2	direct anthropometry	Portuguese	[2]
30	52.5	3.8	44.9	60.1	direct anthropometry	Russian	[2]
30	53.2	4	45.2	61.2	direct anthropometry	Slovak	[2]
30	53.0	3.9	45.2	60.8	direct anthropometry	Slovenian	[2]
30	53.0	3.2	46.6	59.4	direct anthropometry	Turkish	[2]
149	48.2	-	-	-	photogrammetry	Caucasian	[3]
535	50.7	3.7	-	-	digital stereophotogrammetry	Caucasian	[4]
50	53.3	3.3	-	-	direct anthropometry	North American White	[11]
100	51.5	4.1	43.1	62.2	direct anthropometry	Turkish	[5]
-	53.3	3.6	-	-	direct anthropometry	North American White	[5]
149	47.1	5.4	-	-	photographic analysis	Turkish	[6]
109	54.5	3.0	-	-	direct anthropometry	North American White	[7]
228	49.4	3.4	41.0	59.0	direct anthropometry	Turkish	[8]
25	51.1	4.1	44.0	63.0	direct anthropometry	Turkish	[9]
29	50.3	3.6	45.0	59.0	direct anthropometry	Turkish	[9]
36	49.1	4.0	41.0	56.0	direct anthropometry	Turkish	[9]

Table S4: Width of the mouth: (ch_r-ch_l), female subjects. Normotypical values of the linear measurement derived from the literature, expressed in millimetres.

N	Mean	SD	Min	Max	Method	Population	Reference
40	51.2	3.8	-	-	infrared camera	Caucasian	[1]
12	49.2	2.1	-	-	computerized electromagnetic digitizer	Caucasian	[12]
12	48.2	2.9	-	-	computerized electromagnetic digitizer	Caucasian	[12]
71	48.5	3.5	-	-	computerized electromagnetic digitizer	Caucasian	[12]
66	45.7	2.8	38.5	54.3	3D facial scanner	Caucasian	[10]
-	49.8	3.2	43.4	56.2	direct anthropometric measurements	North American White	[2]
30	49.7	3.7	42.3	57.1	direct anthropometry	Azerbaijan	[2]
30	46.2	2.1	42.0	50.4	direct anthropometry	Bulgarian	[2]
30	50.2	3.4	43.4	57.0	direct anthropometry	Chez	[2]
30	46.9	3.4	40.1	53.7	direct anthropometry	Croatian	[2]
30	48.2	3.5	41.2	55.2	direct anthropometry	German	[2]
30	50.3	4.3	41.7	58.9	direct anthropometry	Greek	[2]
30	51.6	3.8	44.0	59.2	direct anthropometry	Hungarian	[2]
30	47.7	3.8	40.1	55.3	direct anthropometry	Italian	[2]
30	49.0	2.8	43.4	54.6	direct anthropometry	Polish	[2]
30	45.3	2.9	39.5	51.1	direct anthropometry	Portuguese	[2]
30	48.1	3.1	41.9	54.3	direct anthropometry	Russian	[2]
30	48.9	3	42.9	54.9	direct anthropometry	Slovak	[2]
30	49.2	3.2	42.8	55.6	direct anthropometry	Slovenian	[2]
30	47.6	2.9	41.8	53.4	direct anthropometry	Turkish	[2]
490	48.0	-	-	-	photogrammetry	Caucasian	[3]
907	47.8	3.4	-	-	digital stereophotogrammetry	Caucasian	[4]
66	45.7	2.8	38.5	54.3	3D facial scanner	Caucasian	[10]
50	49.8	3.2	-	-	direct anthropometry	North American White	[11]
400	48.1	3.3	-	-	photogrammetry	Caucasian	[13]
100	48.9	3.9	39.9	64.7	direct anthropometry	Turkish	[5]
281	44.0	4.1	-	-	photographic analysis	Turkish	[6]
-	49.8	3.2	-	-	direct anthropometry	North American White	[5]
200	50.2	3.5	-	-	direct anthropometry	North American White	[7]
34	47.3	3.3	39.0	55.0	direct anthropometry	Turkish	[9]
20	48.7	4.7	42.0	56.0	direct anthropometry	Turkish	[9]
29	46.5	4.0	40.0	37.0	direct anthropometry	Turkish	[9]
272	45.4	3.0	35.0	59.0	direct anthropometry	Turkish	[8]

Table S5: Biocular width: (ex_r-ex_l), male subjects. Normotypical values of the linear measurement derived from the literature, expressed in millimetres.

N	Mean	SD	Min	Max	Method	Population	Reference
-	89.4	3.6	82.2	96.6	direct anthropometry	North American White	[2]
30	96.2	4.2	87.8	104.6	direct anthropometry	Azerbaijan	[2]
30	94.7	5.8	83.1	106.3	direct anthropometry	Bulgarian	[2]
30	84.2	3.9	76.4	92.0	direct anthropometry	Chez	[2]
30	92.5	4.7	83.1	101.9	direct anthropometry	Croatian	[2]
30	87.9	4.5	78.9	96.9	direct anthropometry	German	[2]
30	93.7	7.4	78.9	108.5	direct anthropometry	Greek	[2]
30	100.4	5	90.4	110.4	direct anthropometry	Hungarian	[2]
30	93.8	3.5	86.8	100.8	direct anthropometry	Italian	[2]
30	90.2	5	80.2	100.2	direct anthropometry	Polish	[2]
30	99.3	3.5	87.7	101.9	direct anthropometry	Portuguese	[2]
30	98.9	3.9	91.1	106.7	direct anthropometry	Russian	[2]
30	100.7	5.3	90.1	111.3	direct anthropometry	Slovak	[2]
30	102.6	4.4	93.8	111.4	direct anthropometry	Slovenian	[2]
30	95.8	3.1	89.6	102.0	direct anthropometry	Turkish	[2]
528	89.0	3.9	-	-	digital stereophotogrammetry	Caucasian	[4]
50	89.4	3.6	-	-	direct anthropometry	North American White	[11]
100	99.7	5.5	84.4	111.1	direct anthropometry	Turkish	[5]

Table S5 – Continued from previous page

N	Mean	SD	Min	Max	Method	Population	Reference
-	90.7	3.8	-	-	direct anthropometry	North American White	[5]
109	91.2	3.0	-	-	direct anthropometry	North American White	[7]

Table S6: Biocular width: (ex_r-ex_l), female subjects. Normotypical values of the linear measurement derived from the literature, expressed in millimetres.

N	Mean	SD	Min	Max	Method	Population	Reference
12	95.2	3.3	-	-	computerized electromagnetic digitizer	Caucasian	[12]
12	92.5	3.5	-	-	computerized electromagnetic digitizer	Caucasian	[12]
71	90.2	3.8	-	-	computerized electromagnetic digitizer	Caucasian	[12]
66	84.0	3.0	78.7	92.3	3D facial scanner	Caucasian	[10]
-	86.8	4	78.8	94.8	direct anthropometry	North American White	[2]
30	94.2	4	86.2	102.2	direct anthropometry	Azerbaijan	[2]
30	91.9	4.9	82.1	101.7	direct anthropometry	Bulgarian	[2]
30	80.3	4.3	71.7	88.9	direct anthropometry	Chez	[2]
30	86.3	4.8	76.7	95.9	direct anthropometry	Croatian	[2]
30	86.4	3.2	80.0	92.8	direct anthropometry	German	[2]
30	87.8	6.1	75.6	100.0	direct anthropometry	Greek	[2]
30	97.3	4.8	87.7	106.9	direct anthropometry	Hungarian	[2]
30	89.5	4	81.5	97.5	direct anthropometry	Italian	[2]
30	87.4	3.2	81.0	93.8	direct anthropometry	Polish	[2]
30	93.9	6.3	81.3	106.5	direct anthropometry	Portuguese	[2]
30	94.6	3.5	87.6	101.6	direct anthropometry	Russian	[2]
30	96.1	4	88.1	104.1	direct anthropometry	Slovak	[2]
30	96.1	5.1	85.9	106.3	direct anthropometry	Slovenian	[2]
30	93.2	4.5	83.8	101.6	direct anthropometry	Turkish	[2]
895	85.6	3.8	-	-	digital stereophotogrammetry	Caucasian	[4]
50	86.8	4.0	-	-	direct anthropometry	North American White	[11]
100	96.5	4.7	88.1	112.4	direct anthropometry	Turkish	[5]
-	87.6	4.0	-	-	direct anthropometry	North American White	[5]
200	87.8	3.2	-	-	direct anthropometry	North American White	[7]

Table S7: Anterior lower facial height: (sn-pg), male subjects. Normotypical values of the linear measurement derived from the literature, expressed in millimetres.

N	Mean	SD	Min	Max	Method	Population	Reference
40	50.4	4.0	-	-	infrared camera	Caucasian	[1]

Table S8: Anterior lower facial height: (sn-pg), female subjects. Normotypical values of the linear measurement derived from the literature, expressed in millimetres.

N	Mean	SD	Min	Max	Method	Population	Reference
40	46.3	3.4	-	-	infrared camera	Caucasian	[1]
66	48.1	2.6	42.5	56.5	3D facial scanner	Caucasian	[10]

Table S9: Skull-base width: (t_r-t_l), male subjects. Normotypical values of the linear measurement derived from the literature, expressed in millimetres.

N	Mean	SD	Min	Max	Method	Population	Reference
40	143.0	9.2	-	-	infrared camera	Caucasian	[1]
529	147.8	5.3	-	-	digital stereophotogrammetry	Caucasian	[4]
109	146.8	5.6	-	-	direct anthropometry	North American White	[7]

Table S10: Skull-base width: (t_r-t_l), female subjects. Normotypical values of the linear measurement derived from the literature, expressed in millimetres.

N	Mean	SD	Min	Max	Method	Population	Reference
40	132.4	10.1	-	-	infrared camera	Caucasian	[1]
12	136.2	5.2	-	-	computerized electromagnetic digitizer	Caucasian	[12]
12	130.8	4.7	-	-	computerized electromagnetic digitizer	Caucasian	[12]
71	134.4	5.7	-	-	computerized electromagnetic digitizer	Caucasian	[12]
66	131.5	4.2	123.7	140.0	3D facial scanner	Caucasian	[10]
881	138.8	4.9	-	-	digital stereophotogrammetry	Caucasian	[4]
200	138.3	4.9	-	-	direct anthropometry	North American White	[7]

Table S11: Intercanthal width: (en_r-en_l), male subjects. Normotypical values of the linear measurement derived from the literature, expressed in millimetres.

**N**	**Mean**	**SD**	**Min**	**Max**	**Method**	**Population**	**Reference**
-	32.9	2.8	27.5	38.5	direct anthropometry	North American White	[2]
30	30.8	3.5	23.8	37.8	direct anthropometry	Azerbaijan	[2]
30	32.3	3.5	25.3	39.3	direct anthropometry	Bulgarian	[2]
30	30.9	3.1	24.7	37.1	direct anthropometry	Chez	[2]
30	31.5	2.7	26.1	36.9	direct anthropometry	Croatian	[2]
30	31.2	2.6	26.0	36.4	direct anthropometry	German	[2]
30	30.0	3.8	22.8	37.8	direct anthropometry	Greek	[2]
30	31.7	2.3	27.1	36.3	direct anthropometry	Hungarian	[2]
30	30.2	2.4	25.4	35.0	direct anthropometry	Italian	[2]
30	30.3	2.4	25.5	35.1	direct anthropometry	Polish	[2]
30	30.5	3.6	23.3	37.7	direct anthropometry	Portuguese	[2]
30	34.2	1.9	30.4	38.0	direct anthropometry	Russian	[2]
30	32.6	2.8	27.0	38.2	direct anthropometry	Slovak	[2]
30	32.0	2	28.0	36.0	direct anthropometry	Slovenian	[2]
30	32.8	2.6	27.6	38.0	direct anthropometry	Turkish	[2]
534	32.8	2.9	-	-	digital stereophotogrammetry	Caucasian	[4]
50	32.9	2.7	-	-	direct anthropometry	North American White	[11]
100	33.2	2.8	27.5	41.4	direct anthropometry	Turkish	[5]
-	32.9	2.7	-	-	direct anthropometry	North American White	[5]
109	33.3	2.7	-	-	direct anthropometry	North American White	[7]
228	30.7	2.8	25.0	39.0	direct anthropometry	Turkish	[8]
25	32.1	2.9	28.0	38.0	direct anthropometry	Turkish	[9]
29	31.0	3.0	27.0	39.0	direct anthropometry	Turkish	[9]
36	28.9	3.7	20.0	40.0	direct anthropometry	Turkish	[9]

Table S12: Intercanthal width: (en_r-en_l), female subjects. Normotypical values of the linear measurement derived from the literature, expressed in millimetres.

**N**	**Mean**	**SD**	**Min**	**Max**	**Method**	**Population**	**Reference**
66	30.5	2.0	25.1	34.7	3D facial scanner	Caucasian	[10]
-	31.6	2.4	26.8	36.4	direct anthropometry	North American White	[2]
30	30.5	2.2	26.1	34.9	direct anthropometry	Azerbaijan	[2]
30	29.7	2.2	25.3	34.1	direct anthropometry	Bulgarian	[2]
30	29.1	2.4	24.3	33.9	direct anthropometry	Chez	[2]
30	29.7	2.4	24.9	34.5	direct anthropometry	Croatian	[2]
30	28.6	3	22.6	34.6	direct anthropometry	German	[2]
30	29.5	2.5	24.5	34.5	direct anthropometry	Greek	[2]
30	31.2	1.8	27.6	34.8	direct anthropometry	Hungarian	[2]
30	27.6	3.2	21.2	34.0	direct anthropometry	Italian	[2]
30	29.2	2.2	24.8	33.6	direct anthropometry	Polish	[2]
30	29.1	2.8	23.5	34.7	direct anthropometry	Portuguese	[2]
30	32.7	3	26.7	38.7	direct anthropometry	Russian	[2]
30	30.7	2.4	25.9	35.5	direct anthropometry	Slovak	[2]
30	30.2	2.8	24.6	35.8	direct anthropometry	Slovenian	[2]
30	31.7	2.2	27.3	36.1	direct anthropometry	Turkish	[2]
906	31.3	2.7	-	-	digital stereophotogrammetry	Caucasian	[4]
50	31.6	2.4	-	-	direct anthropometry	North American White	[11]
100	31.9	2.4	26.9	38.1	direct anthropometry	Turkish	[5]
-	32.5	2.1	-	-	direct anthropometry	North American White	[5]
200	31.8	2.3	-	-	direct anthropometry	North American White	[7]
34	30.0	3.2	24.0	36.0	direct anthropometry	Turkish	[9]
20	30.9	1.9	28.0	34.0	direct anthropometry	Turkish	[9]
29	30.0	2.7	25.0	35.0	direct anthropometry	Turkish	[9]
272	30.0	2.4	23.0	39.0	direct anthropometry	Turkish	[8]

Table S13: Width between the facial insertion points of the alar base: (ac_r-ac_l), male subjects. Normotypical values of the linear measurement derived from the literature, expressed in millimetres.

**N**	**Mean**	**SD**	**Min**	**Max**	**Method**	**Population**	**Reference**
86	32.8	2.3	-	-	direct anthropometry	North American White	[7]

Table S14: Width between the facial insertion points of the alar base: (ac_r-ac_l), female subjects. Normotypical values of the linear measurement derived from the literature, expressed in millimetres.

**N**	**Mean**	**SD**	**Min**	**Max**	**Method**	**Population**	**Reference**
66	31.6	1.9	27.8	36.7	3D facial scanner	Caucasian	[10]
45	30.5	2.2	-	-	direct anthropometry	North American White	[7]

Table S15: Morphological height of the face: (*n-gn*), male subjects. Normotypical values of the linear measurement derived from the literature, expressed in millimetres.

N	Mean	SD	Min	Max	Method	Population	Reference
-	121.3	6.8	107.7	134.9	direct anthropometry	North American White	[2]
30	121.3	5.4	110.5	132.1	direct anthropometry	Azerbaijan	[2]
30	122.9	7.6	107.7	138.1	direct anthropometry	Bulgarian	[2]
30	117.5	5.0	107.5	127.5	direct anthropometry	Chez	[2]
30	119.8	7.9	104.0	135.6	direct anthropometry	Croatian	[2]
30	116.0	8.3	99.4	132.6	direct anthropometry	German	[2]
30	120.0	7.3	105.4	136.4	direct anthropometry	Greek	[2]
30	120.0	8.1	103.8	136.2	direct anthropometry	Hungarian	[2]
30	124.7	5.7	113.3	136.2	direct anthropometry	Italian	[2]
30	117.5	6.1	105.3	129.7	direct anthropometry	Polish	[2]
30	124.5	5.7	113.1	135.9	direct anthropometry	Portuguese	[2]
30	122.9	6.3	110.3	135.5	direct anthropometry	Russian	[2]
30	115.0	7.9	99.2	130.8	direct anthropometry	Slovak	[2]
30	116.6	6.5	103.6	129.6	direct anthropometry	Slovenian	[2]
30	127.7	2.9	121.9	133.5	direct anthropometry	Turkish	[2]
516	126.8	6.5	-	-	digital stereophotogrammetry	Caucasian	[4]
50	121.3	6.8	-	-	direct anthropometry	North American White	[11]
149	121.2	13.1	-	-	photographic analysis	Turkish	[6]
109	124.7	5.7	-	-	direct anthropometry	North American White	[7]
101	125.8	6.4	-	-	direct anthropometry	Kosovo-Albanian	[14]
25	119.8	4.4	111.0	127.0	direct anthropometry	Turkish	[9]
29	121.7	4.5	113.0	133.0	direct anthropometry	Turkish	[9]
36	125.0	7.0	115.0	141.0	direct anthropometry	Turkish	[9]

Table S16: Morphological height of the face: (*n-gn*), female subjects. Normotypical values of the linear measurement derived from the literature, expressed in millimetres.

N	Mean	SD	Min	Max	Method	Population	Reference
-	111.8	5.2	101.4	122.2	direct anthropometry	North American White	[2]
30	111.5	4.0	103.5	119.5	direct anthropometry	Azerbaijan	[2]
30	111.0	4.8	101.4	120.6	direct anthropometry	Bulgarian	[2]
30	112.6	4.3	104.0	121.2	direct anthropometry	Chez	[2]
30	110.4	5.9	98.6	122.2	direct anthropometry	Croatian	[2]
30	109.5	5.0	99.5	119.5	direct anthropometry	German	[2]
30	116.4	7.1	102.2	130.6	direct anthropometry	Greek	[2]
30	112.4	5.4	101.6	123.2	direct anthropometry	Hungarian	[2]
30	113.8	5.5	102.8	124.8	direct anthropometry	Italian	[2]
30	111.6	6.7	98.2	125.0	direct anthropometry	Polish	[2]
30	118.2	7.0	104.2	132.2	direct anthropometry	Portuguese	[2]
30	114.2	6.4	101.4	127.0	direct anthropometry	Russian	[2]
30	109.3	5.4	98.5	120.1	direct anthropometry	Slovak	[2]
30	108.8	9.3	90.2	127.4	direct anthropometry	Slovenian	[2]
30	116.4	7.1	102.2	130.6	direct anthropometry	Turkish	[2]
889	118.4	6.0	-	-	digital stereophotogrammetry	Caucasian	[4]
50	111.8	5.2	-	-	direct anthropometry	North American White	[11]
281	112.4	9.5	-	-	photographic analysis	Turkish	[6]
200	111.4	4.8	-	-	direct anthropometry	North American White	[7]
34	109.7	4.8	101.0	117.0	direct anthropometry	Turkish	[9]
20	114.9	6.1	108.0	129.0	direct anthropometry	Turkish	[9]
29	116.9	5.6	107.0	128.0	direct anthropometry	Turkish	[9]
103	115.6	5.0	-	-	direct anthropometry	Kosovo-Albanian	[14]

Table S17: Height of the lower face: (*sn-gn*), male subjects. Normotypical values of the linear measurement derived from the literature, expressed in millimetres.

N	Mean	SD	Min	Max	Method	Population	Reference
-	71.9	6.0	59.9	83.9	direct anthropometry	North American White	[2]
30	69.0	4.5	60.0	78.0	direct anthropometry	Azerbaijan	[2]
30	69.5	4.8	59.9	79.1	direct anthropometry	Bulgarian	[2]
30	70.7	4.8	61.1	80.3	direct anthropometry	Chez	[2]
30	66.0	6.2	53.6	78.4	direct anthropometry	Croatian	[2]
30	67.9	7.3	53.3	82.5	direct anthropometry	German	[2]
30	65.8	7.7	50.4	81.2	direct anthropometry	Greek	[2]
30	64.2	3.9	56.4	72.0	direct anthropometry	Hungarian	[2]
30	71.4	5.2	61.0	81.8	direct anthropometry	Italian	[2]
30	68.1	3.8	60.5	75.7	direct anthropometry	Polish	[2]
30	69.6	5.7	58.2	81.0	direct anthropometry	Portuguese	[2]
30	64.5	5.3	53.9	75.1	direct anthropometry	Russian	[2]
30	63.7	6.5	50.7	76.7	direct anthropometry	Slovak	[2]
30	66.8	4.9	57.0	76.6	direct anthropometry	Slovenian	[2]
30	65.9	4.2	57.5	74.3	direct anthropometry	Turkish	[2]
536	72.8	5.2	-	-	digital stereophotogrammetry	Caucasian	[4]
100	70.5	5.6	56.6	81.4	direct anthropometry	Turkish	[5]
-	72.6	4.5	-	-	direct anthropometry	North American White	[5]
149	69.4	8.1	-	-	photographic analysis	Turkish	[6]
109	72.6	4.5	-	-	direct anthropometry	North American White	[7]
228	68.4	5.6	53.0	85.0	direct anthropometry	Turkish	[8]
25	65.2	5.4	51.0	74.0	direct anthropometry	Turkish	[9]
29	68.0	5.4	55.0	77.0	direct anthropometry	Turkish	[9]

Table S17 – Continued from previous page

N	Mean	SD	Min	Max	Method	Population	Reference
36	69.8	7.7	47.0	91.0	direct anthropometry	Turkish	[9]

Table S18: Height of the lower face: (*sn-gn*), female subjects. Normotypical values of the linear measurement derived from the literature, expressed in millimetres.

N	Mean	SD	Min	Max	Method	Population	Reference
-	65.5	4.5	56.5	74.5	direct anthropometry	North American White	[2]
30	63.6	3.4	56.8	70.4	direct anthropometry	Azerbaijan	[2]
30	61.6	4.4	52.8	70.4	direct anthropometry	Bulgarian	[2]
30	66.0	7.7	50.6	81.4	direct anthropometry	Chez	[2]
30	60.7	4.7	51.3	70.1	direct anthropometry	Croatian	[2]
30	63.3	5.2	52.9	73.7	direct anthropometry	German	[2]
30	63.3	4.3	54.7	71.9	direct anthropometry	Greek	[2]
30	56.7	3.8	49.1	64.3	direct anthropometry	Hungarian	[2]
30	64.4	5.4	53.6	75.2	direct anthropometry	Italian	[2]
30	60.5	4.4	51.7	69.3	direct anthropometry	Polish	[2]
30	62.8	4.6	53.6	72.0	direct anthropometry	Portuguese	[2]
30	61.4	7.0	47.4	75.4	direct anthropometry	Russian	[2]
30	58.6	4.3	50.0	67.2	direct anthropometry	Slovak	[2]
30	61.4	5.0	51.4	71.4	direct anthropometry	Slovenian	[2]
30	59.1	3.8	51.5	66.7	direct anthropometry	Turkish	[2]
891	66.6	5.1	-	-	digital stereophotogrammetry	Caucasian	[4]
100	63.4	5.9	45.3	76.6	direct anthropometry	Turkish	[5]
-	64.3	4.0	-	-	direct anthropometry	North American White	[5]
281	61.0	6.0	-	-	photographic analysis	Turkish	[6]
200	64.3	4.0	-	-	direct anthropometry	North American White	[7]
34	60.8	4.2	51.0	68.0	direct anthropometry	Turkish	[9]
20	63.4	4.1	58.0	73.0	direct anthropometry	Turkish	[9]
29	65.4	3.8	57.0	71.0	direct anthropometry	Turkish	[9]
272	62.6	4.6	50.0	75.0	direct anthropometry	Turkish	[8]
50	65.5	4.5	-	-	direct anthropometry	North American White	[11]

Table S19: Vermilion height of the upper lip: (*ls-sto*), male subjects. Normotypical values of the linear measurement derived from the literature, expressed in millimetres.

N	Mean	SD	Min	Max	Method	Population	Reference
534	7.7	1.7	-	-	digital stereophotogrammetry	Caucasian	[4]
50	8.9	1.5	-	-	direct anthropometry	North American White	[11]
100	6.9	1.7	4.1	11.9	direct anthropometry	Turkish	[5]
-	9.5	1.5	-	-	direct anthropometry	North American White	[5]
149	5.3	1.3	-	-	photographic analysis	Turkish	[6]
109	8.0	1.4	-	-	direct anthropometry	North American White	[7]

Table S20: Vermilion height of the upper lip: (*ls-sto*), female subjects. Normotypical values of the linear measurement derived from the literature, expressed in millimetres.

N	Mean	SD	Min	Max	Method	Population	Reference
906	7.6	1.4	-	-	digital stereophotogrammetry	Caucasian	[4]
400	6.5	1.4	-	-	photogrammetry	Caucasian	[13]
100	7.6	1.3	3.7	10.7	direct anthropometry	Turkish	[5]
-	8.6	1.6	-	-	direct anthropometry	North American White	[5]
281	5.1	1.1	-	-	photographic analysis	Turkish	[6]
200	8.7	1.3	-	-	direct anthropometry	North American White	[7]

Table S21: Vermilion height of the lower lip: (*li-sto*), male subjects. Normotypical values of the linear measurement derived from the literature, expressed in millimetres.

N	Mean	SD	Min	Max	Method	Population	Reference
535	9.0	2.3	-	-	digital stereophotogrammetry	Caucasian	[4]
50	10.4	1.9	-	-	direct anthropometry	North American White	[11]
100	9.2	1.8	4.5	14.2	direct anthropometry	Turkish	[5]
-	11.0	1.2	-	-	direct anthropometry	North American White	[5]
149	9.1	2.4	-	-	photographic analysis	Turkish	[6]
109	9.3	1.6	-	-	direct anthropometry	North American White	[7]

Table S22: Vermilion height of the lower lip: (*li-sto*), female subjects. Normotypical values of the linear measurement derived from the literature, expressed in millimetres.

N	Mean	SD	Min	Max	Method	Population	Reference
902	8.9	1.9	-	-	digital stereophotogrammetry	Caucasian	[4]
50	9.7	1.6	-	-	direct anthropometry	North American White	[11]
400	11.6	1.5	-	-	photogrammetry	Caucasian	[13]
100	9.7	1.4	5.9	13.1	direct anthropometry	Turkish	[5]
-	10.0	1.5	-	-	direct anthropometry	North American White	[5]
281	9.0	1.8	-	-	photographic analysis	Turkish	[6]
200	9.4	1.5	-	-	direct anthropometry	North American White	[7]

Table S23: Height of the upper lip: (*sn-sto*), male subjects. Normotypical values of the linear measurement derived from the literature, expressed in millimetres.

N	Mean	SD	Min	Max	Method	Population	Reference
271	21.6	-	-	-	photogrammetry	Caucasian	[3]
535	22.3	2.7	-	-	digital stereophotogrammetry	Caucasian	[4]
50	21.8	2.0	-	-	direct anthropometry	North American White	[11]
149	21.6	3.3	-	-	photographic analysis	Turkish	[6]
109	22.3	2.1	-	-	direct anthropometry	North American White	[7]
25	20.5	1.9	16.0	24.0	direct anthropometry	Turkish	[9]
29	21.7	2.9	16.0	29.0	direct anthropometry	Turkish	[9]
36	21.4	3.7	14.0	31.0	direct anthropometry	Turkish	[9]

Table S24: Height of the upper lip: (*sn-sto*), female subjects. Normotypical values of the linear measurement derived from the literature, expressed in millimetres.

N	Mean	SD	Min	Max	Method	Population	Reference
830	19.7	-	-	-	photogrammetry	Caucasian	[3]
906	20.3	2.6	-	-	digital stereophotogrammetry	Caucasian	[4]
50	19.6	2.4	-	-	direct anthropometry	North American White	[11]
400	18.7	2.0	-	-	photogrammetry	Caucasian	[13]
281	19.3	2.9	-	-	photographic analysis	Turkish	[6]
200	20.1	2.0	-	-	direct anthropometry	North American White	[7]
34	19.0	2.3	15.0	25.0	direct anthropometry	Turkish	[9]
20	19.4	2.0	17.0	23.0	direct anthropometry	Turkish	[9]
29	20.6	2.1	18.0	27.0	direct anthropometry	Turkish	[9]

Table S25: Height of forehead II: (*tr-n*), male subjects. Normotypical values of the linear measurement derived from the literature, expressed in millimetres.

N	Mean	SD	Min	Max	Method	Population	Reference
40	61.0	6.9	-	-	infrared camera	Caucasian	[1]
-	70.1	6.4	57.3	82.9	direct anthropometry	North American White	[2]
30	64.7	7.1	50.5	78.9	direct anthropometry	Azerbaijan	[2]
30	66.4	7.2	52.0	80.8	direct anthropometry	Bulgarian	[2]
30	69.6	3.1	63.4	75.8	direct anthropometry	Chez	[2]
30	60.3	5.9	48.5	72.1	direct anthropometry	Croatian	[2]
30	69.9	8.3	53.3	86.5	direct anthropometry	German	[2]
30	64.3	8.6	47.2	81.5	direct anthropometry	Greek	[2]
30	72.8	8.0	56.8	88.8	direct anthropometry	Hungarian	[2]
30	63.1	7.8	47.5	78.7	direct anthropometry	Italian	[2]
30	68.8	5.3	58.2	79.4	direct anthropometry	Polish	[2]
30	67.6	5.9	55.8	79.4	direct anthropometry	Portuguese	[2]
30	75.1	7.8	59.5	90.7	direct anthropometry	Russian	[2]
30	68.8	7.5	53.8	83.8	direct anthropometry	Slovak	[2]
30	69.3	9.3	50.7	87.9	direct anthropometry	Slovenian	[2]
30	61.9	6.1	49.7	74.1	direct anthropometry	Turkish	[2]
100	68.8	9.6	42.7	95.8	direct anthropometry	Turkish	[5]
149	64.7	9.7	-	-	photographic analysis	Turkish	[6]
109	67.1	7.5	-	-	direct anthropometry	North American White	[7]
228	65.3	9.0	41.0	97.0	direct anthropometry	Turkish	[8]
25	64.6	8.3	50.0	80.0	direct anthropometry	Turkish	[9]
29	60.5	9.3	47.0	77.0	direct anthropometry	Turkish	[9]
36	60.0	8.6	40.0	79.0	direct anthropometry	Turkish	[9]

Table S26: Height of forehead II: (*tr-n*), female subjects. Normotypical values of the linear measurement derived from the literature, expressed in millimetres.

N	Mean	SD	Min	Max	Method	Population	Reference
40	57.3	8.6	-	-	infrared camera	Caucasian	[1]
66	63.7	6.2	48.5	79.2	3D facial scanner	Caucasian	[10]
-	67.1	-	-	-	direct anthropometry	North American White	[15]
-	63.3	6.5	50.5	76.5	direct anthropometry	North American White	[2]
30	60.6	3.9	52.8	68.4	direct anthropometry	Azerbaijan	[2]
30	63.4	6.7	50.0	76.8	direct anthropometry	Bulgarian	[2]

Table S26 – Continued from previous page

N	Mean	SD	Min	Max	Method	Population	Reference
30	67.3	6.3	54.7	79.9	direct anthropometry	Chez	[2]
30	62.2	6.1	50.0	74.4	direct anthropometry	Croatian	[2]
30	66.3	6.2	53.9	78.7	direct anthropometry	German	[2]
30	63.1	4.9	53.3	72.9	direct anthropometry	Greek	[2]
30	69.2	6.0	57.2	81.2	direct anthropometry	Hungarian	[2]
30	59.4	6.8	45.8	73.0	direct anthropometry	Italian	[2]
30	63.7	6.4	50.9	76.5	direct anthropometry	Polish	[2]
30	62.2	5.4	51.4	73.0	direct anthropometry	Portuguese	[2]
30	70.7	6.7	57.3	84.1	direct anthropometry	Russian	[2]
30	66.2	6.8	52.6	79.8	direct anthropometry	Slovak	[2]
30	65.3	6.8	51.7	78.9	direct anthropometry	Slovenian	[2]
30	60.7	7.0	46.7	74.7	direct anthropometry	Turkish	[2]
490	64.1	-	-	-	photogrammetry	Caucasian	[3]
100	66.9	8.1	46.1	93.6	direct anthropometry	Turkish	[5]
281	63.3	6.9	-	-	photographic analysis	Turkish	[6]
200	63.0	6.0	-	-	direct anthropometry	North American White	[7]
34	66.6	7.9	51.0	80.0	direct anthropometry	Turkish	[9]
20	65.6	9.0	51.0	82.7	direct anthropometry	Turkish	[9]
29	64.7	7.7	50.0	80.0	direct anthropometry	Turkish	[9]
272	61.6	7.5	40.0	82.0	direct anthropometry	Turkish	[8]

Table S27: Width of the mandible: (Go_r-Go_l), male subjects. Normotypical values of the linear measurement derived from the literature, expressed in millimetres.

N	Mean	SD	Min	Max	Method	Population	Reference
40	111.9	10.3	-	-	infrared camera	Caucasian	[1]
-	97.1	5.8	85.5	108.7	direct anthropometry	North American White	[2]
30	106.3	4.1	98.1	114.5	direct anthropometry	Azerbaijan	[2]
30	108.8	9.1	90.6	127.0	direct anthropometry	Bulgarian	[2]
30	109.5	3.8	101.9	117.1	direct anthropometry	Chez	[2]
30	102.1	6.3	89.5	114.7	direct anthropometry	Croatian	[2]
30	97.6	6	85.6	109.6	direct anthropometry	German	[2]
30	108.7	9.1	90.5	126.9	direct anthropometry	Greek	[2]
30	106.6	7	92.6	120.6	direct anthropometry	Hungarian	[2]
30	116.3	6.9	102.5	130.1	direct anthropometry	Italian	[2]
30	98.8	6.5	85.8	111.8	direct anthropometry	Polish	[2]
30	87.6	3.8	80.0	95.2	direct anthropometry	Portuguese	[2]
30	103.8	5.4	93.0	114.6	direct anthropometry	Russian	[2]
30	118.6	8.5	101.6	135.6	direct anthropometry	Slovak	[2]
30	106.3	7	92.3	120.3	direct anthropometry	Slovenian	[2]
30	109.5	4.3	100.9	118.1	direct anthropometry	Turkish	[2]
514	104.2	7.2	-	-	direct anthropometry	Caucasian	[4]
50	97.1	5.8	-	-	direct anthropometry	North American White	[11]
100	111.5	9.2	90.2	141.6	direct anthropometry	Turkish	[5]
149	116.3	12.6	-	-	photographic analysis	Turkish	[6]
109	105.6	6.7	-	-	direct anthropometry	North American White	[7]
25	117.5	7.2	103.0	133.0	direct anthropometry	Turkish	[9]
29	115.8	9.2	102.0	142.0	direct anthropometry	Turkish	[9]
36	116.5	8.6	97.0	131.0	direct anthropometry	Turkish	[9]

Table S28: Width of the mandible: (Go_r-Go_l), female subjects. Normotypical values of the linear measurement derived from the literature, expressed in millimetres.

N	Mean	SD	Min	Max	Method	Population	Reference
40	104.7	7.6	-	-	infrared camera	Caucasian	[1]
-	91.1	5.9	79.3	102.9	direct anthropometry	North American White	[2]
30	102.9	5.9	91.1	114.7	direct anthropometry	Azerbaijan	[2]
30	98.7	4.8	89.1	108.3	direct anthropometry	Bulgarian	[2]
30	107.1	3.4	100.3	113.9	direct anthropometry	Chez	[2]
30	94.6	5.5	83.6	105.6	direct anthropometry	Croatian	[2]
30	91.5	5	81.5	101.5	direct anthropometry	German	[2]
30	99.2	4.5	90.2	108.2	direct anthropometry	Greek	[2]
30	95.0	5.7	83.6	106.4	direct anthropometry	Hungarian	[2]
30	104.9	4.8	95.3	114.5	direct anthropometry	Italian	[2]
30	93.9	4.2	85.5	102.3	direct anthropometry	Polish	[2]
30	84.3	3.8	76.7	91.9	direct anthropometry	Portuguese	[2]
30	98.6	4.1	90.4	106.8	direct anthropometry	Russian	[2]
30	105.4	4.4	96.6	114.2	direct anthropometry	Slovak	[2]
30	100.7	5.4	89.9	111.5	direct anthropometry	Slovenian	[2]
30	100.0	7.8	84.4	115.6	direct anthropometry	Turkish	[2]
383	105.5	-	-	-	photogrammetry	Caucasian	[3]
835	96.7	7.0	-	-	direct anthropometry	Caucasian	[4]
50	91.1	5.9	-	-	direct anthropometry	North American White	[11]
400	116.8	6.8	-	-	photogrammetry	Caucasian	[13]
100	107.4	8.7	86.0	141.6	direct anthropometry	Turkish	[5]
281	110.2	16.5	-	-	photographic analysis	Turkish	[6]
200	94.5	5.0	-	-	direct anthropometry	North American White	[7]
34	110.0	7.3	91.0	132.0	direct anthropometry	Turkish	[9]
20	107.3	7.0	97.0	120.0	direct anthropometry	Turkish	[9]
29	113.2	8.0	94.0	128.0	direct anthropometry	Turkish	[9]

Table S29: Depth in the maxillary region (right/left): (sn-t), male subjects. Normotypical values of the linear measurement derived from the literature, expressed in millimetres.

N	Mean	SD	Min	Max	Method	Population	Reference
532	133.0	5.0	-	-	digital stereophotogrammetry	Caucasian	[4]
100	131.3	4.8	110.6	143.4	direct anthropometry	Turkish	[5]
109	133.0	4.6	-	-	direct anthropometry	North American White	[7]
530	132.2	5.1	-	-	digital stereophotogrammetry	Caucasian	[4]
109	131.8	4.3	-	-	direct anthropometry	North American White	[7]

Table S30: Depth in the maxillary region (right/left): (sn-t), female subjects. Normotypical values of the linear measurement derived from the literature, expressed in millimetres.

N	Mean	SD	Min	Max	Method	Population	Reference
66	113.7	6.5	99.3	131.2	3D facial scanner	Caucasian	[10]
890	123.8	4.5	-	-	digital stereophotogrammetry	Caucasian	[4]
100	122.2	4.6	110.8	134.5	direct anthropometry	Turkish	[5]
200	120.5	3.8	-	-	direct anthropometry	North American White	[7]
66	113.8	3.7	103.9	122.4	3D facial scanner	Caucasian	[10]
888	123.6	4.6	-	-	digital stereophotogrammetry	Caucasian	[4]
200	119.3	4.0	-	-	direct anthropometry	North American White	[7]

Table S31: Depth of the upper third of the face (right/left): (n-t), male subjects. Normotypical values of the linear measurement derived from the literature, expressed in millimetres.

N	Mean	SD	Min	Max	Method	Population	Reference
532	126.7	4.9	-	-	digital stereophotogrammetry	Caucasian	[4]
100	126.7	5.2	110.1	137.7	direct anthropometry	Turkish	[5]
109	126.6	4.2	-	-	direct anthropometry	North American White	[7]
530	126.3	5.0	-	-	digital stereophotogrammetry	Caucasian	[4]
109	126.2	4.3	-	-	direct anthropometry	North American White	[7]

Table S32: Depth of the upper third of the face (right/left): (n-t), female subjects. Normotypical values of the linear measurement derived from the literature, expressed in millimetres.

N	Mean	SD	Min	Max	Method	Population	Reference
66	110.0	4.0	101.2	117.9	3D facial scanner	Caucasian	[10]
890	118.8	4.4	-	-	digital stereophotogrammetry	Caucasian	[4]
100	118.2	4.3	106.9	129.1	direct anthropometry	Turkish	[5]
200	116.5	4.5	-	-	direct anthropometry	North American White	[7]
66	109.8	4.0	100.0	119.7	3D facial scanner	Caucasian	[10]
888	118.7	4.5	-	-	digital stereophotogrammetry	Caucasian	[4]
200	116.0	4.5	-	-	direct anthropometry	North American White	[7]

Table S33: Length of the eye fissure (right/left): (ex-en), male subjects. Normotypical values of the linear measurement derived from the literature, expressed in millimetres.

N	Mean	SD	Min	Max	Method	Population	Reference
-	31.2	1.3	28.6	33.8	direct anthropometry	North American White	[2]
30	34.4	1.7	31.0	37.8	direct anthropometry	Azerbaijan	[2]
30	31.3	1.6	28.1	34.5	direct anthropometry	Bulgarian	[2]
30	29.4	1.8	25.8	33.0	direct anthropometry	Chez	[2]
30	30.5	1.8	26.9	34.1	direct anthropometry	Croatian	[2]
30	31.9	2.7	26.5	37.3	direct anthropometry	German	[2]
30	34.8	4	26.8	42.8	direct anthropometry	Greek	[2]
30	37.7	3	31.7	43.7	direct anthropometry	Hungarian	[2]
30	33.9	2.2	29.5	38.3	direct anthropometry	Italian	[2]
30	34.0	2	30.0	38.0	direct anthropometry	Polish	[2]
30	38.1	2.4	33.3	42.9	direct anthropometry	Portuguese	[2]
30	34.6	2.5	29.6	39.6	direct anthropometry	Russian	[2]
30	34.9	1.6	31.7	38.1	direct anthropometry	Slovak	[2]
30	36.4	2.9	30.6	42.2	direct anthropometry	Slovenian	[2]
30	30.6	1.2	28.2	33.0	direct anthropometry	Turkish	[2]
528	29.0	1.8	-	-	digital stereophotogrammetry	Caucasian	[4]
100	33.9	2.5	26.9	38.6	direct anthropometry	Turkish	[5]
109	31.3	1.2	-	-	direct anthropometry	North American White	[7]
528	28.7	1.9	-	-	digital stereophotogrammetry	Caucasian	[4]

Table S33 – Continued from previous page

N	Mean	SD	Min	Max	Method	Population	Reference
100	33.9	2.3	27.0	38.4	direct anthropometry	Turkish	[5]
-	31.3	1.4	-	-	direct anthropometry	North American White	[5]
228	32.6	2.5	28.0	50.0	direct anthropometry	Turkish	[8]
25	35.8	2.3	31.0	41.0	direct anthropometry	Turkish	[9]
29	34.5	2.8	29.0	41.0	direct anthropometry	Turkish	[9]
36	34.6	3.2	26.0	43.0	direct anthropometry	Turkish	[9]
25	35.2	2.5	31.0	40.0	direct anthropometry	Turkish	[9]
29	34.2	2.9	28.0	41.0	direct anthropometry	Turkish	[9]
36	34.0	3.3	23.0	42.0	direct anthropometry	Turkish	[9]

Table S34: length of the eye fissure (right/left): (*ex-en*), female subjects. Normotypical values of the linear measurement derived from the literature, expressed in millimetres.

N	Mean	SD	Min	Max	Method	Population	Reference
66	27.8	1.5	23.6	31.2	3D facial scanner	Caucasian	[10]
-	30.7	1.8	27.1	34.3	direct anthropometry	North American White	[2]
30	33.8	1.6	30.6	37.0	direct anthropometry	Azerbaijan	[2]
30	30.4	1.3	27.8	33.0	direct anthropometry	Bulgarian	[2]
30	28.2	2.1	24.0	32.4	direct anthropometry	Chez	[2]
30	38.3	1.9	34.5	42.1	direct anthropometry	Croatian	[2]
30	31.8	1.9	28.0	35.6	direct anthropometry	German	[2]
30	32.0	2.8	26.4	37.6	direct anthropometry	Greek	[2]
30	34.9	2.4	30.1	39.7	direct anthropometry	Hungarian	[2]
30	32.7	2.1	28.5	36.9	direct anthropometry	Italian	[2]
30	32.8	1.8	29.2	36.4	direct anthropometry	Polish	[2]
30	35.9	2.6	30.7	41.1	direct anthropometry	Portuguese	[2]
30	34.5	1.9	30.7	38.3	direct anthropometry	Russian	[2]
30	32.3	2	28.3	36.3	direct anthropometry	Slovak	[2]
30	33.2	2.2	28.8	37.6	direct anthropometry	Slovenian	[2]
30	29.8	1.6	26.6	33.0	direct anthropometry	Turkish	[2]
896	28.1	1.9	-	-	digital stereophotogrammetry	Caucasian	[4]
100	33.5	1.9	28.2	38.1	direct anthropometry	Turkish	[5]
200	30.7	1.2	-	-	direct anthropometry	North American White	[7]
66	27.5	1.3	25.0	31.1	3D facial scanner	Caucasian	[10]
897	27.8	2.0	-	-	digital stereophotogrammetry	Caucasian	[4]
100	33.4	1.8	28.5	38.2	direct anthropometry	Turkish	[5]
-	30.7	1.8	-	-	direct anthropometry	North American White	[5]
34	33.5	2.6	24.0	37.0	direct anthropometry	Turkish	[9]
20	34.2	2.3	31.0	39.0	direct anthropometry	Turkish	[9]
29	34.1	2.0	31.0	38.0	direct anthropometry	Turkish	[9]
34	33.0	2.7	22.0	37.0	direct anthropometry	Turkish	[9]
20	34.5	2.5	30.0	40.0	direct anthropometry	Turkish	[9]
29	33.6	1.7	30.0	55.0	direct anthropometry	Turkish	[9]
272	31.0	2.4	26.0	40.0	direct anthropometry	Turkish	[8]

Table S35: Length of the nasal bridge: (*n-prn*), male subjects. Normotypical values of the linear measurement derived from the literature, expressed in millimetres.

N	Mean	SD	Min	Max	Method	Population	Reference
535	49.8	3.9	-	-	digital stereophotogrammetry	Caucasian	[4]
50	53.0	3.5	-	-	direct anthropometry	North American White	[11]
40	49.0	4.2	40.6	57.4	direct anthropometry	North American White	[16]
149	40.3	7.1	-	-	photographic analysis	Turkish	[6]
109	50.0	3.6	-	-	direct anthropometry	North American White	[7]

Table S36: Length of the nasal bridge : (*n-prn*), female subjects. Normotypical values of the linear measurement derived from the literature, expressed in millimetres.

N	Mean	SD	Min	Max	Method	Population	Reference
66	43.4	2.5	36.2	48.9	3D facial scanner	Caucasian	[10]
907	47.3	3.6	-	-	digital stereophotogrammetry	Caucasian	[4]
209	42.2	-	-	-	photogrammetry	Caucasian	[3]
50	48.9	2.6	-	-	direct anthropometry	North American White	[11]
40	45.4	3.9	37.6	53.2	direct anthropometry	North American White	[16]
281	40.1	5.6	-	-	photographic analysis	Turkish	[6]
200	44.7	3.4	-	-	direct anthropometry	North American White	[7]

Table S37: Nasal tip protrusion: (*sn-prn*), male subjects. Normotypical values of the linear measurement derived from the literature, expressed in millimetres.

N	Mean	SD	Min	Max	Method	Population	Reference
532	21.1	2.1	-	-	digital stereophotogrammetry	Caucasian	[4]

Table S37 – Continued from previous page

N	Mean	SD	Min	Max	Method	Population	Reference
100	20.4	3.2	13.4	27.9	direct anthropometry	Turkish	[5]
-	20.6	2.2	-	-	direct anthropometry	North American White	[5]
109	19.5	1.9	-	-	direct anthropometry	North American White	[7]

Table S38: Nasal tip protrusion: (*sn-prn*), female subjects. Normotypical values of the linear measurement derived from the literature, expressed in millimetres.

N	Mean	SD	Min	Max	Method	Population	Reference
66	18.6	1.8	14.3	22.3	3D facial scanner	Caucasian	[10]
904	20.0	2.0	-	-	digital stereophotogrammetry	Caucasian	[4]
100	19.8	2.4	13.5	25.5	direct anthropometry	Turkish	[5]
-	19.4	1.7	-	-	direct anthropometry	North American White	[5]
200	19.7	1.6	-	-	direct anthropometry	North American White	[7]

Table S39: Height of the coutaneous upper lip: (*sn-ls*), male subjects. Normotypical values of the linear measurement derived from the literature, expressed in millimetres.

N	Mean	SD	Min	Max	Method	Population	Reference
109	15.9	1.9	-	-	direct anthropometry	North American White	[7]
534	15.9	2.6	-	-	digital stereophotogrammetry	Caucasian	[4]

Table S40: Height of the coutaneous upper lip: (*sn-ls*), female subjects. Normotypical values of the linear measurement derived from the literature, expressed in millimetres.

N	Mean	SD	Min	Max	Method	Population	Reference
66	12.2	1.5	9.1	16.2	3D facial scanner	Caucasian	[10]
400	12.3	1.8	-	-	photogrammetry	Caucasian	[13]
199	13.8	4.6	-	-	direct anthropometry	North American White	[7]
907	13.9	2.5	-	-	digital stereophotogrammetry	Caucasian	[4]

Table S41: Width of the face: (zy_r-zy_l), male subjects. Normotypical values of the linear measurement derived from the literature, expressed in millimetres.

N	Mean	SD	Min	Max	Method	Population	Reference
-	137.1	4.3	128.5	145.7	direct anthropometry	North American White	[2]
30	147.5	5.4	136.7	158.3	direct anthropometry	Azerbaijan	[2]
30	139.5	5.6	128.3	150.7	direct anthropometry	Bulgarian	[2]
30	134.9	13.3	108.3	161.5	direct anthropometry	Chez	[2]
30	140.7	6	128.7	152.7	direct anthropometry	Croatian	[2]
30	133.2	7.5	118.2	148.2	direct anthropometry	German	[2]
30	128.6	11.4	105.8	151.4	direct anthropometry	Greek	[2]
30	142.1	5.3	131.5	152.7	direct anthropometry	Hungarian	[2]
30	143.2	5.9	131.4	155.0	direct anthropometry	Italian	[2]
30	142.6	4.7	133.2	152.0	direct anthropometry	Polish	[2]
30	125.1	5.4	114.3	135.9	direct anthropometry	Portuguese	[2]
30	141.2	4.4	132.4	150.0	direct anthropometry	Russian	[2]
30	134.7	5.5	123.7	145.7	direct anthropometry	Slovak	[2]
30	136.2	5.8	124.6	147.8	direct anthropometry	Slovenian	[2]
30	140.4	8.2	124.0	156.8	direct anthropometry	Turkish	[2]
149	130.7	-	-	-	photogrammetry	Caucasian	[3]
502	137.2	6.1	-	-	direct anthropometry	Caucasian	[4]
50	137.1	4.3	-	-	direct anthropometry	North American White	[11]
100	129.1	7.1	115.1	145.4	direct anthropometry	Turkish	[5]
-	139.1	6.3	-	-	direct anthropometry	North American White	[5]
149	123.1	13.0	-	-	photographic analysis	Turkish	[6]
109	139.1	5.3	-	-	direct anthropometry	North American White	[7]
25	127.1	8.6	121.0	145.0	direct anthropometry	Turkish	[9]
29	120.9	6.1	109.0	132.0	direct anthropometry	Turkish	[9]
36	113.8	8.9	98.0	129.0	direct anthropometry	Turkish	[9]
101	131.9	6.7	-	-	direct anthropometry	Kosovo-Albanian	[14]

Table S42: Width of the face: (zy_r-zy_l), female subjects. Normotypical values of the linear measurement derived from the literature, expressed in millimetres.

N	Mean	SD	Min	Max	Method	Population	Reference
-	129.9	5.3	119.3	140.5	direct anthropometry	North American White	[2]
30	138.7	5.2	128.3	149.1	direct anthropometry	Azerbaijan	[2]
30	130.9	4.4	122.1	139.7	direct anthropometry	Bulgarian	[2]
30	126.4	14.4	97.6	155.2	direct anthropometry	Chez	[2]
30	133.2	6.8	119.6	146.8	direct anthropometry	Croatian	[2]
30	133.2	7.5	118.2	148.2	direct anthropometry	German	[2]

Table S42 – Continued from previous page

N	Mean	SD	Min	Max	Method	Population	Reference
30	123.4	9.2	105.0	141.8	direct anthropometry	German	[2]
30	132.2	4.8	122.6	141.8	direct anthropometry	Greek	[2]
30	131.3	3.5	124.3	138.3	direct anthropometry	Hungarian	[2]
30	133.3	4.1	125.1	141.5	direct anthropometry	Italian	[2]
30	135.5	5.5	124.5	146.5	direct anthropometry	Polish	[2]
30	120.4	5.4	109.6	131.2	direct anthropometry	Portuguese	[2]
30	132.3	4.8	122.7	141.9	direct anthropometry	Russian	[2]
30	125.0	5.7	113.6	136.4	direct anthropometry	Slovak	[2]
30	129.5	5.2	119.1	139.9	direct anthropometry	Slovenian	[2]
30	134.5	4.3	125.9	143.1	direct anthropometry	Turkish	[2]
383	123.3	-	-	-	photogrammetry	Caucasian	[3]
799	129.8	5.2	-	-	direct anthropometry	Caucasian	[4]
50	129.9	5.3	-	-	direct anthropometry	North American White	[11]
100	127.2	6.5	111.2	141.6	direct anthropometry	Turkish	[5]
-	131.1	5.3	-	-	direct anthropometry	North American White	[5]
281	116.8	11.4	-	-	photographic analysis	Turkish	[6]
200	130.0	4.6	-	-	direct anthropometry	North American White	[7]
34	120.1	6.6	109.0	141.0	direct anthropometry	Turkish	[9]
20	114.2	6.0	105.0	126.0	direct anthropometry	Turkish	[9]
29	105.2	6.5	96.0	122.0	direct anthropometry	Turkish	[9]
103	123.9	5.6	-	-	direct anthropometry	Kosovo-Albanian	[14]

Table S43: Lower half of the cranial height (right/left): (*en-gn*), male subjects. Normotypical values of the linear measurement derived from the literature, expressed in millimetres.

N	Mean	SD	Min	Max	Method	Population	Reference
100	113.2	6.5	96.3	129.7	direct anthropometry	Turkish	[5]
109	117.7	5.6	-	-	direct anthropometry	North American White	[7]
228	103.7	6.3	61.0	118.0	direct anthropometry	Turkish	[8]

Table S44: Lower half of the cranial height (right/left): (*en-gn*), female subjects. Normotypical values of the linear measurement derived from the literature, expressed in millimetres.

N	Mean	SD	Min	Max	Method	Population	Reference
100	104.0	5.4	93.7	119.8	direct anthropometry	Turkish	[5]
200	102.7	5.1	-	-	direct anthropometry	North American White	[7]
272	94.2	4.9	81.0	107.0	direct anthropometry	Turkish	[8]

Table S45: Height of forehead I: (*tr-g*), male subjects. Normotypical values of the linear measurement derived from the literature, expressed in millimetres.

N	Mean	SD	Min	Max	Method	Population	Reference
271	55.1	-	-	-	photogrammetry	Caucasian	[3]
100	52.7	9.6	29.8	84.7	direct anthropometry	Turkish	[5]
149	53.0	8.6	-	-	photographic analysis	Turkish	[6]
109	57.0	7.4	-	-	direct anthropometry	North American White	[7]
228	55.6	8.6	38.0	86.0	direct anthropometry	Turkish	[8]
25	56.6	10.5	42.0	82.0	direct anthropometry	Turkish	[9]
29	50.3	8.8	37.0	69.0	direct anthropometry	Turkish	[9]
36	50.3	6.4	38.0	65.0	direct anthropometry	Turkish	[9]

Table S46: Height of forehead I: (*tr-g*), female subjects. Normotypical values of the linear measurement derived from the literature, expressed in millimetres.

N	Mean	SD	Min	Max	Method	Population	Reference
730	52.8	-	-	-	photogrammetry	Caucasian	[3]
100	51.3	7.6	36.1	69.2	direct anthropometry	Turkish	[5]
281	53.5	6.7	-	-	photographic analysis	Turkish	[6]
200	52.7	6.0	-	-	direct anthropometry	North American White	[7]
34	57.3	8.3	37.0	71.0	direct anthropometry	Turkish	[9]
20	55.9	8.6	42.0	74.4	direct anthropometry	Turkish	[9]
29	54.7	7.1	38.0	69.0	direct anthropometry	Turkish	[9]
272	52.1	7.0	34.0	72.0	direct anthropometry	Turkish	[8]

Table S47: Distance between the glabella and the subnasale: (*g-sn*), male subjects. Normotypical values of the linear measurement derived from the literature, expressed in millimetres.

N	Mean	SD	Min	Max	Method	Population	Reference
271	65.5	-	-	-	photogrammetry	Caucasian	[3]
100	73.5	5.1	61.4	87.6	direct anthropometry	Turkish	[5]
149	63.7	7.5	-	-	photographic analysis	Turkish	[6]
109	67.2	4.9	-	-	direct anthropometry	North American White	[7]

Table S47 – Continued from previous page

N	Mean	SD	Min	Max	Method	Population	Reference
228	50.3	4.0	40.0	59.0	direct anthropometry	Turkish	[8]

Table S48: Distance between the glabella and the subnasale: (g-sn), female subjects. Normotypical values of the linear measurement derived from the literature, expressed in millimetres.

N	Mean	SD	Min	Max	Method	Population	Reference
730	63.7	-	-	-	photogrammetry	Caucasian	[3]
100	69.2	4.5	58.0	79.8	direct anthropometry	Turkish	[5]
281	61.4	5.4	-	-	photographic analysis	Turkish	[6]
200	63.1	4.4	-	-	direct anthropometry	North American White	[7]
272	47.8	3.8	36.0	57.0	direct anthropometry	Turkish	[8]

Table S49: Width of the nose: (al_r-al_l), male subjects. Normotypical values of the linear measurement derived from the literature, expressed in millimetres.

N	Mean	SD	Min	Max	Method	Population	Reference
-	34.7	2.6	29.5	39.9	direct anthropometry	North American White	[2]
30	35.7	2.4	30.9	40.5	direct anthropometry	Azerbaijan	[2]
30	36.0	3.3	29.4	42.6	direct anthropometry	Bulgarian	[2]
30	36.2	2.5	31.2	41.2	direct anthropometry	Chez	[2]
30	36.5	2.7	31.1	41.9	direct anthropometry	Croatian	[2]
30	34.0	2.2	29.6	38.4	direct anthropometry	German	[2]
30	35.7	2.9	29.9	41.5	direct anthropometry	Greek	[2]
30	37.3	2.5	32.3	42.3	direct anthropometry	Hungarian	[2]
30	32.1	2.1	27.9	36.3	direct anthropometry	Italian	[2]
30	35.2	2	31.2	39.2	direct anthropometry	Polish	[2]
30	36.6	2.9	30.8	42.4	direct anthropometry	Portuguese	[2]
30	35.8	2.3	31.2	40.4	direct anthropometry	Russian	[2]
30	33.6	2.2	29.2	38.0	direct anthropometry	Slovak	[2]
30	35.9	2.9	30.1	41.7	direct anthropometry	Slovenian	[2]
30	36.8	2.3	32.2	41.4	direct anthropometry	Turkish	[2]
169	37.9	-	-	-	photogrammetry	Caucasian	[3]
533	36.1	2.5	-	-	digital stereophotogrammetry	Caucasian	[4]
50	34.7	2.6	-	-	direct anthropometry	North American White	[11]
100	35.1	2.9	28.9	40.9	direct anthropometry	Turkish	[5]
-	34.8	2.7	-	-	direct anthropometry	North American White	[5]
149	38.4	4.4	-	-	photographic analysis	Turkish	[6]
109	34.9	2.1	-	-	direct anthropometry	North American White	[7]
228	35.9	2.9	27.0	47.0	direct anthropometry	Turkish	[8]
25	36.9	3.3	26.0	42.0	direct anthropometry	Turkish	[9]
29	39.1	4.9	34.0	58.0	direct anthropometry	Turkish	[9]
36	35.4	2.9	31.0	42.0	direct anthropometry	Turkish	[9]

Table S50: Width of the nose: (al_r-al_l), female subjects. Normotypical values of the linear measurement derived from the literature, expressed in millimetres.

N	Mean	SD	Min	Max	Method	Population	Reference
-	31.4	1.9	27.6	35.2	direct anthropometry	North American White	[2]
30	33.8	2.7	28.4	39.2	direct anthropometry	Azerbaijan	[2]
30	33.0	4.7	23.6	42.4	direct anthropometry	Bulgarian	[2]
30	33.8	2.5	28.8	38.8	direct anthropometry	Chez	[2]
30	32.9	2	28.9	36.9	direct anthropometry	Croatian	[2]
30	31.0	2.7	25.6	36.4	direct anthropometry	German	[2]
30	32.4	2.2	28.0	36.8	direct anthropometry	Greek	[2]
30	33.5	1.9	29.7	37.3	direct anthropometry	Hungarian	[2]
30	29.5	2.4	24.7	34.3	direct anthropometry	Italian	[2]
30	32.6	2.7	27.2	38.0	direct anthropometry	Polish	[2]
30	31.9	2.4	27.1	36.7	direct anthropometry	Portuguese	[2]
30	33.2	2.2	28.8	37.6	direct anthropometry	Russian	[2]
30	30.6	1.8	27.0	34.2	direct anthropometry	Slovak	[2]
30	33.1	2.5	28.1	38.1	direct anthropometry	Slovenian	[2]
30	32.9	2.1	28.7	37.1	direct anthropometry	Turkish	[2]
510	35.1	-	-	-	photogrammetry	Caucasian	[3]
907	32.4	2.3	-	-	digital stereophotogrammetry	Caucasian	[4]
50	31.4	1.9	-	-	direct anthropometry	North American White	[11]
100	32.3	2.7	24.2	39.6	direct anthropometry	Turkish	[5]
-	31.9	1.0	-	-	direct anthropometry	North American White	[5]
281	34.8	2.9	-	-	photographic analysis	Turkish	[6]
200	31.4	2.0	-	-	direct anthropometry	North American White	[7]
34	32.4	2.7	23.0	37.0	direct anthropometry	Turkish	[9]
20	33.4	2.6	30.0	40.0	direct anthropometry	Turkish	[9]
29	32.5	2.9	26.0	36.0	direct anthropometry	Turkish	[9]
272	32.3	3.1	27.0	54.0	direct anthropometry	Turkish	[8]

Table S51: Width of the nasal root: (mf_r-mf_l), male subjects. Normotypical values of the linear measurement derived from the literature, expressed in millimetres.

N	Mean	SD	Min	Max	Method	Population	Reference
149	20.5	2.9	-	-	photographic analysis	Turkish	[6]
109	19.6	1.9	-	-	direct anthropometry	North American White	[7]

Table S52: Width of the nasal root: (mf_r-mf_l), female subjects. Normotypical values of the linear measurement derived from the literature, expressed in millimetres.

N	Mean	SD	Min	Max	Method	Population	Reference
281	20.4	2.7	-	-	photographic analysis	Turkish	[6]
200	18.4	1.9	-	-	direct anthropometry	North American White	[7]

Table S53: Columella length: (sn-c), male subjects. Normotypical values of the linear measurement derived from the literature, expressed in millimetres.

N	Mean	SD	Min	Max	Method	Population	Reference
109	11.5	1.7	-	-	direct anthropometry	North American White	[7]

Table S54: Columella length: (sn-c), female subjects. Normotypical values of the linear measurement derived from the literature, expressed in millimetres.

N	Mean	SD	Min	Max	Method	Population	Reference
200	11.4	1.7	-	-	direct anthropometry	North American White	[7]

Table S55: Physiognomical height of the face: (tr-gn), male subjects. Normotypical values of the linear measurement derived from the literature, expressed in millimetres.

N	Mean	SD	Min	Max	Method	Population	Reference
-	187.5	8.1	171.3	203.7	direct anthropometry	North American White	[2]
30	185.1	9.0	167.1	203.1	direct anthropometry	Azerbaijan	[2]
30	184.3	8.7	166.9	201.7	direct anthropometry	Bulgarian	[2]
30	181.7	7.9	165.9	197.5	direct anthropometry	Chez	[2]
30	180.1	10.6	158.9	201.3	direct anthropometry	Croatian	[2]
30	182.2	11.1	160.0	204.4	direct anthropometry	German	[2]
30	178.7	12.9	152.9	204.5	direct anthropometry	Greek	[2]
30	181.3	14.2	152.9	209.7	direct anthropometry	Hungarian	[2]
30	186.0	10.6	164.8	207.2	direct anthropometry	Italian	[2]
30	181.9	8.2	165.5	198.3	direct anthropometry	Polish	[2]
30	190.7	7.1	176.5	204.9	direct anthropometry	Portuguese	[2]
30	184.4	8.0	168.2	200.2	direct anthropometry	Russian	[2]
30	183.7	8.8	166.1	201.3	direct anthropometry	Slovak	[2]
30	181.3	10.6	160.7	202.9	direct anthropometry	Slovenian	[2]
30	186.5	6.4	173.7	199.3	direct anthropometry	Turkish	[2]
149	185.8	18.4	-	-	photographic analysis	Turkish	[6]
109	187.2	12.1	-	-	direct anthropometry	North American White	[7]

Table S56: Physiognomical height of the face: (tr-gn), female subjects. Normotypical values of the linear measurement derived from the literature, expressed in millimetres.

N	Mean	SD	Min	Max	Method	Population	Reference
-	172.5	7.5	157.5	187.5	direct anthropometry	North American White	[2]
30	175.4	6.8	161.8	189.0	direct anthropometry	Azerbaijan	[2]
30	170.5	6.9	156.9	184.5	direct anthropometry	Bulgarian	[2]
30	182.9	8.1	166.7	199.1	direct anthropometry	Chez	[2]
30	172.6	8.7	155.2	190.0	direct anthropometry	Croatian	[2]
30	170.9	7.2	156.5	185.3	direct anthropometry	German	[2]
30	173.8	6.9	160.0	187.6	direct anthropometry	Greek	[2]
30	169.4	7.7	154.0	184.8	direct anthropometry	Hungarian	[2]
30	171.4	9.2	153.0	189.8	direct anthropometry	Italian	[2]
30	172.1	8.9	154.3	189.9	direct anthropometry	Polish	[2]
30	177.4	9.5	158.4	196.4	direct anthropometry	Portuguese	[2]
30	174.4	8.7	157.0	191.8	direct anthropometry	Russian	[2]
30	169.7	8.7	152.2	186.9	direct anthropometry	Slovak	[2]
30	170.4	15.1	140.2	200.6	direct anthropometry	Slovenian	[2]
30	179.2	9.4	160.4	198.0	direct anthropometry	Turkish	[2]
281	174.0	18.3	-	-	photographic analysis	Turkish	[6]
200	173.3	7.8	-	-	direct anthropometry	North American White	[7]

Table S57: Height of the chin: (*sl-gn*), male subjects. Normotypical values of the linear measurement derived from the literature, expressed in millimetres.

N	Mean	SD	Min	Max	Method	Population	Reference
149	28.5	4.8	-	-	photographic analysis	Turkish	[6]
109	33.1	3.0	-	-	direct anthropometry	North American White	[7]

Table S58: Height of the chin: (*sl-gn*), female subjects. Normotypical values of the linear measurement derived from the literature, expressed in millimetres.

N	Mean	SD	Min	Max	Method	Population	Reference
400	23.5	3.1	-	-	photogrammetry	Caucasian	[13]
281	24.4	3.3	-	-	photographic analysis	Turkish	[6]
200	27.0	2.5	-	-	direct anthropometry	North American White	[7]

Table S59: Depth in the mandibular region (right/left): (*gn-t*), male subjects. Normotypical values of the linear measurement derived from the literature, expressed in millimetres.

N	Mean	SD	Min	Max	Method	Population	Reference
514	151.6	6.3	-	-	digital stereophotogrammetry	Caucasian	[4]
100	147.3	5.9	125.1	170.0	direct anthropometry	Turkish	[5]
109	148.5	5.0	-	-	direct anthropometry	North American White	[7]
514	150.6	6.3	-	-	digital stereophotogrammetry	Caucasian	[4]
109	148.2	5.2	-	-	direct anthropometry	North American White	[7]

Table S60: Depth in the mandibular region (right/left): (*gn-t*), female subjects. Normotypical values of the linear measurement derived from the literature, expressed in millimetres.

N	Mean	SD	Min	Max	Method	Population	Reference
875	139.0	6.0	-	-	digital stereophotogrammetry	Caucasian	[4]
100	135.6	6.6	120.5	149.4	direct anthropometry	Turkish	[5]
200	134.0	5.0	-	-	direct anthropometry	North American White	[7]
881	138.7	6.0	-	-	digital stereophotogrammetry	Caucasian	[4]
200	133.9	5.1	-	-	direct anthropometry	North American White	[7]

Table S61: Physiognomical height of the upper face: (*n-sto*), male subjects. Normotypical values of the linear measurement derived from the literature, expressed in millimetres.

N	Mean	SD	Min	Max	Method	Population	Reference
535	77.7	4.5	-	-	digital stereophotogrammetry	Caucasian	[4]
50	74.0	4.2	-	-	direct anthropometry	North American White	[11]
149	73.5	8.2	-	-	photographic analysis	Turkish	[6]
109	76.6	4.0	-	-	direct anthropometry	North American White	[7]
25	76.1	2.8	72.0	81.0	direct anthropometry	Turkish	[9]
29	76.2	2.5	71.0	82.0	direct anthropometry	Turkish	[9]
36	76.4	4.2	68.0	83.0	direct anthropometry	Turkish	[9]

Table S62: Physiognomical height of the upper face: (*n-sto*), female subjects. Normotypical values of the linear measurement derived from the literature, expressed in millimetres.

N	Mean	SD	Min	Max	Method	Population	Reference
906	74.0	4.4	-	-	digital stereophotogrammetry	Caucasian	[4]
50	68.1	3.4	-	-	direct anthropometry	North American White	[11]
281	70.8	6.5	-	-	photographic analysis	Turkish	[6]
200	69.4	3.2	-	-	direct anthropometry	North American White	[7]
34	70.0	3.3	63.0	78.0	direct anthropometry	Turkish	[9]
20	73.0	4.3	66.0	81.0	direct anthropometry	Turkish	[9]
29	74.4	3.7	70.0	82.0	direct anthropometry	Turkish	[9]

Table S63: Width between the labial insertions of the alar base: ($sbal_r$-$sbal_l$), male subjects. Normotypical values of the linear measurement derived from the literature, expressed in millimetres.

N	Mean	SD	Min	Max	Method	Population	Reference
534	19.3	2.6	-	-	digital stereophotogrammetry	Caucasian	[4]
85	21.0	2.4	-	-	direct anthropometry	North American White	[7]

Table S64: Width between the labial insertions of the alar base: ($sbal_r$-$sbal_l$), female subjects. Normotypical values of the linear measurement derived from the literature, expressed in millimetres.

N	Mean	SD	Min	Max	Method	Population	Reference
905	17.3	2.3	-	-	digital stereophotogrammetry	Caucasian	[4]
45	19.9	1.9	-	-	direct anthropometry	North American White	[7]

Table S65: Length of the ala (right/left): (*prn-ac*), male subjects. Normotypical values of the linear measurement derived from the literature, expressed in millimetres.

N	Mean	SD	Min	Max	Method	Population	Reference
534	35.3	2.1	-	-	digital stereophotogrammetry	Caucasian	[4]
109	35.0	1.6	-	-	direct anthropometry	North American White	[7]
533	35.4	2.2	-	-	digital stereophotogrammetry	Caucasian	[4]
109	35.0	1.7	-	-	direct anthropometry	North American White	[7]

Table S66: Length of the ala (right/left): (*prn-ac*), female subjects. Normotypical values of the linear measurement derived from the literature, expressed in millimetres.

N	Mean	SD	Min	Max	Method	Population	Reference
905	31.4	1.9	-	-	digital stereophotogrammetry	Caucasian	[4]
199	31.5	1.8	-	-	direct anthropometry	North American White	[7]
904	31.5	1.9	-	-	digital stereophotogrammetry	Caucasian	[4]
198	31.4	1.8	-	-	direct anthropometry	North American White	[7]

Table S67: Width of the philtrum: (cph_r-cph_l), male subjects. Normotypical values of the linear measurement derived from the literature, expressed in millimetres.

N	Mean	SD	Min	Max	Method	Population	Reference
534	13.3	1.8	-	-	digital stereophotogrammetry	Caucasian	[4]
149	13.9	2.2	-	-	photographic analysis	Turkish	[6]
108	10.4	1.4	-	-	direct anthropometry	North American White	[7]

Table S68: Width of the philtrum: (cph_r-cph_l), female subjects. Normotypical values of the linear measurement derived from the literature, expressed in millimetres.

N	Mean	SD	Min	Max	Method	Population	Reference
907	11.8	1.6	-	-	digital stereophotogrammetry	Caucasian	[4]
400	12.3	2.1	-	-	photogrammetry	Caucasian	[13]
281	12.1	1.9	-	-	photographic analysis	Turkish	[6]
200	9.7	1.5	-	-	direct anthropometry	North American White	[7]

Table S69: Lower Lip Height: (*sto-sl*), male subjects. Normotypical values of the linear measurement derived from the literature, expressed in millimetres.

N	Mean	SD	Min	Max	Method	Population	Reference
524	19.1	2.9	-	-	digital stereophotogrammetry	Caucasian	[4]
149	19.5	3.5	-	-	photographic analysis	Turkish	[6]
109	19.7	2.1	-	-	direct anthropometry	North American White	[7]

Table S70: Lower Lip Height: (*sto-sl*), female subjects. Normotypical values of the linear measurement derived from the literature, expressed in millimetres.

N	Mean	SD	Min	Max	Method	Population	Reference
904	17.7	2.3	-	-	digital stereophotogrammetry	Caucasian	[4]
400	14.6	1.8	-	-	photogrammetry	Caucasian	[13]
281	17.5	2.3	-	-	photographic analysis	Turkish	[6]
200	17.8	4.7	-	-	direct anthropometry	North American White	[7]

Table S71: Height of the coutaneous lower lip: (*li-sl*), male subjects. Normotypical values of the linear measurement derived from the literature, expressed in millimetres.

N	Mean	SD	Min	Max	Method	Population	Reference
524	12.1	2.6	-	-	digital stereophotogrammetry	Caucasian	[4]
149	10.2	2.9	-	-	photographic analysis	Turkish	[6]
109	11.9	2.2	-	-	direct anthropometry	North American White	[7]

Table S72: Height of the coutaneous lower lip: (*li-sl*), female subjects. Normotypical values of the linear measurement derived from the literature, expressed in millimetres.

N	Mean	SD	Min	Max	Method	Population	Reference
904	10.6	2.2	-	-	digital stereophotogrammetry	Caucasian	[4]
281	8.4	1.8	-	-	photographic analysis	Turkish	[6]
200	10.7	2.1	-	-	direct anthropometry	North American White	[7]

Table S73: Width of the forehead: (ft_r-ft_l), male subjects. Normotypical values of the linear measurement derived from the literature, expressed in millimetres.

N	Mean	SD	Min	Max	Method	Population	Reference
520	111.3	8.7	-	-	direct anthropometry	Caucasian	[4]
100	118.4	6.2	104.5	137.7	direct anthropometry	Turkish	[5]
149	109.6	11.7	-	-	photographic analysis	Turkish	[6]
109	115.9	5.2	-	-	direct anthropometry	North American White	[7]
25	127.1	8.6	110.0	143.0	direct anthropometry	Turkish	[9]
29	121.4	5.2	112.0	132.0	direct anthropometry	Turkish	[9]
36	119.3	7.3	107.0	135.1	direct anthropometry	Turkish	[9]

Table S74: Width of the forehead: (ft_r-ft_l), female subjects. Normotypical values of the linear measurement derived from the literature, expressed in millimetres.

N	Mean	SD	Min	Max	Method	Population	Reference
845	105.2	7.8	-	-	direct anthropometry	Caucasian	[4]
100	113.6	5.3	99.3	126.8	direct anthropometry	Turkish	[5]
281	102.9	8.4	-	-	photographic analysis	Turkish	[6]
199	111.5	4.4	-	-	direct anthropometry	North American White	[7]
34	115.3	5.9	100.0	126.0	direct anthropometry	Turkish	[9]
20	112.8	5.8	101.0	126.0	direct anthropometry	Turkish	[9]
29	114.9	5.2	107.0	127.0	direct anthropometry	Turkish	[9]

Table S75: Depth of the lower jaw (right/left): (*gn-Go*), male subjects. Normotypical values of the linear measurement derived from the literature, expressed in millimetres.

N	Mean	SD	Min	Max	Method	Population	Reference
35	86.6	5.2	76.2	97.0	direct anthropometry	North American White	[16]
35	86.8	5.2	76.4	97.2	direct anthropometry	North American White	[16]

Table S76: Depth of the lower jaw (right/left): (*gn-Go*), female subjects. Normotypical values of the linear measurement derived from the literature, expressed in millimetres.

N	Mean	SD	Min	Max	Method	Population	Reference
40	81.3	3.7	73.9	88.7	direct anthropometry	North American White	[16]
40	81.8	3.7	74.4	89.2	direct anthropometry	North American White	[16]

Table S77: Orbito-glabellar distance (right/left): (*ex-g*), male subjects. Normotypical values of the linear measurement derived from the literature, expressed in millimetres.

N	Mean	SD	Min	Max	Method	Population	Reference
35	52.3	2.8	-	-	direct anthropometry	North American White	[7]
35	52.2	2.9	-	-	direct anthropometry	North American White	[7]

Table S78: Orbito-glabellar distance (right/left): (*ex-g*), female subjects. Normotypical values of the linear measurement derived from the literature, expressed in millimetres.

N	Mean	SD	Min	Max	Method	Population	Reference
40	48.9	2.8	-	-	direct anthropometry	North American White	[7]
40	48.9	2.7	-	-	direct anthropometry	North American White	[7]

Table S79: Height of the mandible: (*sto-gn*), male subjects. Normotypical values of the linear measurement derived from the literature, expressed in millimetres.

N	Mean	SD	Min	Max	Method	Population	Reference
149	47.8	6.2	-	-	photographic analysis	Turkish	[6]
109	50.7	4.0	-	-	direct anthropometry	North American White	[7]
25	45.9	7.3	35.0	68.0	direct anthropometry	Turkish	[9]
29	47.7	4.0	38.0	58.0	direct anthropometry	Turkish	[9]
36	48.5	5.5	36.0	57.0	direct anthropometry	Turkish	[9]

Table S80: Height of the mandible: (*sto-gn*), female subjects. Normotypical values of the linear measurement derived from the literature, expressed in millimetres.

N	Mean	SD	Min	Max	Method	Population	Reference
281	42.1	7.0	-	-	photographic analysis	Turkish	[6]
200	43.4	3.1	-	-	direct anthropometry	North American White	[7]
34	42.3	3.2	36.0	47.0	direct anthropometry	Turkish	[9]
20	44.0	3.1	39.0	50.0	direct anthropometry	Turkish	[9]
29	44.0	2.8	38.0	48.0	direct anthropometry	Turkish	[9]

Table S81: Width of the nostril floor (right/left): (*sbal-sn*), male subjects. Normotypical values of the linear measurement derived from the literature, expressed in millimetres.

N	Mean	SD	Min	Max	Method	Population	Reference
149	11.1	2.1	-	-	photographic analysis	Turkish	[6]
109	12.9	1.7	-	-	direct anthropometry	North American White	[7]
149	10.0	1.9	-	-	photographic analysis	Turkish	[6]
109	12.8	1.7	-	-	direct anthropometry	North American White	[7]

Table S82: Width of the nostril floor (right/left): (*sbal-sn*), female subjects. Normotypical values of the linear measurement derived from the literature, expressed in millimetres.

N	Mean	SD	Min	Max	Method	Population	Reference
281	10.0	1.7	-	-	photographic analysis	Turkish	[6]
200	10.9	1.4	-	-	direct anthropometry	North American White	[7]
281	8.8	1.6	-	-	photographic analysis	Turkish	[6]
200	10.9	1.5	-	-	direct anthropometry	North American White	[7]

Table S83: Height of the upper profile: (*tr-prn*), male subjects. Normotypical values of the linear measurement derived from the literature, expressed in millimetres.

N	Mean	SD	Min	Max	Method	Population	Reference
109	115.3	9.3	-	-	direct anthropometry	North American White	[7]

Table S84: Height of the upper profile: (*tr-prn*), female subjects. Normotypical values of the linear measurement derived from the literature, expressed in millimetres.

N	Mean	SD	Min	Max	Method	Population	Reference
197	106.4	8.1	-	-	direct anthropometry	North American White	[7]

Table S85: Height of the lower profile: (*prn-gn*), male subjects. Normotypical values of the linear measurement derived from the literature, expressed in millimetres.

N	Mean	SD	Min	Max	Method	Population	Reference
109	91.7	5.6	-	-	direct anthropometry	North American White	[7]

Table S86: Height of the lower profile: (*prn-gn*), female subjects. Normotypical values of the linear measurement derived from the literature, expressed in millimetres.

N	Mean	SD	Min	Max	Method	Population	Reference
200	81.4	4.6	-	-	direct anthropometry	North American White	[7]

Table S87: Height of the mandibular ramus (right/left): (*Go-cdl*), male subjects. Normotypical values of the linear measurement derived from the literature, expressed in millimetres.

N	Mean	SD	Min	Max	Method	Population	Reference
35	67.1	5.3	-	-	direct anthropometry	North American White	[7]
35	67.7	5.3	-	-	direct anthropometry	North American White	[7]

Table S88: Height of the mandibular ramus (right/left): (*Go-cdl*), female subjects. Normotypical values of the linear measurement derived from the literature, expressed in millimetres.

N	Mean	SD	Min	Max	Method	Population	Reference
40	61.6	5.0	-	-	direct anthropometry	North American White	[7]
40	62.2	4.7	-	-	direct anthropometry	North American White	[7]

Table S89: Depth of the supraorbital rim (right/left): (*t-g*), male subjects. Normotypical values of the linear measurement derived from the literature, expressed in millimetres.

N	Mean	SD	Min	Max	Method	Population	Reference
109	129.5	5.8	-	-	direct anthropometry	North American White	[7]
109	129.2	5.7	-	-	direct anthropometry	North American White	[7]

Table S90: Depth of the supraorbital rim (right/left): (*t-g*), female subjects. Normotypical values of the linear measurement derived from the literature, expressed in millimetres.

N	Mean	SD	Min	Max	Method	Population	Reference
193	119.7	4.2	-	-	direct anthropometry	North American White	[7]
193	118.9	5.6	-	-	direct anthropometry	North American White	[7]

Table S91: Endocanthion-facial midline distance (right/left): (*en-se*), male subjects. Normotypical values of the linear measurement derived from the literature, expressed in millimetres.

N	Mean	SD	Min	Max	Method	Population	Reference
109	25.6	2.1	-	-	direct anthropometry	North American White	[7]
109	25.3	2.1	-	-	direct anthropometry	North American White	[7]

Table S92: Endocanthion-facial midline distance (right/left): (*en-se*), female subjects. Normotypical values of the linear measurement derived from the literature, expressed in millimetres.

N	Mean	SD	Min	Max	Method	Population	Reference
199	21.9	1.6	-	-	direct anthropometry	North American White	[7]
199	22.0	1.5	-	-	direct anthropometry	North American White	[7]

Table S93: Orbitotragial distance (right/left): (*ex-t*), male subjects. Normotypical values of the linear measurement derived from the literature, expressed in millimetres.

N	Mean	SD	Min	Max	Method	Population	Reference
109	85.6	3.2	-	-	direct anthropometry	North American White	[7]
109	84.4	3.3	-	-	direct anthropometry	North American White	[7]

Table S94: Orbitotragial distance (right/left): (*ex-t*), female subjects. Normotypical values of the linear measurement derived from the literature, expressed in millimetres.

N	Mean	SD	Min	Max	Method	Population	Reference
199	78.9	3.7	-	-	direct anthropometry	North American White	[7]
199	78.2	3.5	-	-	direct anthropometry	North American White	[7]

Table S95: Orbitogonial distance (right/left): (*ex-Go*), male subjects. Normotypical values of the linear measurement derived from the literature, expressed in millimetres.

N	Mean	SD	Min	Max	Method	Population	Reference
109	103.9	4.7	-	-	direct anthropometry	North American White	[7]
109	103.6	4.5	-	-	direct anthropometry	North American White	[7]

Table S96: Orbitogonial distance (right/left): (*ex-Go*), female subjects. Normotypical values of the linear measurement derived from the literature, expressed in millimetres.

N	Mean	SD	Min	Max	Method	Population	Reference
198	93.0	4.5	-	-	direct anthropometry	North American White	[7]
198	92.4	4.4	-	-	direct anthropometry	North American White	[7]

Table S97: Combined height of orbit and eyebrow (right/left): (*or-sci*), male subjects. Normotypical values of the linear measurement derived from the literature, expressed in millimetres.

N	Mean	SD	Min	Max	Method	Population	Reference
109	38.0	3.5	-	-	direct anthropometry	North American White	[7]
109	38.0	3.5	-	-	direct anthropometry	North American White	[7]

Table S98: Combined height of orbit and eyebrow (right/left): (*or-sci*), female subjects. Normotypical values of the linear measurement derived from the literature, expressed in millimetres.

N	Mean	SD	Min	Max	Method	Population	Reference
195	38.4	3.4	-	-	direct anthropometry	North American White	[7]
195	38.3	3.3	-	-	direct anthropometry	North American White	[7]

Table S99: Height of eye fissure (right/left): (*ps-pi*), male subjects. Normotypical values of the linear measurement derived from the literature, expressed in millimetres.

N	Mean	SD	Min	Max	Method	Population	Reference
109	10.8	0.9	-	-	direct anthropometry	North American White	[7]

Table S100: Height of eye fissure: (*ps-pi*), female subjects. Normotypical values of the linear measurement derived from the literature, expressed in millimetres.

N	Mean	SD	Min	Max	Method	Population	Reference
200	10.9	1.2	-	-	direct anthropometry	North American White	[7]

Table S101: Height of the upper lid (right/left): (*ps-os*), male subjects. Normotypical values of the linear measurement derived from the literature, expressed in millimetres.

N	Mean	SD	Min	Max	Method	Population	Reference
40	11.2	2.0	-	-	direct anthropometry	North American White	[7]

Table S102: Height of the upper lid: (*ps-os*), female subjects. Normotypical values of the linear measurement derived from the literature, expressed in millimetres.

N	Mean	SD	Min	Max	Method	Population	Reference
40	12.6	2.6	-	-	direct anthropometry	North American White	[7]

Table S103: Height of the lower lid (right/left): direct linear measurment (*pi-or*), male subjects. Normotypical values of the linear measurement derived from the literature, expressed in millimetres.

N	Mean	SD	Min	Max	Method	Population	Reference
40	8.1	1.6	-	-	direct anthropometry	North American White	[7]
40	8.1	1.5	-	-	direct anthropometry	North American White	[7]

Table S104: Height of the lower lid (right/left): (*pi-or*), female subjects. Normotypical values of the linear measurement derived from the literature, expressed in millimetres.

N	Mean	SD	Min	Max	Method	Population	Reference
40	7.5	1.7	-	-	direct anthropometry	North American White	[7]
40	7.5	1.7	-	-	direct anthropometry	North American White	[7]

Table S105: Width of the columella: (sn'_r-sn'_l), male subjects. Normotypical values of the linear measurement derived from the literature, expressed in millimetres.

N	Mean	SD	Min	Max	Method	Population	Reference
109	6.9	0.7	-	-	direct anthropometry	North American White	[7]

Table S106: Width of the columella: (sn'_r-sn'_l), female subjects. Normotypical values of the linear measurement derived from the literature, expressed in millimetres.

N	Mean	SD	Min	Max	Method	Population	Reference
200	6.8	0.7	-	-	direct anthropometry	North American White	[7]

Table S107: Distance between the facial insertions of the alar base and the subnasal point (right/left): (*ac-sn*), male subjects. Normotypical values of the linear measurement derived from the literature, expressed in millimetres.

N	Mean	SD	Min	Max	Method	Population	Reference
86	23.2	1.8	-	-	direct anthropometry	North American White	[7]
86	23.2	1.8	-	-	direct anthropometry	North American White	[7]

Table S108: Distance between the facial insertions of the alar base and the subnasal point (right/left): (*ac-sn*), female subjects. Normotypical values of the linear measurement derived from the literature, expressed in millimetres.

N	Mean	SD	Min	Max	Method	Population	Reference
45	20.9	1.5	-	-	direct anthropometry	North American White	[7]
45	20.9	1.8	-	-	direct anthropometry	North American White	[7]

Table S109: Labio-aural distance (right/left): (*ch-t*), male subjects. Normotypical values of the linear measurement derived from the literature, expressed in millimetres.

N	Mean	SD	Min	Max	Method	Population	Reference
109	114.5	5.0	-	-	direct anthropometry	North American White	[7]
109	113.6	4.8	-	-	direct anthropometry	North American White	[7]

Table S110: Labio-aural distance (right/left): (*ch-t*), female subjects. Normotypical values of the linear measurement derived from the literature, expressed in millimetres.

N	Mean	SD	Min	Max	Method	Population	Reference
194	104.1	4.6	-	-	direct anthropometry	North American White	[7]
194	103.5	4.9	-	-	direct anthropometry	North American White	[7]

Table S111: Height of the orbit (right/left) : (*or-os*), male subjects. Normotypical values of the linear measurement derived from the literature, expressed in millimetres.

N	Mean	SD	Min	Max	Method	Population	Reference
40	29.5	5.3	-	-	direct anthropometry	North American White	[7]
40	29.8	4.2	-	-	direct anthropometry	North American White	[7]

Table S112: Height of the orbit (right/left): (*or-os*), female subjects. Normotypical values of the linear measurement derived from the literature, expressed in millimetres.

N	Mean	SD	Min	Max	Method	Population	Reference
40	29.4	3.1	-	-	direct anthropometry	North American White	[7]
40	29.4	3.2	-	-	direct anthropometry	North American White	[7]

Table S113: Angle of facial convexity (*n-sn-pg*), male subjects. Normotypical values of the angular measurement derived from the literature, expressed in degrees.

N	Mean	SD	Min	Max	Method	Population	Reference
40	168.0	5.7	-	-	infrared camera	Caucasian	[1]

Table S114: Angle of facial convexity (*n-sn-pg*), female subjects. Normotypical values of the angular measurement derived from the literature, expressed in degrees.

N	Mean	SD	Min	Max	Method	Population	Reference
40	166.8	5.3	-	-	infrared camera	Caucasian	[1]
12	160.2	5	-	-	computerized electromagnetic digitizer	Caucasian	[12]
12	162.7	4.6	-	-	computerized electromagnetic digitizer	Caucasian	[12]
71	160.4	6.3	-	-	computerized electromagnetic digitizer	Caucasian	[12]
66	163.6	4.4	152.7	172.1	3D facial scanner	Caucasian	[10]

Table S115: Nasomental angle (*n-prn-pg*), male subjects. Normotypical values of the angular measurement derived from the literature, expressed in degrees.

N	Mean	SD	Min	Max	Method	Population	Reference
40	134.2	5.5	-	-	infrared camera	Caucasian	[1]
247	129.4	-	-	-	photogrammetry	Caucasian	[3]

Table S116: Nasomental angle (*n-prn-pg*), female subjects. Normotypical values of the angular measurement derived from the literature, expressed in degrees.

N	Mean	SD	Min	Max	Method	Population	Reference
40	134.0	5.0	-	-	infrared camera	Caucasian	[1]
66	131.1	4.1	123.3	142.3	3D facial scanner	Caucasian	[10]
264	129.5	-	-	-	photogrammetry	Caucasian	[3]
84	130.7	4	-	-	stereophotogrammetry	Caucasian Brazilian	[17]

Table S117: Exocanthion–nasion angle (ex_l-n-ex_r), male subjects. Normotypical values of the angular measurement derived from the literature, expressed in degrees.

N	Mean	SD	Min	Max	Method	Population	Reference
40	109.8	7.6	-	-	infrared camera	Caucasian	[1]

Table S118: Exocanthion–nasion angle (ex_l-n-ex_r), female subjects. Normotypical values of the angular measurement derived from the literature, expressed in degrees.

N	Mean	SD	Min	Max	Method	Population	Reference
40	110	7.9	-	-	infrared camera	Caucasian	[1]
12	124.9	3.5	-	-	computerized electromagnetic digitizer	Caucasian	[12]
12	120.8	4.2	-	-	computerized electromagnetic digitizer	Caucasian	[12]
71	120.2	5.6	-	-	computerized electromagnetic digitizer	Caucasian	[12]
66	126.2	4.9	114.5	137.5	3D facial scanner	Caucasian	[10]

Table S119: Nasofrontal angle (*g-n-prn*), male subjects. Normotypical values of the angular measurement derived from the literature, expressed in degrees.

N	Mean	SD	Min	Max	Method	Population	Reference
360	137.9	-	-	-	photogrammetry	Caucasian	[3]
46	146.0	8.2	129.3	161.5	angular photogrammetry	Anatolian Turkish	[18]
50	138.6	6.8	124	153.6	lateral photogrammetry	Caucasian	[19]
109	130.3	7.4	-	-	direct anthropometry	North American White	[7]

Table S120: Nasofrontal angle (*g-n-prn*), female subjects. Normotypical values of the angular measurement derived from the literature, expressed in degrees.

N	Mean	SD	Min	Max	Method	Population	Reference
30	136.6	7.8	117.2	151	photography	Caucasian	[20]
30	140.7	7.8	112.5	151	photography	Caucasian	[20]
628	140.6	-	-	-	photogrammetry	Caucasian	[3]
54	148.6	6.7	123.7	158.1	angular photogrammetry	Anatolian Turkish	[18]
162	142.0	6.1	122.2	156.3	lateral photogrammetry	Caucasian	[19]
84	143.6	5.7	-	-	stereophotogrammetry	Caucasian Brazilian	[17]
200	134.3	7	-	-	direct anthropometry	North American White	[7]

Table S121: Nasal tip angle (*n-prn-sn*), male subjects. Normotypical values of the angular measurement derived from the literature, expressed in degrees.

N	Mean	SD	Min	Max	Method	Population	Reference
56	82.2	10.0	-	-	direct anthropometry	Turkish	[21]
126	93.8	6.3	-	-	digital anthropometry	Caucasian	[22]

Table S122: Nasal tip angle (*n-prn-sn*), female subjects. Normotypical values of the angular measurement derived from the literature, expressed in degrees.

N	Mean	SD	Min	Max	Method	Population	Reference
30	79.7	7	64	91	photography	Caucasian	[20]
30	82	6.7	63.5	91	photography	Caucasian	[20]
59	77.9	9.8	-	-	direct anthropometry	Turkish	[21]
66	95.0	5.0	-	-	digital anthropometry	Caucasian	[22]

Table S123: Angle of total facial convexity (*g-sn-pg*), male subjects. Normotypical values of the angular measurement derived from the literature, expressed in degrees.

N	Mean	SD	Min	Max	Method	Population	Reference
340	167.8	-	-	-	photogrammetry	Caucasian	[3]
46	170.6	6.2	161	188	angular photogrammetry	Anatolian Turkish	[18]
50	168.2	5.0	160	178.4	lateral photogrammetry	Caucasian	[19]

Table S124: Angle of total facial convexity (*g-sn-pg*), female subjects. Normotypical values of the angular measurement derived from the literature, expressed in degrees.

N	Mean	SD	Min	Max	Method	Population	Reference
30	168.1	4.1	158.5	177	photography	Caucasian	[20]
30	166.2	6.4	154.5	179	photography	Caucasian	[20]
506	168.2	-	-	-	photogrammetry	Caucasian	[3]
54	168.8	5.4	157.4	179.8	angular photogrammetry	Anatolian Turkish	[18]
162	167	5.4	150.8	179	lateral photogrammetry	Caucasian	[19]

Table S125: Nasofacial angle (*g-prn-pg*), male subjects. Normotypical values of the angular measurement derived from the literature, expressed in degrees.

N	Mean	SD	Min	Max	Method	Population	Reference
191	141.5	-	-	-	photogrammetry	Caucasian	[3]
46	142.3	5.4	133.0	154.6	angular photogrammetry	Anatolian Turkish	[18]
50	139.9	5.4	126.9	152.5	lateral photogrammetry	Caucasian	[19]

Table S126: Nasofacial angle (*g-prn-pg*), female subjects. Normotypical values of the angular measurement derived from the literature, expressed in degrees.

N	Mean	SD	Min	Max	Method	Population	Reference
334	141	-	-	-	photogrammetry	Caucasian	[3]
54	142.6	5.3	128.5	159.1	angular photogrammetry	Anatolian Turkish	[18]
162	139.2	4.5	128	149.6	lateral photogrammetry	Caucasian	[19]

Table S127: Tragion–nasion–tragion angle (t_l-n-t_r), male subjects. Normotypical values of the angular measurement derived from the literature, expressed in degrees.

N	Mean	SD	Min	Max	Method	Population	Reference
40	71.6	5.0	-	-	infrared camera	Caucasian	[1]

Table S128: Tragion–nasion–tragion angle (t_l-n-t_r), female subjects. Normotypical values of the angular measurement derived from the literature, expressed in degrees.

N	Mean	SD	Min	Max	Method	Population	Reference
40	69.7	4.9	-	-	infrared camera	Caucasian	[1]
66	73.5	2.1	68.5	79.1	3D facial scanner	Caucasian	[10]

Table S129: Tragion–pronasale–tragion angle (t_l-prn-t_r), male subjects. Normotypical values of the angular measurement derived from the literature, expressed in degrees.

N	Mean	SD	Min	Max	Method	Population	Reference
40	63.6	4.6	-	-	infrared camera	Caucasian	[1]

Table S130: Tragion–pronasale–tragion angle (t_l-prn-t_r), female subjects. Normotypical values of the angular measurement derived from the literature, expressed in degrees.

N	Mean	SD	Min	Max	Method	Population	Reference
40	62.4	3.8	-	-	infrared camera	Caucasian	[1]
66	64.1	1.8	59.3	68.0	3D facial scanner	Caucasian	[10]

Table S131: Tragion–pogonion–tragion angle (t_l-pg-t_r), male subjects. Normotypical values of the angular measurement derived from the literature, expressed in degrees.

N	Mean	SD	Min	Max	Method	Population	Reference
40	61.6	4.8	-	-	infrared camera	Caucasian	[1]

Table S132: Tragion–pogonion–tragion angle (t_l-pg-t_r), female subjects. Normotypical values of the angular measurement derived from the literature, expressed in degrees.

N	Mean	SD	Min	Max	Method	Population	Reference
40	61.2	4.2	-	-	infrared camera	Caucasian	[1]
66	62.3	1.8	58.5	67.1	3D facial scanner	Caucasian	[10]

Table S133: Nasolabial angle (c-sn-ls), male subjects. Normotypical values of the angular measurement derived from the literature, expressed in degrees.

N	Mean	SD	Min	Max	Method	Population	Reference
360	100.1	-	-	-	photogrammetry	Caucasian	[3]
50	98.9	8	-	-	direct anthropometry	North American White	[11]
30	111.4	10.8	-	-	Lateral cephalometric radiographs	Caucasian	[23]
-	102.9	10.5	-	-	Lateral cephalometric radiographs	Turkish	[24]
-	112.6	10.6	-	-	Lateral cephalometric radiographs	European–American Caucasian	[24]
46	101.1	10.2	75.4	126.9	angular photogrammetry	Anatolian Turkish	[18]
50	105.2	13.3	71.7	137.6	lateral photogrammetry	Caucasian	[19]
109	99.8	11.8	-	-	direct anthropometry	North American White	[7]

Table S134: Nasolabial angle (c-sn-ls), female subjects. Normotypical values of the angular measurement derived from the literature, expressed in degrees.

N	Mean	SD	Min	Max	Method	Population	Reference
628	103.3	-	-	-	photogrammetry	Caucasian	[3]
50	99.1	8.7	-	-	direct anthropometry	North American White	[11]
60	111.6	9.5	-	-	Lateral cephalometric radiographs	Caucasian	[23]
-	107.7	8.6	-	-	Lateral cephalometric radiographs	Turkish	[24]
-	111.1	9.7	-	-	Lateral cephalometric radiographs	European–American Caucasian	[24]
54	102.9	10.4	81.7	129.9	angular photogrammetry	Anatolian Turkish	[18]
162	107.6	8.5	76.5	134.5	lateral photogrammetry	Caucasian	[19]
200	104.2	9.8	-	-	direct anthropometry	North American White	[7]
30	108.3	10.4	85	129.5	photography	Caucasian	[20]
30	114.6	10.3	98	141.5	photography	Caucasian	[20]

Table S135: Labiomental angle (*li-sl-pg*), male subjects. Normotypical values of the angular measurement derived from the literature, expressed in degrees.

N	Mean	SD	Min	Max	Method	Population	Reference
290	128.6	-	-	-	photogrammetry	Caucasian	[3]
44	113.5	20.7	-	-	direct anthropometry	North American White	[7]

Table S136: Labiomental angle (*li-sl-pg*), female subjects. Normotypical values of the angular measurement derived from the literature, expressed in degrees.

N	Mean	SD	Min	Max	Method	Population	Reference
456	132	-	-	-	photogrammetry	Caucasian	[3]
84	137.7	12.5	-	-	stereophotogrammetry	Caucasian Brazilian	[17]
45	121.4	14.4	-	-	direct anthropometry	North American White	[7]

Table S137: Midface convexity angle (right/left) (*n-t-sn*), male subjects. Normotypical values of the angular measurement derived from the literature, expressed in degrees.

N	Mean	SD	Min	Max	Method	Population	Reference
46	29.9	2.4	25.8	37.4	angular photogrammetry	Anatolian Turkish	[18]
50	28.9	2.6	23.5	34.5	lateral photogrammetry	Caucasian	[19]

Table S138: Midface convexity angle (right/left) (*n-t-sn*), female subjects. Normotypical values of the angular measurement derived from the literature, expressed in degrees.

N	Mean	SD	Min	Max	Method	Population	Reference
54	29.3	2.6	24.3	35.9	angular photogrammetry	Anatolian Turkish	[18]
162	28.2	2.6	22.8	34.3	lateral photogrammetry	Caucasian	[19]

Table S139: Inclination of the upper face profile (*g-sn/y-plane*), male subjects. Normotypical values of the angular measurement derived from the literature, expressed in degrees.

N	Mean	SD	Min	Max	Method	Population	Reference
109	2.4	3.3	-	-	direct anthropometry	North American White	[7]

Table S140: Inclination of the upper face profile (*g-sn/y-plane*), female subjects. Normotypical values of the angular measurement derived from the literature, expressed in degrees.

N	Mean	SD	Min	Max	Method	Population	Reference
200	1.7	3.1	-	-	direct anthropometry	North American White	[7]

Table S141: Inclination of the anterior surface of the forehead (*tr-g/y-plane*), male subjects. Normotypical values of the angular measurement derived from the literature, expressed in degrees.

N	Mean	SD	Min	Max	Method	Population	Reference
109	-9.8	4.4	-	-	direct anthropometry	North American White	[7]

Table S142: Inclination of the anterior surface of the forehead (*tr-g/y-plane*), female subjects. Normotypical values of the angular measurement derived from the literature, expressed in degrees.

N	Mean	SD	Min	Max	Method	Population	Reference
200	-5.9	5.2	-	-	direct anthropometry	North American White	[7]

Table S143: Inclination of the Leiber line (*g-ls/y-plane*), male subjects. Normotypical values of the angular measurement derived from the literature, expressed in degrees.

N	Mean	SD	Min	Max	Method	Population	Reference
109	1.3	4.4	-	-	direct anthropometry	North American White	[7]

Table S144: Inclination of the Leiber line (*g-ls/y-plane*), female subjects. Normotypical values of the angular measurement derived from the literature, expressed in degrees.

N	Mean	SD	Min	Max	Method	Population	Reference
200	1.6	2.5	-	-	direct anthropometry	North American White	[7]

Table S145: Inclination of the lower face profile (*sn-pg/y-plane*), male subjects. Normotypical values of the angular measurement derived from the literature, expressed in degrees.

N	Mean	SD	Min	Max	Method	Population	Reference
109	-10.6	5.3	-	-	direct anthropometry	North American White	[7]

Table S146: Inclination of the lower face profile (*sn-pg/y-plane*), female subjects. Normotypical values of the angular measurement derived from the literature, expressed in degrees.

N	Mean	SD	Min	Max	Method	Population	Reference
200	-13.3	4.5	-	-	direct anthropometry	North American White	[7]

Table S147: Inclination of the mandible (*li-pg/y-plane*), male subjects. Normotypical values of the angular measurement derived from the literature, expressed in degrees.

N	Mean	SD	Min	Max	Method	Population	Reference
109	-15	6.6	-	-	direct anthropometry	North American White	[7]

Table S148: Inclination of the mandible (*li-pg/y-plane*), female subjects. Normotypical values of the angular measurement derived from the literature, expressed in degrees.

N	Mean	SD	Min	Max	Method	Population	Reference
200	-19.4	6.9	-	-	direct anthropometry	North American White	[7]

Table S149: Inclination of the chin (*sl-pg/y-plane*), male subjects. Normotypical values of the angular measurement derived from the literature, expressed in degrees.

N	Mean	SD	Min	Max	Method	Population	Reference
109	16.7	10.4	-	-	direct anthropometry	North American White	[7]

Table S150: Inclination of the chin (*sl-pg/y-plane*), female subjects. Normotypical values of the angular measurement derived from the literature, expressed in degrees.

N	Mean	SD	Min	Max	Method	Population	Reference
200	9.1	9.6	-	-	direct anthropometry	North American White	[7]

Table S151: Inclination of the general profile line (*g-pg/y-plane*), male subjects. Normotypical values of the angular measurement derived from the literature, expressed in degrees.

N	Mean	SD	Min	Max	Method	Population	Reference
109	-3	3.4	-	-	direct anthropometry	North American White	[7]

Table S152: Inclination of the general profile line (*g-pg/y-plane*), female subjects. Normotypical values of the angular measurement derived from the literature, expressed in degrees.

N	Mean	SD	Min	Max	Method	Population	Reference
200	-4.1	3	-	-	direct anthropometry	North American White	[7]

Table S153: Mentocervical angle (*sl-pg/gn-gn′*), male subjects. Normotypical values of the angular measurement derived from the literature, expressed in degrees.

N	Mean	SD	Min	Max	Method	Population	Reference
40	78.3	7.9	-	-	direct anthropometry	North American White	[7]

Table S154: Mentocervical angle (*sl-pg/gn-gn′*), female subjects. Normotypical values of the angular measurement derived from the literature, expressed in degrees.

N	Mean	SD	Min	Max	Method	Population	Reference
42	83.9	9.3	-	-	direct anthropometry	North American White	[7]

Table S155: Inclination of the nasal bridge (*n-prn/y-plane*), male subjects. Normotypical values of the angular measurement derived from the literature, expressed in degrees.

N	Mean	SD	Min	Max	Method	Population	Reference
109	30.4	3.6	-	-	direct anthropometry	North American White	[7]

Table S156: Inclination of the nasal bridge (*n-prn/y-plane*), female subjects. Normotypical values of the angular measurement derived from the literature, expressed in degrees.

N	Mean	SD	Min	Max	Method	Population	Reference
200	29.9	3.9	-	-	direct anthropometry	North American White	[7]

Table S157: Inclination of the columella (*sn-c/y-plane*), male subjects. Normotypical values of the angular measurement derived from the literature, expressed in degrees.

N	Mean	SD	Min	Max	Method	Population	Reference
109	77.9	8.9	-	-	direct anthropometry	North American White	[7]

Table S158: Inclination of the columella (*sn-c/y-plane*), female subjects. Normotypical values of the angular measurement derived from the literature, expressed in degrees.

N	Mean	SD	Min	Max	Method	Population	Reference
45	78.2	7.5	-	-	direct anthropometry	North American White	[7]

Table S159: Inclination of the nasal tip portion (*c-prn/y-plane*), male subjects. Normotypical values of the angular measurement derived from the literature, expressed in degrees.

N	Mean	SD	Min	Max	Method	Population	Reference
67	61.7	7.1	-	-	direct anthropometry	North American White	[7]

Table S160: Inclination of the nasal tip portion (*c-prn/y-plane*), female subjects. Normotypical values of the angular measurement derived from the literature, expressed in degrees.

N	Mean	SD	Min	Max	Method	Population	Reference
45	34.3	11.2	-	-	direct anthropometry	North American White	[7]

Table S161: Nasal root-slope angle (mf_r-*se*-mf_l), male subjects. Normotypical values of the angular measurement derived from the literature, expressed in degrees.

N	Mean	SD	Min	Max	Method	Population	Reference
42	44.8	6.9	-	-	direct anthropometry	North American White	[7]

Table S162: Nasal root-slope angle (mf_r-se-mf_l), female subjects. Normotypical values of the angular measurement derived from the literature, expressed in degrees.

N	Mean	SD	Min	Max	Method	Population	Reference
45	43.9	7.7	-	-	direct anthropometry	North American White	[7]

Table S163: Deviation of the nasal bridge (*n-prn/z-plane*), male subjects. Normotypical values of the angular measurement derived from the literature, expressed in degrees.

N	Mean	SD	Min	Max	Method	Population	Reference
40	4.6	2.5	-	-	direct anthropometry	North American White	[7]

Table S164: Deviation of the nasal bridge (*n-prn/z-plane*), female subjects. Normotypical values of the angular measurement derived from the literature, expressed in degrees.

N	Mean	SD	Min	Max	Method	Population	Reference
40	3.4	1.3	-	-	direct anthropometry	North American White	[7]

Table S165: Deviation of the columella (*sn-c/z-plane*), male subjects. Normotypical values of the angular measurement derived from the literature, expressed in degrees.

N	Mean	SD	Min	Max	Method	Population	Reference
108	0.5	1.6	-	-	direct anthropometry	North American White	[7]

Table S166: Deviation of the columella (*sn-c/z-plane*), female subjects. Normotypical values of the angular measurement derived from the literature, expressed in degrees.

N	Mean	SD	Min	Max	Method	Population	Reference
199	0.4	1.7	-	-	direct anthropometry	North American White	[7]

Table S167: Inclination of the labial fissure (ch_r-ch_l/*x-plane*), male subjects. Normotypical values of the angular measurement derived from the literature, expressed in degrees.

N	Mean	SD	Min	Max	Method	Population	Reference
52	2	1.4	-	-	direct anthropometry	North American White	[7]

Table S168: Inclination of the labial fissure (ch_r-ch_l/*x-plane*), female subjects. Normotypical values of the angular measurement derived from the literature, expressed in degrees.

N	Mean	SD	Min	Max	Method	Population	Reference
51	3	-	-	-	direct anthropometry	North American White	[7]

Table S169: Inclination of the upper lip (*sn-ls/y-plane*), male subjects. Normotypical values of the angular measurement derived from the literature, expressed in degrees.

N	Mean	SD	Min	Max	Method	Population	Reference
109	1.5	8.7	-	-	direct anthropometry	North American White	[7]

Table S170: Inclination of the upper lip (*sn-ls/y-plane*), female subjects. Normotypical values of the angular measurement derived from the literature, expressed in degrees.

N	Mean	SD	Min	Max	Method	Population	Reference
199	0.8	7.5	-	-	direct anthropometry	North American White	[7]

Table S171: Inclination of the lower lip (*li-sl/y-plane*), male subjects. Normotypical values of the angular measurement derived from the literature, expressed in degrees.

N	Mean	SD	Min	Max	Method	Population	Reference
109	-46.6	17.8	-	-	direct anthropometry	North American White	[7]

Table S172: Inclination of the lower lip (*li-sl/y-plane*), female subjects. Normotypical values of the angular measurement derived from the literature, expressed in degrees.

N	Mean	SD	Min	Max	Method	Population	Reference
199	-48.6	14.1	-	-	direct anthropometry	North American White	[7]

Table S173: Nasal convexity angle (*sl-n-sn*), female subjects. Normotypical values of the angular measurement derived from the literature, expressed in degrees.

N	Mean	SD	Min	Max	Method	Population	Reference
12	11	2.3	-	-	computerized electromagnetic digitizer	Caucasian	[12]
12	9.6	2.7	-	-	computerized electromagnetic digitizer	Caucasian	[12]
71	10.9	2.7	-	-	computerized electromagnetic digitizer	Caucasian	[12]
66	9.3	2.0	5.0	15.1	3D facial scanner	Caucasian	[10]

Table S174: Variant of nasolabial angle (*prn-sn-ls*), female subjects. Normotypical values of the angular measurement derived from the literature, expressed in degrees.

N	Mean	SD	Min	Max	Method	Population	Reference
12	130.7	6.4	-	-	computerized electromagnetic digitizer	Caucasian	[12]
12	123	8.3	-	-	computerized electromagnetic digitizer	Caucasian	[12]
71	127.2	8	-	-	computerized electromagnetic digitizer	Caucasian	[12]
66	123.1	9.5	102.4	150.3	3D facial scanner	Caucasian	[10]
84	126.9	8.3	-	-	stereophotogrammetry	Caucasian Brazilian	[17]

Table S175: Deviation of the columella (*sn-c/z-plane*), female subjects. Normotypical values of the angular measurement derived from the literature, expressed in degrees.

N	Mean	SD	Min	Max	Method	Population	Reference
199	0.4	1.7	-	-	direct anthropometry	North American White	[7]

Table S176: Inclination of the labial fissure (ch_r-ch_l/*x-plane*), male subjects. Normotypical values of the angular measurement derived from the literature, expressed in degrees.

N	Mean	SD	Min	Max	Method	Population	Reference
52	2	1.4	-	-	direct anthropometry	North American White	[7]

Table S177: Inclination of the labial fissure (ch_r-ch_l/*x-plane*), female subjects. Normotypical values of the angular measurement derived from the literature, expressed in degrees.

N	Mean	SD	Min	Max	Method	Population	Reference
51	3	-	-	-	direct anthropometry	North American White	[7]

Table S178: Inclination of the upper lip (*sn-ls/y-plane*), male subjects. Normotypical values of the angular measurement derived from the literature, expressed in degrees.

N	Mean	SD	Min	Max	Method	Population	Reference
109	1.5	8.7	-	-	direct anthropometry	North American White	[7]

Table S179: Inclination of the upper lip (*sn-ls/y-plane*), female subjects. Normotypical values of the angular measurement derived from the literature, expressed in degrees.

N	Mean	SD	Min	Max	Method	Population	Reference
199	0.8	7.5	-	-	direct anthropometry	North American White	[7]

Table S180: Inclination of the lower lip (*li-sl/y-plane*), male subjects. Normotypical values of the angular measurement derived from the literature, expressed in degrees.

N	Mean	SD	Min	Max	Method	Population	Reference
109	-46.6	17.8	-	-	direct anthropometry	North American White	[7]

Table S181: Inclination of the lower lip (*li-sl/y-plane*), female subjects. Normotypical values of the angular measurement derived from the literature, expressed in degrees.

N	Mean	SD	Min	Max	Method	Population	Reference
199	-48.6	14.1	-	-	direct anthropometry	North American White	[7]

Table S182: Nasal convexity angle (*sl-n-sn*), female subjects. Normotypical values of the angular measurement derived from the literature, expressed in degrees.

N	Mean	SD	Min	Max	Method	Population	Reference
12	11	2.3	-	-	computerized electromagnetic digitizer	Caucasian	[12]
12	9.6	2.7	-	-	computerized electromagnetic digitizer	Caucasian	[12]
71	10.9	2.7	-	-	computerized electromagnetic digitizer	Caucasian	[12]
66	9.3	2.0	5.0	15.1	3D facial scanner	Caucasian	[10]

Table S183: Variant of nasolabial angle (*prn-sn-ls*), female subjects. Normotypical values of the angular measurement derived from the literature, expressed in degrees.

N	Mean	SD	Min	Max	Method	Population	Reference
12	130.7	6.4	-	-	computerized electromagnetic digitizer	Caucasian	[12]
12	123	8.3	-	-	computerized electromagnetic digitizer	Caucasian	[12]
71	127.2	8	-	-	computerized electromagnetic digitizer	Caucasian	[12]
66	123.1	9.5	102.4	150.3	3D facial scanner	Caucasian	[10]
84	126.9	8.3	-	-	stereophotogrammetry	Caucasian Brazilian	[17]

References

[1] Virgilio F. Ferrario, Chiarella Sforza, Carlo E. Poggio, and Graziano Serrao. Facial three-dimensional morphometry. *American Journal of Orthodontics and Dentofacial Orthopedics*, 109(1):86–93, 1996. doi: 10.1016/S0889-5406(96)70167-1.

[2] L. G. Farkas, M. J. Katic, C. R. Forrest, et al. International anthropometric study of facial morphology in various ethnic groups/races. *Journal of Craniofacial Surgery*, 16(4):615–646, 2005. doi: 10.1097/01.scs.0000171847.58031.9e.

[3] Y. F. Wen, H. M. Wong, R. Lin, G. Yin, and C. McGrath. Inter-ethnic/racial facial variations: A systematic review and bayesian meta-analysis of photogrammetric studies. *PLoS ONE*, 10(8):e0134525, 2015. doi: 10.1371/journal.pone.0134525.

[4] S. M. Weinberg, Z. D. Raffensperger, M. J. Kesterke, C. L. Heike, M. L. Cunningham, et al. The 3D facial norms database: Part 1. a web-based craniofacial anthropometric and image repository for the clinical and research community. *Cleft Palate-Craniofacial Journal*, 53(6):e185–e197, 2016. doi: 10.1597/15-199.

[5] Ö. Karaca, B. Gülcen, M. A. Kuş, F. Elmalı, and İ. Kuş. Morphometric facial analysis of turkish adults. *Balıkesir Sağlık Bilimleri Dergisi*, 1(1):7–11, 2012. URL `https://izlik.org/JA65AR34AX`.

[6] S. T. Ozdemir, D. Sigirli, I. Ercan, and A. S. Cakan. Photographic facial soft tissue analysis of healthy turkish young adults: Anthropometric measurements. *Aesthetic Plastic Surgery*, 33(2):175–184, 2009. doi: 10.1007/s00266-008-9274-z.

[7] L. G. Farkas. *Anthropometry of the Head and Face*. Raven Press, New York, USA, 2nd edition, 1994.

[8] M. G. Bozkir, P. Karakas, and O. Oguz. Vertical and horizontal neoclassical facial canons in turkish young adults. *Surgical and Radiologic Anatomy*, 26(3):212–219, 2004. doi: 10.1007/s00276-003-0202-2.

[9] S. Gündüz Arslan, C. Genç, B. Odabaş, and S. Kaya. Comparison of facial proportions and anthropometric norms among turkish young adults with different face types. *Aesthetic Plastic Surgery*, 32(2):234–242, 2008. doi: 10.1007/s00266-007-9049-y.

[10] L. M. Galantucci, R. Deli, A. Laino, E. Di Gioia, R. D'Alessio, F. Lavecchia, G. Percoco, and C. Savastano. Three-dimensional anthropometric database of attractive caucasian women: Standards and comparisons. *The Journal of Craniofacial Surgery*, 27(7):1884–1895, 2016. doi: 10.1097/SCS.0000000000002933.

[11] H. Jagadish Chandra, M. S. Ravi, S. M. Sharma, and B. Rajendra Prasad. Standards of facial esthetics: an anthropometric study. *Journal of Maxillofacial and Oral Surgery*, 11(4):384–389, 2012. doi: 10.1007/s12663-012-0355-9.

[12] C. Sforza, A. Laino, R. D'Alessio, G. Grandi, M. Binelli, and V. F. Ferrario. Soft-tissue facial characteristics of attractive italian women as compared to normal women. *The Angle Orthodontist*, 79(1):17–23, 2009. doi: 10.2319/122707-605.1.

[13] N. Winiarska, B. Roszkowski, W. Paskal, M. Majak, and P. Pietruski. The concept of ideal lips of caucasian female: An anthropometric analysis of the lower third of the face. *Aesthetic Plastic Surgery*, 49(1):20–30, 2025. doi: 10.1007/s00266-024-04299-1.

[14] G. Staka, M. Disha, and F. Dragidella. Cephalic and facial indices among kosovo-albanian population. *International Journal of Morphology*, 31(2):468–472, 2013. doi: 10.4067/S0717-95022013000200017.

[15] J. P. Porter. The average african american male face: An anthropometric analysis. *Archives of Facial Plastic Surgery*, 6(2):78–81, 2004. doi: 10.1001/archfaci.6.2.78.

[16] Leslie G. Farkas. *Anthropometry of the Head and Face in Medicine*. Elsevier North Holland, New York, USA, 1981.

[17] M. G. R. Mengoa, A. A. M. N. Garcia, K. S. Fioravanti, K. H. Neppelenbroek, T. M. Oliveira, C. Sforza, and S. Soares. Facial morphology analysis of Caucasian Brazilian adult women using stereophotogrammetry. *Brazilian Oral Research*, 38(e105):1–11, 2024. doi: 10.1590/1807-3107bor-2024.vol38.0105.

[18] Siddik Malkoç, Abdullah Demir, Tancan Uysal, and Naci Canbuldu. Angular photogrammetric analysis of the soft tissue facial profile of turkish adults. *European Journal of Orthodontics*, 31(2):174–179, 2009. doi: 10.1093/ejo/cjn082.

[19] Paula Fernández-Riveiro, Ernesto Smyth-Chamosa, David Suárez-Quintanilla, and Mercedes Suárez-Cunqueiro. Angular photogrammetric analysis of the soft tissue facial profile. *European Journal of Orthodontics*, 25(4):393–399, 2003. doi: 10.1093/ejo/25.4.393.

[20] Jr Sutter, R. E. and P. K. Turley. Soft tissue evaluation of contemporary caucasian and african american female facial profiles. *The Angle Orthodontist*, 68(6):487–496, 1998. doi: 10.1043/0003-3219(1998)068<0487:STEOCC>2.3.CO;2.

[21] Ahmet Uzun and Fikri Ozdemir. Morphometric analysis of nasal shapes and angles in young adults. *Brazilian Journal of Otorhinolaryngology*, 80(5):397–402, 2014. ISSN 1808-8694. doi: 10.1016/j.bjorl.2014.07.010.

[22] C. Sforza, G. Grandi, M. De Menezes, G. M. Tartaglia, and V. F. Ferrario. Age- and sex-related changes in the normal human external nose. *Forensic Science International*, 204(1-3):205.e1–205.e9, 2011. doi: 10.1016/j.forsciint.2010.07.027.

[23] Yan Gu, Jr McNamara, James A., Lauren M. Sigler, and Tiziano Baccetti. Comparison of craniofacial characteristics of typical chinese and caucasian young adults. *European Journal of Orthodontics*, 33(2):205–211, 2011. doi: 10.1093/ejo/cjq054.

[24] Tancan Uysal, Asli Baysal, Ahmet Yagci, Lauren M. Sigler, and Jr McNamara, James A. Ethnic differences in the soft tissue profiles of turkish and european–american young adults with normal occlusions and well-balanced faces. *European Journal of Orthodontics*, 34(3):296–301, 2012. doi: 10.1093/ejo/cjq165.
